# Final analysis of a phase II trial of daratumumab, carfilzomib, lenalidomide, and dexamethasone in newly diagnosed multiple myeloma without transplant

**DOI:** 10.1038/s41408-024-01045-3

**Published:** 2024-05-29

**Authors:** Benjamin A. Derman, Jennifer Cooperrider, Jacalyn Rosenblatt, David E. Avigan, Murtuza Rampurwala, David Barnidge, Ajay Major, Theodore Karrison, Ken Jiang, Aubrianna Ramsland, Tadeusz Kubicki, Andrzej J. Jakubowiak

**Affiliations:** 1 Section of Hematology/Oncology, University of Chicago, Chicago, IL USA; 2 Beth Israel Deaconess Medical Center, Boston, MA USA; 3 The Binding Site Group, part of Thermo Fisher, Rochester, MN USA; 4 University of Colorado, Denver, CO USA; 5 Department of Public Health Sciences, University of Chicago, Chicago, IL USA

**Keywords:** Phase II trials, Myeloma

## Abstract

We evaluated the efficacy and safety of 24 cycles of Dara in combination with carfilzomib (K), lenalidomide (R), and dexamethasone (d) without autologous stem cell transplant (ASCT) in newly diagnosed multiple myeloma (NDMM) irrespective of ASCT eligibility in a single-arm, phase II study. The primary endpoint was the rate of stringent complete response (sCR) and/or measurable residual disease (MRD) < 10^−5^ by next-generation sequencing (NGS) at the end of cycle 8 (C8). MRD was also assessed on peripheral blood samples using both the EXENT^®^ system and liquid chromatography–mass spectrometry (LC–MS). Forty-two patients entered the treatment phase; forty were evaluable for the primary endpoint. The rate of sCR and/or MRD < 10^−5^ following C8 was 30/40 (75%), meeting the statistical threshold for efficacy. The 10^−6^ MRD negative rate improved with treatment beyond C8. Agreement between EXENT^®^ and NGS was high and increased over time; agreement between LC-MS and NGS was lower. The estimated 3-year progression-free survival progression-free survival was 85%, and 3-year overall survival was 95%. Upper respiratory infections occurred in 67% (7% grade 3–4). There were no treatment-related deaths. Extended frontline Dara-KRd induced a high rate of sCR and/or MRD negativity; the rate and depth of MRD negativity improved beyond C8.

## Introduction

Patients with newly diagnosed multiple myeloma (NDMM) typically receive induction therapy consisting of at least a proteasome inhibitor (PI), an immunomodulatory imide drug (IMiD), and a corticosteroid, potentially followed by autologous stem cell transplant (ASCT) and maintenance therapy. ASCT employed in the frontline setting compared to a delayed ASCT at the time of first relapse has been shown to improve progression-free survival (PFS) but to date, its association with overall survival (OS) has not been demonstrated [1, 2].

Quadruplet induction therapy with the anti-CD38 monoclonal antibody (mAb) daratumumab (Dara) added to a PI, IMiD, and corticosteroid has led to high rates of deep and durable responses. The combination of Dara, bortezomib (V), lenalidomide (R), and dexamethasone (d) and ASCT led to a stringent complete response (sCR) rate of 69% and a minimal residual disease (MRD) negativity (10^−5^) rate of 75% as best response in the PERSEUS trial [3]. When V was replaced with carfilzomib (K) in the MASTER trial, MRD-adapted Dara-KRd with ASCT led to an MRD negative (10^−6^) rate of 71%; with rapid de-escalation of therapy for patients with MRD negativity, the 3-year PFS was 88%, 79%, and 50% for patients with 0, 1 or 2+ high-risk cytogenetic abnormalities (HRCA) [4]. An ASCT-free approach with Dara-KRd for up to 13 cycles led to a CR or better in 67% of patients; separately, the MANHATTAN trial found that Dara-KRd for 8 cycles led to an MRD negativity (10^−5^) rate of 71% [5, 6]. Some patients in both studies received post-protocol ASCT, and thus the durability of response to Dara-KRd without ASCT was not established.

In this phase II study, we sought to evaluate the efficacy of an ASCT-free approach with 24 cycles of Dara-KRd in patients with NDMM regardless of ASCT eligibility.

## Methods

### Study design and participants

This was an open-label, single-arm, phase II study that enrolled patients from two Multiple Myeloma Research Consortium sites in the United States. Patients aged 18 or older with NDMM were eligible irrespective of ASCT eligibility. All patients provided written informed consent; the study was conducted in accordance with the International Conference on Harmonization Guidelines for Good Clinical Practice and the Declaration of Helsinki. The study was approved by the institutional review boards of the participating institutions, and the study was registered at clinicaltrials.gov (NCT03500445). The datasets generated during the current study are available from the corresponding author upon reasonable request.

### Treatment

Patients with NDMM were permitted to receive up to one cycle of anti-myeloma therapy prior to enrollment. Once enrolled, patients received Dara-KRd for a planned 24 cycles at the following doses: intravenous (IV) daratumumab 16 mg/kg weekly for cycles (C) 1 & 2, every 2 weeks for C3–8, then every 4 weeks for C9–24; IV carfilzomib 20/36 mg/m^2^ on days 1, 2, 8, 9, 15, and 16 for C1–8 and then 36 mg/m^2^ on days 1, 2, 15, and 16 for C9–24; oral lenalidomide 25 mg on days 1–21 of a 28-day cycle for 24 cycles; and oral dexamethasone 40 mg weekly (20 mg if age > 75) for C1-8 and then 20 mg weekly for C9–24. Patients were given the option to harvest stem cells after 4–6 cycles of protocol therapy to permit ASCT in the future. Following the completion of 24 cycles of protocol therapy, single-agent lenalidomide maintenance therapy was recommended.

### Assessments

Minimal residual disease (MRD) testing was performed at the end of C8, C12, and C24 by next-generation sequencing (NGS, clonoSEQ, Adaptive Biotechnologies) with a limit of detection (LoD) of 6.8 × 10^−7^ with 20 µg of DNA input. Mass spectrometry (MS) of peripheral blood samples was also performed by The Binding Site (part of Thermo Fisher) using the matrix-assisted laser desorption/ionization time-of-flight (MALDI-TOF) MS-based EXENT^®^ system (lower limit of measuring interval (LLMI) = 15 mg/L in 200 mg/L total immunoglobulin) with EXENT^®^ immunoprecipitate eluates also analyzed by the more sensitive LC–MS (LLMI ≥ 0.15 mg/L) as previously described [7].

Adverse events (AEs) were graded according to the National Cancer Institute Common Terminology Criteria for Adverse Events version 4.0.

### Study endpoints

The primary endpoint was the rate of sCR and/or MRD negativity at the 10^−5^ threshold at the end of C8 to account for patients nonevaluable for MRD by NGS due to unavailable or untrackable clonal sequences along with the limitation of accurately distinguishing the IgG kappa mAb Dara from IgG kappa paraprotein by serum protein immunofixation. Secondary endpoints included conventional International Myeloma Working Group (IMWG) response [8], MRD status by NGS, toxicity, PFS, and OS. Peripheral blood MRD status using the EXENT^®^ assay and LC–MS were exploratory endpoints.

### Statistical analysis

The primary analysis required forty patients to test the null hypothesis that the sCR and/or MRD negative status by NGS at the end of 8 cycles is ≤30% against the alternative that it is >30% using an exact one-sided binomial test with alpha = 0.10. The null hypothesis was to be rejected if 17 or more sCR and/or MRD negative responses were observed. Our sample size provided 85% power if the true response rate was 50%.

Efficacy analyses were performed on the intent-to-treat (ITT) population, including the MRD negativity rate as per the international consensus on MRD reporting [9]. Patients without progression nor toxicity who discontinued treatment prior to C8 were included in all analyses except the C8 response evaluation; they were ‘replaced’ by additional patients to achieve the necessary sample size. Categorical data were compared using chi-squared or Fisher’s exact tests. The Kaplan-Meier method was used for time-to-event endpoints, specifically for PFS and OS.

## Results

### Patient characteristics

A total of 42 patients entered the treatment phase from March 2019 to January 2022. The data cutoff was July 15, 2023. The median age was 58 (range 39–79), including 12 (29%) age ≥ 65 years (Table 1). High-risk cytogenetic abnormalities (HRCA) by CD138+ selected *fluorescent in situ hybridization* according to IMWG criteria [10] were present in 24 (57%), including 10 (24%) with t(4;14), 3 (7%) with t(14;16), 8 (19%) with deletion 17p, and 14 (33%) with a 1q copy number abnormality (6 [14%] with 1q amplification); only 2 patients had an isolated 1q gain without any other high-risk abnormalities. Two or more HRCA were present in 10 (24%) patients.

**Table 1:** Baseline Characteristics.

Characteristic	*n* (%)
Total number of patients	42
*Age*	
Median (range)	58 (39-79)
≥65	12 (29%)
*Sex*	
Male	25 (60%)
Female	17 (40%)
*Race*	
White	23 (55%)
Black	13 (31%)
Asian	1 (2%)
More than one race	5 (12%)
*Ethnicity*	
Hispanic	5 (12%)
Non-Hispanic	37 (88%)
*ECOG Performance Status*	
0	31 (74%)
1	11 (26%)
*ISS/R-ISS Stage*	
I	21 (50%) / 14 (33%)
II	14 (33%) / 24 (57%)
III	7 (17%) / 4 (10%)
*IgG kappa monoclonal protein*	15 (36%)
*Extramedullary disease*	5 (12%)
*Cytogenetic risk by FISH*	
Unknown	1 (2%)
Standard	17 (41%)
High	24 (57%)
t(4;14)	10 (24%)
t(14;16)	3 (7%)
del(17p)	8 (19%)
1q copy number abnormalities^a^	14 (33%)
1q amplification	6 (14%)
2+ high-risk abnormalities	10 (24%)
*Number of cycles of therapy*	
Median (range)	23 (1–24)

*ECOG* Eastern Cooperative Oncology Group, *FISH* fluorescence in situ hybridization, *ISS* International Staging System.

^a^Eight patients had a 1q gain (no amplification). Of these 8 patients, only 2 had no other high-risk abnormalities.

**Fig. 1:**
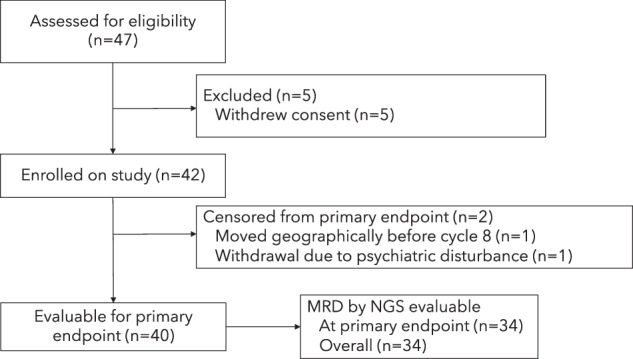
CONSORT diagram for enrolled patients. Forty-seven patients were assessed for eligibility, of which 42 enrolled on the study. A total of 40 patients were evaluable for the primary endpoint, though all 42 patients were followed for progression-free and overall survival.

Of the 42 patients who entered the treatment phase, 40 (95%) were evaluated for response at the end of C8. Two patients withdrew from the study before C8, one due to geographic relocation and one due to a psychological disturbance unrelated to treatment (Fig. 1). The median number of cycles received was 23 (range 1–24).

### Efficacy

Following 8 cycles of Dara-KRd (*n* = 40), the rate of sCR and/or MRD negativity (<10^−5^) was 30/40 (75%, 95% confidence interval [CI] 61–89%), meeting the statistical threshold for efficacy (Table 2). The overall response rate at the end of 8 cycles was 38/40 (95%), with 27 (68%) achieving an sCR, 28 (70%) at least a CR, and 38 (95%) at least a very good partial response. Two (5%) patients had primary refractory disease; one had two HRCA, and the other had an insufficient sample for cytogenetics but had 15% circulating plasma cells at diagnosis that had not met the previously established criteria (≥20% plasma cells) for plasma cell leukemia.

**Table 2:** Overall response and MRD-negativity rates.

Response	After 8 cycles, *n* (%)	Best response, *n* (%)
*Evaluable for response*	40	42
≥PR	38 (95%)	40 (95%)
≥VGPR	38 (95%)	40 (95%)
≥CR	28 (70%)	36 (86%)
sCR	27 (68%)	31 (74%)
PD	2 (5%)	2 (5%)
Early discontinuation	2 (5%)	–
Early death	2 (5%)	–
MRD by NGS		
MRD-negative (10^−^^5^)^a^	20/34 (59%)	22/34 (65%)
MRD-negative (10^−6^)^a^	12/34 (35%)	18/34 (53%)
sCR and/or MRD-negative (10^−5^) by NGS^a^	30/40 (75%)	32/42 (76%)
	(95% CI 61–89%)	(95% CI 60–88%)
Mass spectrometry		
EXENT^®^ negative^a^	22/39 (56%)	25/39 (64%)
LC-MS negative^a^	7/39 (18%)	12/39 (31%)
MRD Kinetics		
Sustained MRD-negative (10^−^^5^) by NGS^b^	N/A	11/27 (40%)
Converted After Cycle 8^c^		
MRD (+) 10^−^^5^ to MRD (−) 10^−^^5^	N/A	1/10 (10%)
MRD (+) 10^−^^6^ to MRD (−) 10^−6^	N/A	6/18 (30%)

^a^Patients with no clone ID or baseline mass spectrum are excluded from the denominator. Missing MRD or mass spectra results at a timepoint for those with a baseline are MRD positive.

^b^MRD < 10^−5^ on two or more instances at least one year apart. The denominator includes patients with trackable MRD and at least one year of MRD follow-up if they had at least one MRD negative result.

^c^Denominator includes patients still on protocol, with trackable MRD, and MRD-positive at the end of cycle 8.

*CR* complete response, *LC–MS* liquid chromatography–mass spectrometry, *MRD* measurable residual disease, *NGS* next generation sequencing, *PD* progressive disease, *PR* partial response, *sCR* stringent CR, *VGPR* very good partial response.

The rate of sCR as the best response in the ITT population (*n* = 42) was 31/42 (74%) and 36/42 (86%) ≥CR. A best response of sCR and/or MRD negativity (10^−5^) was achieved in 32/42 (76%).

### Patient disposition

Among the 42 patients who entered the treatment phase, 21 (50%) reached the end of therapy, 6 (14%) of whom were off all treatment at the data cutoff. An additional 11 (26%) patients remain on protocol treatment, all past C8. A total of 7 (17%) patients experienced disease progression, including 6 (14%) while on protocol therapy (Supplemental Tables 1 and 2). Two deaths occurred, both early in treatment and due to primary refractory disease. Stem cell collection was performed for 37 (88%) patients, all with G-CSF and upfront plerixafor with a median of 2 (range 1–3) days of collection, for a median yield of 8.26 × 10^6^ CD34^+^ cells/kg (range 3.1–17.5 × 10^6^ CD34^+^ cells/kg). Four (10%) patients discontinued protocol therapy early while still in disease response; one of these patients proceeded to ASCT (Supplemental Table 1). Another 3 (7%) patients received ASCT following disease progression.

### MRD by NGS

Clonotypic tracking for MRD by NGS was available for 34 (81%) patients; 4 (9.5%) had clonotypic tracking failure leading to a 90% calibration rate, and 4 (9.5%) had no suitable calibration material available (Supplemental Fig. 1). By MRD ITT [9], 20 (59%) achieved MRD negativity at the 10^−5^ threshold and 12 (35%) at 10^−6^ at the end of C8 (Table 2). Responses deepened over time (Table 2) among patients who reached C8: 1/10 (10%) and 6/18 (33%) patients converted from MRD positive at C8 to MRD negative at later timepoints at the 10^−5^ and 10^−6^ thresholds, respectively. The rate of MRD negativity at any timepoint was 22/34 (65%) at 10^−5^ and 18/34 (53%) at 10^−6^. Among 33 MRD-evaluable patients from C8 onward, 6 had one MRD < 10^−5^ result and less than one year of subsequent follow-up, while 11 of the remaining 27 patients (41%) had sustained 10^−5^ MRD negativity (two consecutive MRD negative results ≥1 year apart).

### Mass spectrometry

**Fig. 2:**
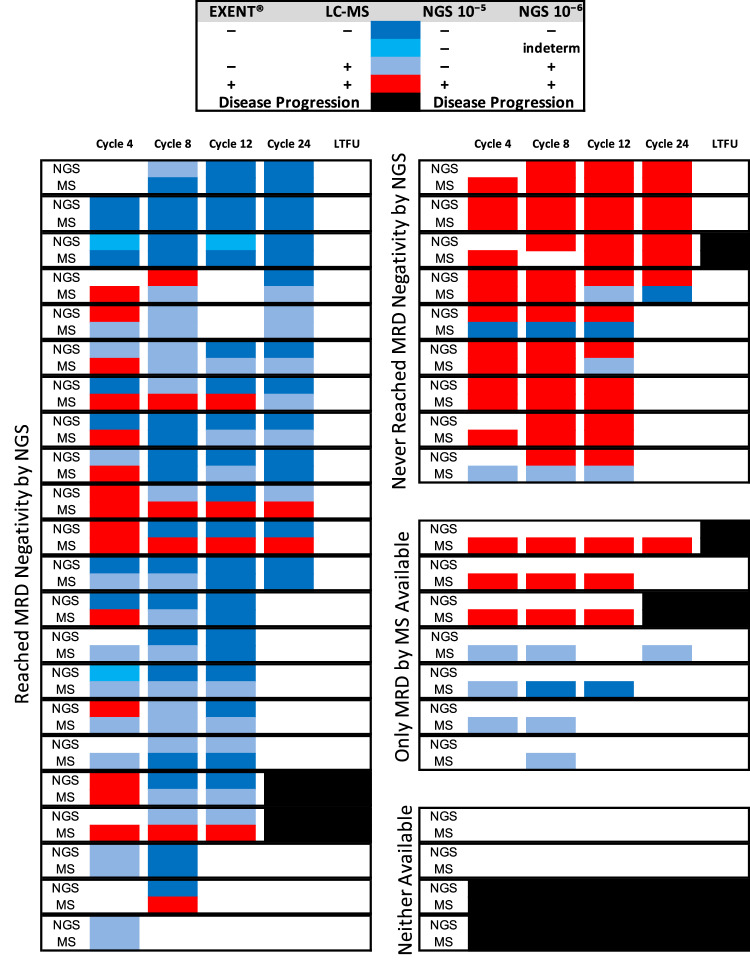
Kinetics of MRD by NGS and mass spectrometry. Longitudinal assessments for measurable residual disease using both next generation sequencing (NGS) and mass spectrometry (MS) are shown. Each paired row represents a unique patient. Black denotes patients with disease progression, including in long-term follow-up (LTFU). Four patients had neither NGS nor MS tracking results available, including two with primary refractory disease. indeterm indeterminate result, LC–MS liquid chromatography–mass spectrometry.

A trackable monoclonal light chain was identified in 39 (93%) patients; 3 patients had no baseline sample available (Supplemental Fig. 2). Among the same 39 patients evaluable for conventional response at C8, 22/39 (56%) were EXENT^®^ negative at the end of C8 and 25/39 (64%) were EXENT^®^ negative as the best response. By LC–MS, 7/39 (18%) were LC–MS negative following C8 and 12/39 (31%) were LC–MS negative as best response (Table 2; Fig. 2).

**Fig. 3:**
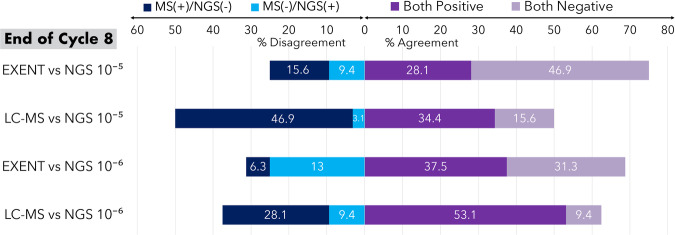
Agreement between mass spectrometry and next-generation sequencing following cycle 8 of Dara-KRd. A visual representation of concordance between mass spectrometry in the peripheral blood and next-generation sequencing in the bone marrow at the conclusion of 8 cycles of Dara-KRd. LC–MS liquid chromatography–mass spectrometry, MS mass spectrometry, NGS next-generation sequencing.

There was 75% agreement between 32 paired EXENT^®^ and NGS (10^−5^) samples at C8 (Cohen’s kappa 0.48, 95% CI 0.18–0.79), with 3 cases NGS(+)/EXENT^®^(−) and 5 cases NGS(−)/EXENT^®^(+) (Fig. 3). Among paired EXENT^®^ and NGS (10^−6^) samples at C8, the agreement was 69% (Cohen’s kappa 0.39, 0.10–0.69), with 8 cases NGS(+)/EXENT^®^(−) and only 2 cases NGS(−)/EXENT^®^(+). Concordance increased by the end of C24 to 84% for both EXENT^®^/NGS (10^−5^) and EXENT^®^/NGS (10^−6^).

The agreement between LC–MS and NGS at both thresholds was lower. There was 50% agreement (Cohen’s kappa 0.14, 95% CI: −0.08 to 0.36) between 32 paired LC–MS and NGS (10^−5^) samples at C8, with most discordance (15/16, 94%) due to NGS(−)/LC-MS(+) cases (Fig. 3). There was 63% agreement (Cohen’s kappa 0.11, 95% CI −0.21 to 0.43) between LC–MS and NGS (10^−6^) samples at C8, with 9 cases NGS(−)/LC-MS(+) and 3 cases NGS(+)/LC-MS(−). At the end of C24, 7/8 (88%) and 5/6 (83%) discordant cases were LC–MS(+)/NGS(−) at 10^−5^ and 10^−6^ thresholds, respectively.

### Progression-free survival and overall survival

**Fig. 4:**
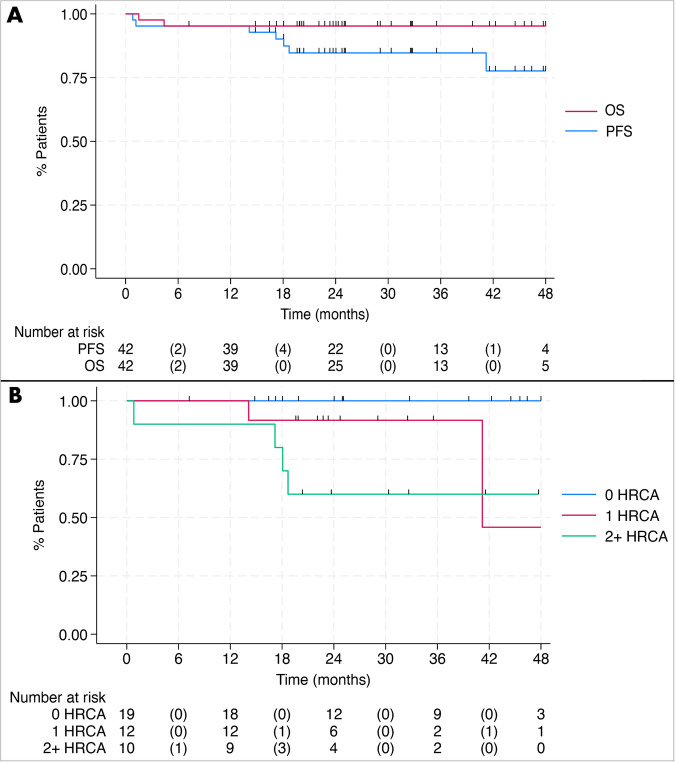
Progression-free survival and overall survival. **A** Progression-free survival (PFS) and overall survival (OS) by intent to treat analysis. **B** PFS is stratified by the number of high-risk cytogenetic abnormalities (HRCA). One patient had unknown cytogenetics.

With a median follow-up of 27 months (range 1.5–52 months), there were 7 progression events and 2 deaths (both due to progression). The estimated 3-year PFS was 85% (Fig. 4): 100% for standard-risk disease, 92% for 1 HRCA, and 60% for 2 + HRCA. Of the 7 patients with progression, 6 had at least one of the following: extramedullary disease (*n* = 4), 2 + HRCA (*n* = 4), or circulating plasma cells (*n* = 1). The estimated 3-year OS was 95%.

Excluding patients who experienced progression or death prior to C8 using the landmark method [11], C8 MRD status at neither 10^-5^ nor 10^−6^ was associated with PFS (Supplemental Fig. 3). None of the 11 patients with sustained MRD negativity < 10^−5^ had disease progression.

**Fig. 5:**
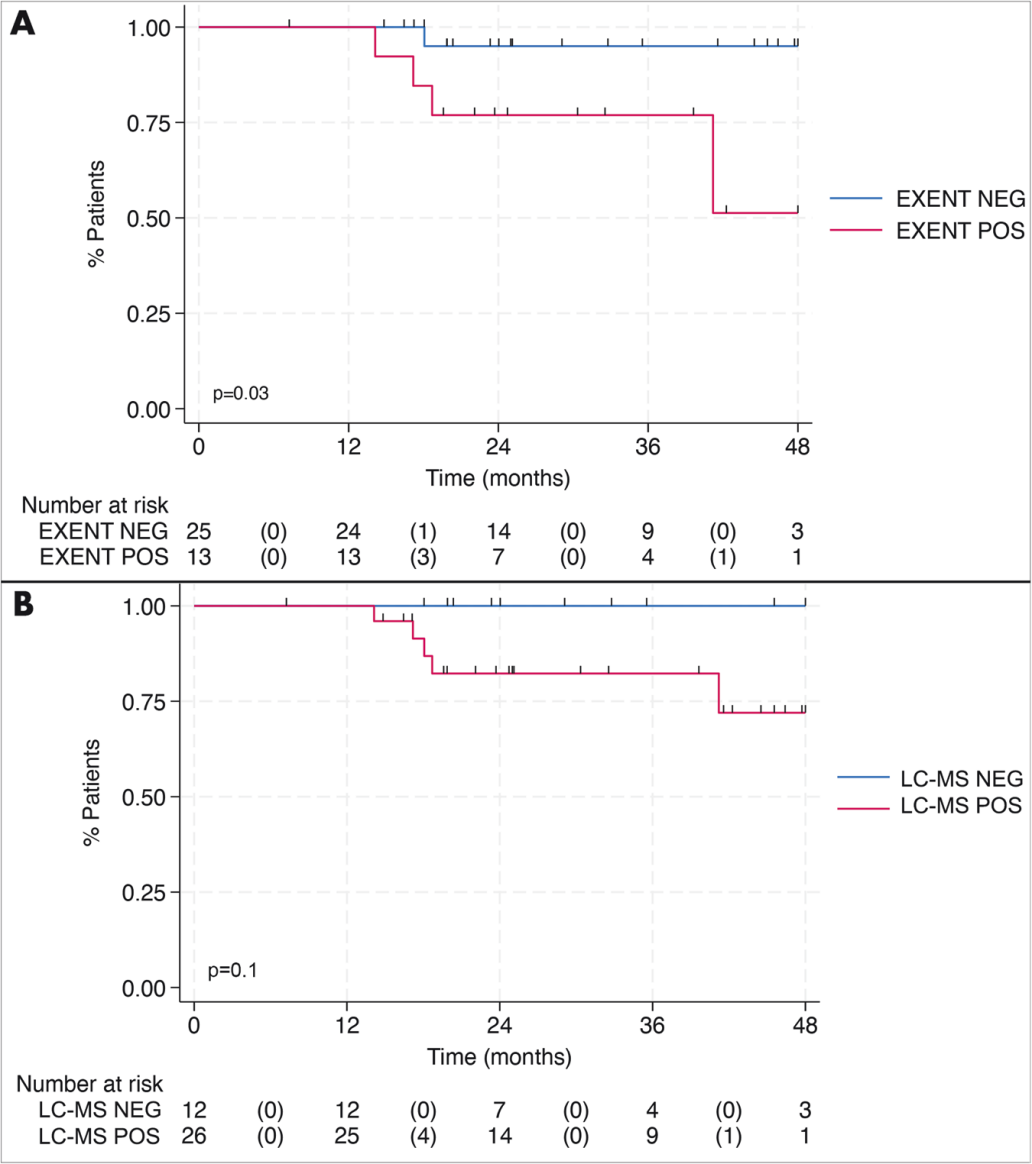
Progression-free survival stratified by mass spectrometry status as best response. **A** Progression-free survival stratified by EXENT status as the best response and **B** progression-free survival stratified by liquid-chromatography mass spectrometry (LC–MS) status as the best response, both using the landmark method from cycle 8 onward.

Using the same landmark method [11], the EXENT^®^ assay and LC-MS status at C8 were not associated with PFS (logrank *p* = 0.055 and *p* = 0.22, respectively) (Supplemental Fig. 4). Excluding patients with progression before C8, EXENT^®^ negative status as the best response was associated with superior PFS (*p* = 0.03); only one patient with EXENT^®^ negativity had disease progression. None of the patients who reached LC–MS negativity experienced disease progression (Fig. 5).

### Safety and tolerability

Dose reductions occurred in 2 (5%) patients for daratumumab, both as temporary dose omissions to limit exposure at the onset of the COVID-19 pandemic. Dose reductions occurred in 11 (26%) patients for carfilzomib, 23 (55%) for lenalidomide, and 22 (52%) for dexamethasone. Hematologic AEs (Table 3) included neutropenia (all grade (G) 26%, G3 + 21%), anemia (G 59%, G3 + 2%), and thrombocytopenia (G 64%, G3 + 26%). The most common nonhematologic AEs were hyperglycemia (G 76%, G3 + 7%), diarrhea (G 71%, G3 + 5%), hypertension (G 57%, G3 + 17%), and neuropathy (G 40%, G3 + 0%). Upper respiratory infections occurred in 28 (67%) patients including 16 (38%) with COVID-19 infections; there were 3 (7%) total G3+ infectious events. There was one case of thrombotic microangiopathy leading to the discontinuation of carfilzomib. G3 atrial fibrillation and heart failure each occurred in 1 patient, both of which resolved. No patient discontinued treatment or died due to toxicity.

**Table 3:** Treatment-emergent adverse events during Dara-KRd.

Adverse event	All grades, *n* (%) (*n* = 42)	Grade 3+, *n* (%) (*n* = 42)
*Hematologic*		
Lymphopenia	29 (69%)	15 (36%)
Thrombocytopenia	27 (64%)	11 (26%)
Anemia	25 (59%)	1 (2%)
Neutropenia	11 (26%)	9 (21%)
*Non-hematologic*		
Fatigue	37 (88%)	1 (2%)
*Infection*		
Upper respiratory^a^	28 (67%)	3 (7%)
Skin/Soft Tissue	5 (12%)	1 (2%)
Urinary tract	2 (5%)	0 (0%)
Other^b^	9 (21%)	0 (0%)
Hyperglycemia	32 (76%)	3 (7%)
Diarrhea	30 (71%)	2 (5%)
Lower extremity edema	28 (67%)	0 (0%)
Dyspnea	26 (62%)	0 (0%)
Hypertension	24 (57%)	7 (17%)
Liver enzyme elevations	17 (40%)	4 (10%)
Peripheral sensory neuropathy	17 (40%)	0 (0%)
Nausea	15 (35%)	1 (2%)
*Cardiac events, any*		
Chest pain	3 (7%)	0 (0%)
Atrial fibrillation	2 (5%)	1 (2%)
Sinus bradycardia	4 (10%)	0 (0%)
Sinus tachycardia	3 (7%)	0 (0%)
Reduced ejection fraction	4 (10%)	1 (2%)
*Electrolyte imbalances, any*		
Hyperkalemia	5 (12%)	1 (2%)
Hypocalcemia	13 (31%)	2 (5%)
Hypokalemia	23 (55%)	4 (10%)
Hypomagnesemia	5 (12%)	0 (0%)
Hypophosphatemia	9 (21%)	1 (2%)
Hyponatremia	4 (10%)	0 (0%)
Rash	13 (31%)	2 (5%)
Blurred vision	11 (26%)	0 (0%)
Acute kidney injury^c^	8 (19%)	2 (5%)
Infusion reactions	4 (10%)	0 (0%)

^a^Includes 16 patients with COVID-19 infection (1 grade 3 event).

^b^3 gastrointestinal infections, 2 fungal rashes, 2 mucositis, 1 bacterial pneumonia, 1 ear infection.

^c^Includes one case of thrombotic microangiopathy.

## Discussion

In this phase II study involving a diverse NDMM population with 57% harboring an HRCA, eight cycles of Dara-KRd without ASCT led to an sCR and/or MRD negativity rate of 75%, thereby meeting the primary endpoint. The historical comparator for this trial was KRd without ASCT, which led to sCR rates ranging widely from 6 to 62% in a variety of settings [12–15]. Extended Dara-KRd also compares favorably with other quadruplets; the sCR and/or MRD negativity (<10^−5^) rate after 8 cycles of elotuzumab (Elo)-KRd was 58% [16]. Even with the more conservative ITT approach, the 59% post-induction MRD 10^−^^5^ negativity rate in this study was high in the context of other (sometimes shorter) quadruplet induction strategies: Dara-VRd 22% [17], Dara-VMP 28% [18], Dara-VTd 35% [19], Dara-KRd 38% [20], Isa-VRd 50% [21], Isa-KRd 45-54% [22, 23], and Elo-KRd 53% [24].

The estimated 3-year PFS with Dara-KRd in this trial was 85%, including 100% for patients with no HRCA, 92% for 1 HRCA, and 60% for 2 + HRCA. This represents one of the highest reported rates of 3-year PFS in NDMM regardless of receipt of ASCT; for comparison, in the phase 3 PERSEUS trial, Dara-VRd and ASCT led to a 3-year PFS of 90% [3]. This study also highlights the challenge of treating patients with 2 + HRCA. A post-hoc analysis of PFS from the GRIFFIN trial revealed the 3-year PFS with Dara-VRd and ASCT to be 54% for patients with 2 + HRCA [25]. The MASTER trial involving Dara-KRd, ASCT, and MRD-guided discontinuation of therapy and the GMMG-CONCEPT trial involving extended Isa-KR post-ASCT led to a similar 3-year PFS.^4,22^A retrospective study of patients with 2 + HRCA who received ASCT was less optimistic with a median PFS of just 22.9 months [26]. The OPTIMUM MUKnine trial used an extended high-intensity therapy approach for ultra-high-risk disease, yielding a 3-year PFS of ~75% [27]. The totality of these results suggests that the current treatment approach for patients with 2 + HRCA is unsatisfactory regardless of ASCT and serves as motivation to incorporate chimeric antigen receptor (CAR) T-cell therapy and bispecific antibodies earlier into the treatment paradigm.

Extended quadruplet therapy may help compensate for the deferral of ASCT. Eight cycles of induction have been an arbitrary historical standard for patients deferring ASCT. The benefit of extending quadruplet therapy beyond 8 cycles is further exemplified by the deepening of responses over time in this study and in others [13, 28, 29]. The rate of MRD negativity at the 10^−6^ threshold was 35% after 8 cycles of therapy but increased to 53% as a best response, which may explain why C8 MRD status was not prognostic. Importantly, no patient with sustained MRD < 10^−5^ nor <10^−^^6^ experienced disease progression, in line with observations that sustained MRD negativity is an important prognostic marker.

Stem cell mobilization and collection did not appear to be significantly impacted by the administration of a quadruplet therapy in this study. The median stem cell yield in this study was higher than in the MASTER trial (8.26 × 10^6^ vs 6.0 × 10^6^ CD34^+^ cells/kg), perhaps because mobilization of all patients in our study included upfront plerixafor and because a higher target stem cell dose (4 × 10^6^ CD34^+^ cells/kg per transplant) was used.

Mass spectrometry performed on peripheral blood also added prognostic value. Negativity as the best response using EXENT^®^ was associated with superior PFS, and patients negative by LC–MS did not experience disease progression. The sensitivity of LC–MS is greater than EXENT^®^, which explains the lower rate of LC–MS negativity compared to EXENT^®^ negativity; a negative LC–MS result may serve as one of the best predictors of durable response. However, C8 does not appear to be the appropriate timepoint for prognostication by mass spectrometry, particularly for patients with an IgG heavy chain, likely due to paraprotein that persists in circulation long after eradication of the malignant plasma cells that produced it [30]. A recent analysis among patients receiving maintenance therapy found that 18 months post-ASCT was the optimal prognostication timepoint for EXENT^®^ [31]. Additionally, identifying the time from immunofixation negativity to negativity by mass spectrometry for each method is an area requiring further research.

No patient discontinued protocol therapy due to toxicity, suggesting extended Dara-KRd has a favorable safety profile. The 55% dose reduction rate for lenalidomide is similar to that seen in the ENDURANCE trial [14]. Rates of neutropenia were low, yet infections were still common, potentially in part due to enrollment of patients primarily during the initial years of the COVID-19 pandemic; while 38% of patients did contract COVID-19, no patient died from COVID-19. Cardiac events were rare, and no venous thromboembolic (VTE) events were reported, potentially due to omitting intravenous fluids with carfilzomib after cycle 1 day 1, close collaboration with a cardio-oncologist, and the recommendation for enhanced VTE prophylaxis with direct oral anticoagulants. The absence of treatment-related deaths in this study is notable in light of preliminary findings from the GEM2017FIT study, which included a cohort of older transplant-ineligible patients that were assigned 18 cycles of Dara-KRd, of which 8.5% died due to an adverse event [32]. In a similar vein, the PERSEUS study found that compared with VRd and ASCT, Dara-VRd and ASCT were associated with improved PFS in nearly all subgroups except patients aged 65 and older. These studies may suggest that quadruplet therapy be used with caution in older adults.

Limitations of this study include its small sample size and the use of a nonstandard primary endpoint. Twice weekly carfilzomib, as used in this study, has become less common in practice, especially as Dara-KRd with once weekly carfilzomib 56 mg/m^2^ led to similarly high MRD negativity rates [6]. Extending Dara-KRd carries cost implications and time toxicity to patients but may also spare patients from frontline ASCT that is associated with short-term detriments in health-related quality of life and increased risk of second hematologic malignancies [33, 34]. Patients who experience relapse while on Dara-KRd face more challenges in finding a suitable second-line regimen, as it is unlikely that bortezomib has activity in carfilzomib-resistant settings.

In conclusion, this study is the first to our knowledge to show that extended Dara-KRd can induce and sustain deep and durable responses without the use of ASCT. The high proportion of patients with HRCA in this study provides a signal of efficacy in a population that is typically recommended to proceed with early ASCT when possible. The success of this extended approach may be due to the deepening of MRD and MS responses beyond eight cycles, raising the question of whether ASCT could be supplanted in NDMM. Moreover, this study challenges whether the addition of an anti-CD38 mAb to KRd may lead to superior outcomes compared to KRd with or without ASCT. The phase III PERSEUS study that compared Dara-VRd vs VRd in the ASCT-intended setting has shown that Dara-VRd is associated with superior PFS [3]. An ongoing phase III randomized trial of VRd vs extended KRd (NCT03729804) may provide a signal about the duration of PI and outcomes, and the phase III CEPHEUS and IMROZ studies may further inform on the best approach to NDMM in the transplant-deferred setting. Still, other randomized studies will be needed to answer whether ASCT can be circumvented by extending quadruplet treatment for most patients with NDMM and whether extended quadruplet therapy is superior to shorter courses of quadruplet therapy in the absence of ASCT.

### Supplementary information

Study Protocol

Supplemental material

## Data Availability

The data generated in this study is available from the corresponding author upon reasonable request.
