# Supplementary material for: Final analysis of a phase II trial of daratumumab, carfilzomib, lenalidomide, and dexamethasone in newly diagnosed multiple myeloma without transplant
Source: Blood Cancer J. 2024 May 29;14(1):87. doi: 10.1038/s41408-024-01045-3 (PMC11136961; doi:10.1038/s41408-024-01045-3)
Supplement: Supplementary file 1 — Study Protocol [file 41408_2024_1045_MOESM1_ESM.pdf]

CLINICAL STUDY PROTOCOL

**Protocol Number:**	UChicago Protocol IRB17-1097
[redacted]	[redacted]
**Study Title:**	Open-Label, Single-Arm, Phase 2 Study of Initial Treatment with Daratumumab (Darzalex), Carfilzomib (Kyprolis), Lenalidomide (Revlimid) and Low-Dose Dexamethasone (DKRd) in Newly Diagnosed, Multiple Myeloma Requiring Systemic Chemotherapy
**Sponsor:**	The University of Chicago
**Lead Principal Investigator:**	Andrzej Jakubowiak, MD, PhD<br>[redacted]
**Co-Investigator:**	Benjamin Derman, MD<br>[redacted]
**Investigational Products:**	Drug Company Supplied:<br>Carfilzomib (Kyprolis®) (Amgen)<br>Daratumumab (Darzalex®) (Janssen)<br>Lenalidomide (Revlimid®) (Celgene)
**Type Of Research:**	Interventional, clinical multi-center
**Protocol Version:**	Protocol Version Date: February 20, 2023 (Version 7)

DOCUMENT HISTORY

Use the table below to summarize modifications and amendments to protocol that have been approved by the Study Lead PI. Note: The protocol must not be rewritten or modified by anyone other than the Study Lead PI.

**Protocol Version Date**	**Description**
February 20, 2023	Amended protocol for IRB submission. Please reference Summary of Changes (Document Title: "IRB17-1097_SOC_02.20.2023") for summary of edits to Protocol Version Date: 02/20/23.
1.0 dated 29 JAN2018<br>2.0 dated 26 OCT2018<br>3.0 dated 15 FEB2019<br>4.0 dated 5 APR 2019<br>5.0 dated 18 SEPT 2019<br>6.0 dated 11Nov2019	Previous versions of protocol.

Table of Contents

LIST OF ABBREVIATIONS AND DEFINITIONS OF TERMS 11

1. INTRODUCTION 14

2 STUDY ENDPOINTS 30

2.1 Primary Objective 29

2.2 Secondary Objectives 29

2.3 Exploratory Objectives 29

3 STUDY ENDPOINTS 30

3.1 Primary Endpoints 30

3.2 Secondary Endpoints 30

3.3 Tertiary/Exploratory Endpoints 30

4 INVESTIGATIONAL PLAN 30

4.1 Overview of Study Design and Dosing Regimen 30

5 PATIENT SELECTION 33

5.1 Inclusion Criteria 33

5.2 Exclusion Criteria 35

5.3 Subject Enrollment and Registration 37

5.3.1 Registration Process 37

6 TREATMENT PLAN 39

6.1 Study Procedures 39

6.2 Pretreatment Preparation 44

6.3 Study Drug Administration 49

6.4 Instructions for Initiation of a New Cycle 55

6.5 Dose-modification guidelines 56

6.6 Treatment Discontinuation 70

6.7 Safety Considerations & Supportive Care 72

7 SAMPLES FOR MRD EVALUATION AND CORRELATIVE SAMPLES 74

8 ADVERSE EVENTS ... 78
8.1 Causality ... 79
8.2 Adverse Event Reporting Procedures ... 80
8.3 Serious Adverse Events ... 81
9 INVESTIGATIONAL MEDICINAL PRODUCT ... 98
9.1 Carfilzomib ... 98
9.2 Lenalidomide ... 99
9.3 Dexamethasone ... 100
10 STATISTICAL CONSIDERATIONS ... 102
10.1 Objectives ... 102
10.2 Sample Size Justification & Analysis Plan ... 113
11 DATA REPORTING ... 106
12 REGULATORY OBLIGATIONS ... 106
12.1 Informed Consent ... 106
12.2 Compliance with Laws and Regulations ... 107
12.3 Subject Confidentiality ... 108
12.4 Multicenter Guidelines ... 109
13 ADMINISTRATION AND LEGAL OBLIGATIONS ... 109
13.1 Institutional Review Board (IRB) Approval and Consent ... 109
13.2 Required Documentation ... 110
13.3 Protocol Amendments and Study Termination ... 110
13.4 Study Documentation and Archive ... 111
13.5 Clinical Monitoring Procedures ... 113
13.6 Data Safety and Monitoring ... 114
13.7 Quality Assurance & Auditing ... 116
REFERENCES ... 116
APPENDIX 1: MULTIPLE MYELOMA STAGING ... 122

APPENDIX 2: ECOG PERFORMANCE STATUS 123

APPENDIX 3: NCI CTCAE VERSION 4.0 124

APPENDIX 4: RESPONSE CRITERIA FOR MULTIPLE MYELOMA 125

APPENDIX 5: FACT/GOG-NEUROTOXICITY QUESTIONNAIRE, V. 4.0........ 128

APPENDIX 6: QUALITY OF LIFE ASSESSMENT TOOL 129

APPENDIX 7: REVLIMID PREGNANCY RISK MINIMIZATION PLAN 132

APPENDIX 8: SCHEDULE OF EVENTS........ 146

SYNOPSIS

<table>
<tr><td>Objectives</td><td>Primary Objective
The primary objective of this study is to evaluate the rate of sCR and the rate MRD-negative disease by NGS at the end of 8 cycles.

Secondary Objectives
Secondary objectives include characterization of efficacy variables:
Rate of MRD by next generation gene sequencing (NGS) by clonoSEQ (Adaptive Biotechnologies) at the end of Cycles 8 and 24, and then yearly for as long as no PD for a maximum follow-up period of 2 years.
Duration of response (DOR), progression-free survival (PFS), time to progression (TTP), and overall survival (OS).
Overall response rate defined as partial response or better (>PR) including the rate of VGPR or better (>VGPR) and near complete response or better (sCR/CR/nCR) across entire treatment in high-risk and low-risk patients at indicated time points and as best response.
Safety and tolerability of regimen.
Exploratory Objectives
GEP, proteomics, RNASeq, and gene sequencing studies will be conducted on pro-treatment patient samples to evaluate the correlation between treatment outcome and pre-treatment patient profile.</td></tr>
<tr><td>Sample Size</td><td>A total of 45 non-transplant candidates and/or transplant candidates who agreed to defer transplant will be enrolled.</td></tr>
<tr><td>Study Design</td><td>This study will be a multi-center, open-label, phase 2 study, enrolling subjects with newly diagnosed MM who require systemic chemotherapy.

Dose Schedule and Dose Levels
Dosing schedule is based on 28-day cycles.

<u>Daratumumab</u>
Daratumumab (1800 mg) will be administered by SC injection by manual push over approximately 3 – 5 minutes in the abdominal subcutaneous tissues in the left/right locations, alternating between individual doses. The volume of the SC solution will be 15 mL for the 1800 mg dose.
Cycles 1-2: Days 1, 8, 15, and 22
Cycles 3-8: Days 1 and 15
Cycles 9-24: Day 1
<u>Carfilzomib</u>
- Cycle 1: Days 1 and 2: 20 mg/m^2 IV infusion</td></tr>
</table>

	- Cycle 1: Days 8, 9, 15, and 16: 36 mg/m^2<br><br>*Alternatively, intermediate dose escalation (to 27mg/m^2 on Days 8, 9 of Cycle 1) will be allowed at the treating physician's discretion.<br><br>- Cycle 2-8 36 mg/m^2 (or last best tolerated dose) of carfilzomib on Days 1, 2, 8, 9 and 15, 16<br><br>- Cycle 9-24: 36 mg/m^2 (or last best tolerated dose) of carfilzomib on Days 1, 2 and 15, 16<br><br>**Lenalidomide**<br>- Cycle 1-24 PO at 25 mg per dose (or at last best tolerated dose) on Days 1- 21<br><br>**Dexamethasone**<br>Dexamethasone will be administered between 30 minutes and 4 hours preceding carfilzomib (on days that they coincide), as follows:<br><br>• Cycles 1-4: 40 mg PO (subjects $<$ 75 years) or 20 mg PO (subjects $\geq$ 75 years) per week<br><br>• Cycles 5-24: 20 mg PO per week<br><br>For weeks of daratumumab injection, dexamethasone dose is split into two doses: half the dose prior to daratumumab infusion and half the dose the day after.<br><br>Split weekly dosing on other days (e.g. 10 mg on Day 1 and 10 mg on Day 2, etc.) is permitted with approval from Lead Principal Investigator.<br><br>Dexamethasone given on days without carfilzomib (on Days 22 and 23 of Cycles 1-8) may be self-administered by the subject on an outpatient basis. If Day 2 of carfilzomib dosing is delayed (i.e., Day 2, 9, 16) 4 mg of dexamethasone premedication is required to be used prior to second treatment. Missed doses will not be replaced during a cycle.<br>Missed doses of dexamethasone will not be made up.<br><br>**Treatment Overview**<br>Cycles 1 through 4 are considered D-KRd induction cycles, Cycle 5-8 are D-KRd consolidation, and cycles 9-24 D-KRd are maintenance cycles.<br><br>After Cycle 4, subjects who are deemed candidates for ASCT will proceed to stem cell harvest as per institutional guidelines. However, these subjects will not proceed straight to ASCT and will have cells frozen down for a potential future transplant at progression or unacceptable toxicity after

	stem cell harvest. After completion of stem cell harvest, subjects will resume protocol treatment.<br><br>Subjects who complete 24 months of D-KRd will then proceed to lenalidomide single-agent maintenance at least tolerated dose on Days 1-21 in 28-day cycles.<br><br>All subjects will undergo testing for minimal residual disease (MRD) using next generation sequencing (NGS) using clonoSEQ with 10-6 sensitivity at the end of Cycles 8 and 24. After Cycle 24, testing will continue on a yearly basis for as long as the subject is free of progression for a maximum follow-up period of 2 years. If a CR is suspected after Cycle 8, subjects will undergo MRD testing at Cycle 12 and/or 18 as well.
**Duration of Treatment**	The total study enrollment period is expected to be 12-18 months. A subject is considered to have completed the treatment phase of the study 4 weeks (28 days) after the end of the last treatment cycle. Excluding long-term follow-up, the treatment phase of the study will be completed once the last subject completes treatment the 28-day safety follow-up visit. Subjects who have not progressed will be followed for progression for up to 2 years from the first patient enrollment.
**Key Inclusion/Exclusion Criteria**	**<u>Inclusion Criteria</u>**<br><br>**Disease-related:**<br><br>1. Newly diagnosed, previously untreated myeloma requiring systemic chemotherapy.<br><br>Prior treatment of hypercalcemia or spinal cord compression or active and/or aggressively progressing myeloma with corticosteroids or lenalidomide or bortezomib-based regimens does not disqualify the patient (the treatment dose should not exceed the equivalent of 160 mg of dexamethasone in a 4 week period or not more than 1 cycle of PI/IMiD-based therapy).<br><br>2. Both transplant and non-transplant candidates are eligible.<br><br>3. Diagnosis of symptomatic multiple myeloma as per current IMWG uniform criteria prior to initial treatment.<br><br>4. Monoclonal plasma cells in the BM ≥ 10% or presence of a biopsy-proven plasmacytoma.<br><br>5. Measurable disease, prior to initial treatment including any emergent treatment of myeloma. Measurable disease will be indicated by one or more of the following:<br>• Serum M-protein ≥ 1 g/dL<br>• Urine M-protein ≥ 200 mg/24 hours

	• If serum protein electrophoresis is felt to be unreliable for routine M-protein measurement, then quantitative immunoglobulin levels are acceptable<br>• Serum Freelite measurable disease as per current IMWG criteria<br><br>**<u>Exclusion Criteria</u>**<br><br>**Disease-related:**<br><br>1. Frail non-transplant candidates, defined as in Palumbo et al, Blood 2015.<br><br>2. Non-secretory or hyposecretory multiple myeloma, prior to initial treatment defined as <1.0 g/dL M-protein in serum, <200 mg/24 hr urine M-protein, and no measurable disease as per IMWG by Freelite.<br><br>3. POEMS syndrome (polyneuropathy, organomegaly, endocrinopathy, monoclonal protein, and skin changes).<br><br>4. Amyloidosis<br><br>5. Plasma cell leukemia<br><br>6. Waldenström's macroglobulinemia or IgM myeloma<br><br>7. Radiotherapy to multiple sites or immunotherapy within 4 weeks before start of protocol treatment (localized radiotherapy to a single site at least 1 week before start is permissible).<br><br>8. Participation in an investigational therapeutic study within 3 weeks or within 5 drug half-lives (t ½) prior to first dose, whichever time is greater.
**Response**	M-protein determination:<br>• Serum Protein Electrophoresis (SPEP) and immunofixation<br>• Urine Protein Electrophoresis (UPEP) and immunofixation<br>• Serum Free Light Chains (SFLC) and Hevylite test<br>• Serum quantitative immunoglobulins (Igs)<br><br>• Bone marrow biopsy is required to confirm response at time of suspected CR or better.<br>• MRD by gene sequencing (NGS)<br>• Plasmacytoma evaluation should be completed if clinically indicated
**Safety Variables & Analysis**	The safety and tolerability of daratumumab, lenalidomide and carfilzomib will be evaluated by means of drug-related AE reports, physical examinations, and laboratory safety evaluations. Common Terminology Criteria for Adverse Events (CTCAE) v4.0 will be used for grading of AEs. Treating investigators will provide their assessment of causality as 1) unrelated, 2) unlikely related 3) possibly related, or 4) probably or 5) definitely related for all AEs.

**Statistical Analysis**	A total of 45 non-transplant candidates and/or transplant candidates who agreed to defer transplant will be enrolled. The sample size is selected to allow for a collection of sufficient data for evaluation of sCR and MRD rates at the end of 8 cycles, which assumes that we may not have successful MRD evaluation in up to 25% of patients (primary end-point), and that up to 15% transplant candidates may change their mind and want to proceed to transplant, both based on our historical experience in our prior KRd studies and is based on historical rates of sCR from KRd study without ASCT (or deferred transplant) of 30% at the end of 8 cycles, and preliminary rates of sCR and MRD by NGS at the end of Cycle 8 of 69% and 66%, respectively (both to be updated at ASH 2016). Primary analysis will require 50+ non-transplant candidates and/or transplant candidates who agreed to defer transplant to test the null hypothesis that the sCR and/or negative MRD status by NGS rate after 8 cycles is, ≤30% against the alternative that it is,>30% using an exact one-sided binomial test at the alpha=0.10 significance level. The null hypothesis will be rejected if 17 or more sCR's and/or MRD-negative's responses are observed (>42.5%). The sample size of n=45 response evaluable subjects provides a little over 85% power if the true sCR and/or negative MRD status rate by NGS is 50%. Based on this analysis, the study will provide preliminary estimate of probability of improvement of outcome of treatment with KRd without ASCT or with deferred ASCT for transplant candidates. In addition, we will conduct secondary analysis of sCR and/or MRD rates at the end of 8 cycles to test the null hypothesis that the sCR and/or negative MRD status by NGS rate after 8 cycles is, ≤30% against the alternative that it is, >60% using an exact one-sided binomial test at the alpha=0.10 significance level. The sample size of n=45 response evaluable subjects provides a little over 99% power if the true sCR and/or negative MRD status rate by NGS is 60%. Based on this analysis, the study will provide preliminary estimate of probability of higher similar efficacy of D+KRd to KRd+ASCT. The sample size is selected to also allow for collection of sufficient data for toxicity and tolerability evaluation of the D-KRd regimen. The frequency of adverse events will be summarized by type, grade, and attribution to the study drugs.

LIST OF ABBREVIATIONS AND DEFINITIONS OF TERMS

°C	degrees Centigrade
°F	degrees Fahrenheit
AE	Adverse event
ALT	Alanine aminotransferase
ANC	Absolute neutrophil count
AML	Acute myeloid leukemia
aPTT	Activated partial thromboplastin time (also PTT)
ASaT	All Subjects as Treated
ASCT	Autologous Stem Cell Transplant
AST	Aspartate aminotransferase
ASO-PCR	Allele-Specific Oligonucleotide Polymerase Chain Reaction
bid	Twice daily
BSA	Body surface area
BUN	Blood urea nitrogen
CBC	Complete blood count
CFR	Code of Federal Regulations
CHF	Congestive heart failure
CK	Creatinine kinase
CNS	Central nervous system
CR	Complete response
CrCl	Creatinine Clearance
CRF	Case report form(s)
CRM1	Chromosome region maintenance protein 1
CRO	Clinical research organization
CSR	Clinical Study Report
CTCAE	Common Terminology Criteria for Adverse Events
CT Scan	Computed Tomography Scan
CV	Curriculum vitae
CYP450	Cytochrome P450
dL	Deciliter
DLT	Dose-limiting toxicity
DNA	Deoxyribonucleic acid
DOR	Duration of response
DVT	Deep venous thrombosis
EC	Ethical Committee
ECG	Electrocardiogram
ECOG	Eastern Cooperative Oncology Group
EOT	End of Treatment
FAS	Full Analysis Set
FCBP	Females of childbearing potential
FDA	Food and Drug Administration
FISH	Fluorescent in situ hybridization
FLC	Free light chain
FPI	First Patient In
FU	Follow-up
G-CSF	Granulocyte colony stimulating factor

GCP	Good Clinical Practice
GI	Gastrointestinal
GLP	Good Laboratory Practice
GM-CSF	Granulocyte macrophage colony stimulating factor
GSH	Glutathione
h	Hour(s)
HIPAA	Health Insurance Portability and Accountability Act
HIV	Human immunodeficiency virus
HL	Hodgkin's Lymphoma
IA	Interim analysis
IB	Investigator Brochure
ICF	Informed Consent Form
ICH	International Conference on Harmonisation
IEC	Independent Ethics Committee
Igs	Immunoglobulins
IMWG	International Myeloma Working Group
IMiD®	Immunomodulatory
IND	Investigational New Drug (Application)
INR	International Normalized Ratio
IRB	Institutional Review Board
IV	Intravenous
kg	Kilogram(s)
KRAS	Kirsten Rat sarcoma
KRd (CRd)	Kyprolis (Carfilzomib), Revlimid, Dexamethasone
LDH	Lactate dehydrogenase
MFC	Multiparameter Flow Cytometry
mg	Milligram(s)
min	Minute(s)
mIU	Milli International Units
mL	Milliliter(s)
MM	Multiple myeloma
mm^2	Millimeter(s) squared
mm^3	Millimeter cubed
MR	Minimal response
MRD	Minimal Residual Disease
MRI	Magnetic Resonance Imaging
MTD	Maximum tolerated dose
NCI	National Cancer Institute
nCR	Near Complete Response
NCT	National Center for Tumor Diseases
NHL	Non-Hodgkin's lymphoma
NYHA	New York heart association
ORR	Overall response rate
OS	Overall survival
PBMC	Peripheral blood mononuclear cells
PBSCT	Peripheral Blood Stem Cell Transplant
PCCC	Personalized Cancer Care Consortium (of the University of Chicago)
PD	Progressive disease
PDn	Pharmacodynamics

PFS	Progression-free survival
PI	Proteasome Inhibitor
PIS	Patient Information Sheet
PK	Pharmacokinetics
PO	Per os (oral)
PR	Partial response
PSA	Prostate-specific antigen
PT	Prothrombin time
Pt	Patient
PTT	Partial thromboplastin time
QDx5	Daily dosing for five days
QIU	Qualified Investigator Undertaking Form
RA	Regulatory Authority
RBC	Red blood cell
RECIST	Response Evaluation Criteria in Solid Tumors
SAE	Serious adverse event
SAP	Statistical Analysis Plan
sCR	Stringent complete response
SD	Stable disease
SEER	Surveillance, Epidemiology, and End Results
SFLCs	Serum Free Light Chains
SINE	Selective Inhibitor of Nuclear Export
SOC	System Organ Class
SPEP	Serum protein electrophoresis
SPM	Second Primary Malignancies
STD_{10}	Severely toxic dose in 10% of animals
SUSAR	Suspected unexpected serious adverse reaction
TK	Toxicokinetics
TLS	Tumor lysis syndrome
TSH	Thyroid Stimulating Hormone
TSP	Tumor Suppressor Protein
TTP	Time to tumor progression
UCM	University of Chicago Medicine
UPEP	Urine Protein Electrophoresis
URPLWMiPB	The Office for Registration of Medicinal Products, Medical Devices and Biocidal Products
VGPR	Very Good Partial Response

1. INTRODUCTION

1.1 Overview of Multiple Myeloma

Multiple myeloma is a clonal neoplastic proliferation of plasma cells affecting 19,900 US patients each year (Ries LAG, 2007). Multiple myeloma is characterized by anemia, bone destruction, monoclonal gammopathy, renal failure, hypercalcemia and increased susceptibility to infections. The disease is systemic, and chemotherapy is indicated for management of symptomatic myeloma. Current front-line treatments include combination chemotherapy with regimens using melphalan (Alkeran®), bortezomib (Velcade®), thalidomide (Thalomid®), and lenalidomide (Revlimid®) and their combinations with and without corticosteroids. In addition, two agents pomalidomide (Pomalyst®) and carfilzomib (Kyprolis®) have been recently approved in the treatment of relapsed disease. Younger patients are consolidated with high-dose therapy (ablative chemotherapy or radiation) with autologous stem cell transplantation (ASCT). Although improvements in progression-free survival and overall survival have occurred in the past 5 years, even with the best available approved agents, 10-30% of patients fail to respond to the primary therapy, and almost all subjects eventually relapse, with a median overall survival of 44.8 months (Kumar et al., 2008).

1.2 Proteasome Background

The proteasome is a multicatalytic proteinase complex that is responsible for degradation of a wide variety of protein substrates within normal and transformed cells. Intracellular proteins targeted for degradation by the proteasome are first ubiquitinated via the ubiquitin conjugation system. Ubiquitinated proteins are cleaved within the proteasome by one or more of three separate threonine protease activities: a chymotrypsin-like activity, a trypsin-like activity, and a caspase-like activity.

1.3 Carfilzomib Background

Carfilzomib (PR-171) is a tetrapeptide keto-epoxide-based inhibitor specific for the chymotrypsin-like active site of the 20S proteasome. Carfilzomib is structurally and mechanistically distinct from the dipeptide boronic acid proteasome inhibitor bortezomib (Velcade®). In addition, when measured against a broad panel of proteases including metallo, aspartyl, and serine proteases, carfilzomib demonstrated less reactivity against non-proteasomal proteases when compared to bortezomib (Arastu-Kapur S, Nov 2008; Demo et al., 2007).

1.3.1 Carfilzomib Toxicology Studies

In the initial Good Laboratory Practice (GLP)-compliant toxicity studies done by the drug maker, Amgen, carfilzomib was administered to rats and monkeys as two complete two-week cycles of QDx5 for five days with nine days rest (Kirk CJ, Nov 2008). Administration to rats at 12 mg/m^2, the severely toxic dose in 10% of animals (STD_{10}), caused > 90% proteasome inhibition in red blood cells one hour after dosing. Overall, stronger inhibition of the proteasome and longer duration of inhibition was tolerated with carfilzomib compared with bortezomib. Daily administration of bortezomib at anti-tumor doses is not tolerated in animals, and therefore daily bortezomib has not been given in the clinic. A dose-dependent decrease in proteasome activity was demonstrated in animals, and equivalent levels of proteasome inhibition were achieved with administration of carfilzomib as either an intravenous (IV) push or an IV infusion. The dose-limiting toxicities (DLTs) of carfilzomib in both the rat and monkey 28-day GLP toxicity studies included toxicity to the gastrointestinal tract, bone marrow, pulmonary, and cardiovascular systems. No behavioral or histopathological signs of neurotoxicity were observed, and carfilzomib does not cross the blood-brain barrier.

In 6-month rat and 9-month chronic toxicity studies, carfilzomib was administered on Days 1, 2, 8, 9, 15, and 16 of a 28-day cycle, mimicking the active anti-tumor regimen being used in ongoing phase II studies in myeloma and solid tumors (Kirk CJ, Nov 2008). Tolerability was excellent, with no evidence of peripheral (or central) neurotoxicity, including neuropathology, observed, even at high doses. This is in stark contrast to that observed with bortezomib (Bross et al., 2004; FDA, 2003). DLTs included effects on the gastrointestinal, renal, pulmonary, and cardiovascular systems and appeared related to C_{max} effects. Of note, neutropenia was not observed; rather, transient neutrophilia was seen following acute dosing. Renal, cardiovascular and gastrointestinal toxicities were similar to those observed with bortezomib. Finally, cyclical thrombocytopenia, likely due to inhibition of platelet budding from megakaryocytes, was similar to that seen with bortezomib. Proteasome inhibition in the blood in excess of 90% was achievable at well-tolerated doses, which contrasts with the ~70% proteasome inhibition achievable with bortezomib at its maximum tolerated dose (MTD). In summary, these animal toxicity studies support the tolerability of carfilzomib in clinical studies, even on intensive dosing schedules and at doses achieving proteasome inhibition in excess of what can be achieved with bortezomib at its MTD on a less intensive schedule.

1.3.2 Carfilzomib Preclinical Antitumor Activity

Based upon the results of *in vitro* and *in vivo* studies, it is anticipated that the more intense and longer duration of proteasome inhibition that can be achieved with carfilzomib will result in enhanced anti-tumor activity relative to bortezomib. Continuous (72 hr) exposure to carfilzomib is associated with potent cytotoxic and pro-apoptotic activity across a broad panel of tumor-derived cell lines in culture (Demo et al., 2007; Kuhn et al., 2007; Ries LAG, 2007). Incubation of hematologic tumor cell lines with carfilzomib for as little as one hour leads to rapid inhibition of proteasome activity followed by accumulation of polyubiquitinated proteins and induction of apoptotic cell death. Carfilzomib has also been demonstrated to be cytotoxic in bortezomib-resistant tumor cell lines (Demo et al., 2007; Kuhn et al., 2007).

The anti-tumor efficacy of carfilzomib has been tested in immunocompromised mice implanted with a variety of tumor cell lines. In a human colorectal adenocarcinoma model HT-29, administration of carfilzomib on a twice-weekly Day 1, Day 2 schedule resulted in significant reduction in tumor size and was superior to a twice-weekly Day 1, Day 4 schedule using the same dose of carfilzomib, and a once-weekly dosing schedule using twice the dose level. Bortezomib at its MTD has no activity in this xenograft model using the standard Day 1, Day 4 schedule (Demo et al., 2007).

1.3.3 Phase 1 Experience with Carfilzomib as a Monotherapy

A phase 1 clinical trial, PX-171-002, testing carfilzomib in subjects with relapsed/refractory hematologic malignancies, was completed (Alsina M, 2007). During the dose escalation portion of the trial, 36 subjects received carfilzomib on Days 1, 2, 8, 9, 15, and 16 of a 28-day cycle. Subjects with multiple myeloma (MM), non-Hodgkin's lymphoma (NHL), Waldenström's macroglobulinemia, and Hodgkin's Lymphoma (HL) were enrolled on the study.

No DLTs were observed in the initial seven cohorts (doses ranged from 1.2 to 15 mg/m^2) of three subjects each. At the 20 mg/m^2 dose level, one of eight patients had a Grade 3 renal failure at Cycle 1, Day 2 which was considered possibly related to study drug and lasted for six days. The patient continued on study for the remainder of Cycle 1 before having disease progression. At the 27 mg/m^2 dose level, one of six subjects experienced a DLT during Cycle 1, consisting of severe hypoxia with pulmonary infiltrates following Day 2 of dosing. In subjects where the 27 mg/m^2 dose was efficacious, a "first dose effect" was seen that included a constellation of findings that appeared to be the clinical sequelae of rapid tumor lysis syndrome (TLS) and/or cytokine release. This effect was notable for fever, chills, and/or rigors occurring

during the evening following the first day of infusion. On the second day, three of five subjects with multiple myeloma experienced an increase in creatinine to Grade 2 (including the subject with the DLT). This elevation was rapidly reversible and all three subjects were rechallenged with carfilzomib without recurrence of the events. Interestingly, all three subjects had a rapid decline in serum and/or urine M-protein levels; two subjects achieved a partial response (PR) and the third subject achieved a minimal response (MR). There were no consistent changes in potassium, calcium, phosphorous, or uric acid levels although some increases in LDH and other markers of tumor lysis were noted. Because of the possible tumor lysis syndrome (TLS) and reversible creatinine elevations, hydration and very-low dose dexamethasone prophylaxis were instituted in subsequent studies and have essentially eliminated clinically significant TLS/creatinine elevations and the other "first-dose" effects.

Hematologic toxicities were primarily mild or moderate. The thrombocytopenia reported with carfilzomib is cyclical and similar to that reported with bortezomib. The cause and kinetics of the thrombocytopenia following treatment are different from those of standard cytotoxic agents. To maximize the likely benefit of carfilzomib, subjects with thrombocytopenia should be supported as clinically indicated rather than having treatment reduced due to thrombocytopenia.

Of the 36 evaluable patients enrolled in PX-171-002, 20 had MM (Alsina M, 2007). Four MM patients achieved a PR, one of two at the 15 mg/m^2 dose, one of six at the 20 mg/m^2 dose, and two of five at the 27 mg/m^2 dose. The responses have been rapid in onset, beginning in some subjects after 1-2 doses. The duration of response (DOR) ranged from 134 to 392 days. The minimal effective dose was 15 mg/m^2 wherein >80% proteasome inhibition in peripheral blood and mononuclear cells was observed one hour after dosing. The median number of prior therapies for subjects on this trial was five, and responses were seen in subjects who had relapsed from (including some refractory to) bortezomib and/or immunomodulatory agents. Stable disease also occurred in four NHL and five MM subjects, with subjects on therapy for up to 409 days. Such prolonged therapy, at "full" twice-weekly doses, is not possible with bortezomib. These results led to the initiation of two phase 2 studies.

1.3.4 Phase 2 Experience with Carfilzomib as a Monotherapy

Two phase 2 clinical studies were conducted with carfilzomib in MM patients, PX-171-003-A0 (N=46) in relapsed and refractory MM and PX-171-004 (N=39) in relapsed MM. In both studies, patients were dosed with 20 mg/m^2 on Days 1, 2, 8, 9, 15, and 16 on a 28 day schedule. In these studies there were four

cases of suspected or documented TLS prior to institution of the prophylaxis guidelines. Since these guidelines were implemented, no further cases of TLS have been reported including in >350 additional patients with relapsed or refractory MM treated in ongoing phase II studies. In both studies, the most common adverse events were fatigue, anemia, thrombocytopenia (primarily cyclical), gastrointestinal, and dyspnea. Almost all were Grades 1 or 2. There were reported cases of increased in serum creatinine that were primarily < Grade 2 and were transient, rapidly reversible, and non-cumulative. A very low rate of treatment-emergent peripheral neuropathy, 2.2% Grade 3/4, was observed in PX-171-003-A0 despite the fact that 78% of patients had Grade 1/2 neuropathy upon study entry[8].

The response rate in PX-171-003-A0 was 17% PR, 7% MR and 41% SD in these patients that entered the study with progressive disease and were refractory to their most recent therapy, often including bortezomib and/or an immunomodulatory drug (usually lenalidomide). The median time to progression on the PX-171-003-A0 study was 3.5 months with a DOR of 7.2 months (mean follow up of 7.6 months) (Jagannath S, 2009).

A "stepped up" dosing schedule, referred to as 20/27 mg/m^2, has subsequently been incorporated into the PX-171-003 study (referred to as PX-171-003-A1) in order to maximize the clinical benefit of carfilzomib (Siegel et al., 2012). Patients received 20 mg/m^2 for the first cycle and 27 mg/m^2 thereafter. This dosing regimen was overall well tolerated. The most common side effects reported were fatigue (49%), anemia (46%), nausea (45%), and thrombocytopenia (39%). Thirty-three patients (12.4%) experienced peripheral neuropathy, primarily Grades 1 or 2 and were similar to the A0 portion of the study. In addition, anemia rates in the PX-171-003-A1 (higher dose) were lower than those reported in the PX-171-003–A0 portion of the study, possibly indicating that the higher dose of carfilzomib achieves better clearing of neoplastic cells in the bone marrow allowing superior normal marrow reconstitution The overall response rate was 23.7% with median duration of response of 7.8 months. Median overall survival was 15.6 months.

In PX-171-004, a first cohort of patients received 20 mg/m^2. The subset of patients (N=54) that had not seen bortezomib had an overall response rate (ORR) of 46% (2% CR, 9% VGPR and 35% PR), while the bortezomib treated patients (N=33) had an ORR of 18% (3% CR, 3% VGPR and 12% PR) (Siegel D, Nov 2009; Wang L, 2009). The median time to progression (TTP) was 7.6 and 5.3 months in these two groups, respectively. Thus, carfilzomib can induce very high levels of response in patients who have not previously been treated with bortezomib and, even in bortezomib-treated patients, substantial anti-tumor

activity is observed. Of note, disease control (PR + MR + SD) was achieved in ~65% of patients with progressive MM entering the study. Patients on these studies have been treated for >12 cycles with good tolerability and no cumulative toxicity (e.g., bone marrow, severe fatigue, or neuropathy) have been observed.

The protocol was amended to allow patients to increase to 27 mg/m^2 in Cycle 2 or later based on tolerability, similar to that used in PX-171-003 – A1.

Further information about the phase 2 studies is presented in the Investigator's Brochure.

Indications and Usage: Carfilzomib (Kyprolis) was approved by the FDA as a single agent for refractory and relapsed myeloma and in combination with lenalidomide and dexamethasone for the treatment of myeloma patients who have received 1-3 lines of prior therapy. Kyprolis is also approved for use alone to treat relapsed or refractory multiple myeloma. More details can be found in the Package Insert.

1.4 Lenalidomide Background

Lenalidomide (Revlimid) is a proprietary IMiD® compound of Celgene Corporation. IMiD® compounds have both immunomodulatory and anti-angiogenic properties which could confer anti-tumor and anti-metastatic effects. Lenalidomide has been demonstrated to possess anti-angiogenic activity through inhibition of bFGF, VEGF and TNF-alpha induced endothelial cell migration, due at least in part to inhibition of Akt phosphorylation response to bFGF (Dredge et al., 2005). In addition, lenalidomide has a variety of immunomodulatory effects. Lenalidomide stimulates T cell proliferation and the production of IL-2, IL-10 and IFN-gamma, inhibits IL-1 beta and IL-6 and modulates IL-12 production (Dredge et al., 2005). Upregulation of T cell derived IL-2 production is achieved at least in part through increased AP-1 activity (Corral et al., 1999).

Although the exact anti-tumor mechanism of action of lenalidomide is unknown, a number of mechanisms are postulated to be responsible for lenalidomide's activity against multiple myeloma. Lenalidomide has been shown to increase T cell proliferation, which leads to an increase in IL-2 and IFN-gamma secretion. The increased level of these circulating cytokines augment natural killer cell number and function, and enhance natural killer cell activity to yield an increase in multiple myeloma cell lysis (Schafer et al., 2003). In addition, lenalidomide has direct activity against multiple myeloma and induces apoptosis or G1 growth

arrest in multiple myeloma cell lines and in multiple myeloma cells of patients resistant to melphalan, doxorubicin and dexamethasone (Davies et al., 2001).

Indications and Usage: Revlimid (lenalidomide) is indicated for the treatment of patients with transfusion-dependent anemia due to low- or intermediate-1-risk myelodysplastic syndromes associated with a deletion 5q cytogenetic abnormality with or without additional cytogenetic abnormalities. Revlimid is also approved in combination with dexamethasone for the treatment of patients with multiple myeloma that have received at least one prior therapy.

Adverse Events: Most frequently reported adverse events reported during clinical studies with lenalidomide in oncologic and non-oncologic indications, regardless of presumed relationship to study medication include: anemia, neutropenia, thrombocytopenia and pancytopenia, abdominal pain, nausea, vomiting and diarrhea, dehydration, rash, itching, infections, sepsis, pneumonia, UTI, upper respiratory infection, atrial fibrillation, congestive heart failure, myocardial infarction, chest pain, weakness, hypotension, hypercalcemia, hyperglycemia, back pain, bone pain, generalized pain, dizziness, mental status changes, syncope, renal failure, dyspnea, pleural effusion, pulmonary embolism, deep vein thrombosis, CVA, convulsions, dizziness, spinal cord compression, syncope, disease progression, death (not specified) and fractures.

Complete and updated adverse events are available in the Investigator Brochure and the IND Safety Letters.

1.5 Combination Of Proteasome Inhibitors And Immunomodulatory Agents

Proteasome inhibitors (bortezomib, carfilzomib) and immunomodulatory agents (thalidomide and lenalidomide) are both highly effective agents in multiple myeloma. Lenalidomide is an immunomodulatory derivative of thalidomide and has both immunomodulatory and anti-angiogenic properties, which are considered to confer anti-tumor effects. Two pivotal randomized phase 3 trials established that lenalidomide in combination with high-dose dexamethasone produced a significant improvement in overall response rate and time to tumor progression vs. high-dose dexamethasone alone in relapsed multiple myeloma patients with up to 3 prior therapies (Dimopoulos et al., 2007; Weber et al., 2007).

Preclinical studies show that lenalidomide sensitizes multiple myeloma to the proteasome inhibitor bortezomib, suggesting combination therapy may enhance clinical activity. The combination of a proteasome inhibitor and immunomodulatory agent is attractive, as the expected overlapping toxicities would be manageable. A phase 1 dose-escalation study was conducted to determine the MTD and activity of the bortezomib, lenalidomide, and dexamethasone combination in subjects with heavily pre-treated relapsed and/or refractory multiple myeloma (Anderson KC, 2008). The MTD was established as lenalidomide 15 mg and bortezomib 1.0 mg/m^2 with 20 to 40 mg dexamethasone. In 36 evaluable subjects, the overall response rate (CR+PR+MR) was 58%, including 6% CR. Although the regimen is active, the requirement for dose reductions of both agents to achieve a tolerable combination may have resulted in suboptimal complete response rates.

A phase 2 study followed to evaluate the efficacy and safety of lenalidomide, bortezomib, dexamethasone (RVD) at the phase 1 MTD (Richardson P, Dec 6, 2008) found that the overall response rate (CR/nCR+VGPR+ PR+ MR) in 63 response-evaluable patients is currently 86%, including 24% CR/nCR and 67% CR/nCR/VGPR/PR. Response rates according to baseline cytogenetics, disease stage, and prior therapies showed no significant differences according to adverse risk. Toxicities were manageable, consisting primarily of Grade (G) 1-2 myelosuppression. Attributable non-hematologic toxicities included deep vein thrombosis (two patients; attributed to lenalidomide), and two episodes of atrial fibrillation (G3) prompting dexamethasone dose reduction. G3 polyneuropathy was reported in one patient (pt) attributed to bortezomib and leading to treatment discontinuation despite bortezomib dose reduction. Dose reductions were required for: lenalidomide (13 pts); bortezomib (9 pts) and dexamethasone (26 pts).

Lenalidomide and high-dose dexamethasone without a proteasome inhibitor has shown significant activity in untreated disease. In a phase 2 study of 34 subjects with newly diagnosed myeloma, subjects received lenalidomide (25 mg Days 1 to 21 of a 28-day cycle) and high-dose dexamethasone (40 mg Days 1 to 4, 9 to 12, and 17-20). The objective response rate was 91%, with 6% CR, 32% near CR plus VGPR, and 53% PR (Rajkumar et al., 2005). However, when this regimen was compared with a more conventional delivery of dexamethasone (40 mg Days 1, 8, 15, and 22) in a 445-subject study, the more intensive dexamethasone schedule was associated with significantly shorter overall survival relative to the less intensive regimen (1-year survival rates 86% vs. 96.5%, respectively) (Rajkumar P, 2007). The dexamethasone-intensive regimen was associated with higher incidences of thromboembolism,

hyperglycemia, and higher incidences of Grades 3 and 4 toxicities overall. Clearly, better and safer combination regimens for newly diagnosed disease are warranted.

A recently completed phase 1/2 study of bortezomib in combination with lenalidomide and dexamethasone (RVD) in newly diagnosed subjects with multiple myeloma has yielded promising results (Richardson P, 2007; Richardson et al., 2010). Patients (N=66) that received a median of 10 cycles of combination treatment and achieved 39% and 67% across all dose levels had achieved a CR or VGPR, and at MTD 57% and 74%, respectively. The regimen overall was well tolerated; however, dose reductions due to bortezomib were common and 80% of patients developed peripheral neuropathy.

In summary, the combination of proteasome inhibitors and immunomodulatory agents seem to be very active and well tolerated in patients with relapsed and/or refractory MM, including patients who have received prior lenalidomide, bortezomib, thalidomide, and stem cell transplant (SCT). Further investigations with combination therapy with these agents in newly diagnosed disease are warranted.

1.5.1 Experience with Carfilzomib in Combination with Lenalidomide and Dexamethasone

PX-171-006 is a phase 1b study in patients with relapsed multiple myeloma in which carfilzomib was administered in combination with lenalidomide (Revlimid) and dexamethasone (CRd, renamed to KRd). "Low-dose" dexamethasone 40 mg/day was given on Days 1, 8, 15, and 22 in all cases. Carfilzomib was administered IV on Days 1, 2, 7, 8, 15, and 16; lenalidomide was administered PO on Days 1 through 21. The MTD was not reached in this study and the maximum doses used per protocol were: carfilzomib (27 mg/m^2), lenalidomide 25 mg and low-dose dexamethasone (Niesvizky, April 15, 2013). Eight patients were treated in the phase I portion of the trial and 44 patients were accrued to the expansion cohort. After a median follow-up of 24.4 months, the ORR was 76.9% with a median DOR 22.1 months and median progression free survival (PFS) of 15.4 months. The ORR was 69.2% in bortezomib-refractory patients and 69.6% in lenalidomide-refractory patients. Overall the regimen was well tolerated with a median of 9.5 cycles administered. Generally, KRd was well tolerated with manageable adverse events (AEs). The most common Grade 3/4 AEs were hematologic in nature and included Grade 3/4 AEs lymphopenia (48.1%), neutropenia (32.7%), thrombocytopenia (19.2%), and anemia (19.2%). Non-hematologic AEs were generally Grade 1/2 in severity. Dose reductions of carfilzomib due to AEs were required in 7.7% of patients with a discontinuation rate of 19% and not associated with a specific type of AE. In addition, the rate of peripheral neuropathy was similar to that reported for single-agent carfilzomib and did not

increase with the addition of lenalidomide (Wang, Oct 31, 2013). Phase III study (PX-171-009; ASPIRE trial) comparing KRd and Rd (Revlimid/dexamethasone) showed significant improvement in the KRd arm across all categories, including an improvement of primary endpoint of PFS (26.3 vs 17.6 months) with no to significant added toxicity (Stewart et al., 2015).

Together, these results suggest that carfilzomib, lenalidomide, and low-dose dexamethasone in combination are active and well tolerated and that there are no significant overlapping toxicities (in the dose ranges tested). Importantly, lenalidomide-associated neutropenia and thrombocytopenia do not appear to be exacerbated by concurrent treatment with carfilzomib, even up to 27mg/m^2, suggesting that carfilzomib will combine well with other anti-cancer agents.

1.5.2 Dose Rationale

1.5.2.1 Carfilzomib Dose Rationale

Data suggest that carfilzomib as a single agent can produce substantial response rates in myeloma subjects across a variety of dosing cohorts. Responses were seen over a wide therapeutic window, from 15 to 27 mg/m^2. Maximum proteasome inhibition was seen at doses 11 mg/m^2 and higher in whole blood samples taken 1 hour after the first dose. Carfilzomib is rapidly cleared from plasma with an elimination half-life of < 60 minutes at the 20 mg/m^2 dose. Large, single-arm studies of the 27 mg/m^2 dose have demonstrated that this dose is very well tolerated with patients being treated for >10 cycles without cumulative toxicities.

In addition, in multiple preclinical studies, the tolerability of carfilzomib in rats has been shown to be significantly higher when administered as a 30-minute infusion as compared to a rapid IV bolus. Toxicities observed with IV bolus injection of carfilzomib *above the MTD* at a dose of 48 mg/m^2 include evidence of prerenal azotemia (transient increases in BUN > creatinine) as well as lethargy, piloerection, dyspnea, and gastrointestinal bleeding. Notably, death occurred in ~50% of animals at 48 mg/m^2 when carfilzomib was given as a bolus. Administration of the same dose (48 mg/m^2) as a 30-minute continuous infusion was well tolerated, with no changes in BUN and creatinine and substantially reduced signs of lethargy, piloerection, or dyspnea. Moreover, all animals in the infusion treatment groups survived. The only toxicity observed following infusion of carfilzomib for 30 minutes was gastrointestinal bleeding. The reduced toxicity seen with dosing by infusion may reflect the reduced C_{max} of carfilzomib

vs. that with bolus dosing. Inhibition of the pharmacological target of carfilzomib (the chymotrypsin-like activity of the proteasome) was equivalent in the bolus and infusion treatment groups.

In the clinic, the MTD of carfilzomib has not been reached in the multiple myeloma setting, particularly when administered as a 30-minute infusion. 27mg/m^2 of carfilzomib (bolus administration over 2-10 minutes) is well tolerated in MM patients overall and can be tolerated for >12 cycles in late-stage MM patients with substantial comorbidities.

In the phase 1 dose escalation study (PX-171-007) of single-agent carfilzomib, solid tumor patients started in the initial phase 2 portion of the study at 36 mg/m^2 (bolus administration over 2-10 minutes). A review of the tolerability of 36 mg/m^2 carfilzomib in these patients indicates that this regimen was very well tolerated and an overall adverse event profile similar to that seen with the 27 mg/m^2 carfilzomib experience with bolus dosing. Three patients completed >12 cycles of therapy at 36 mg/m^2 with no evidence of cumulative toxicity. There were no significant DLTs observed; the majority of discontinuations on the study were due to progressive disease. Because of the long-term tolerability of carfilzomib, the phase 1b portion of this study was reopened, and a separate arm for multiple myeloma was added.

More recently in the PX-171-007 trial, patients have been treated with carfilzomib given as a 30-minute infusion in order to potentially minimize C_{max}-related infusion events. The protocol was amended and doses of 20/36 (20 mg/m^2 given on Days 1 and 2 of cycle 1 only; followed by 36 mg/m2 for all subsequent doses), 20/45, 20/56 mg/m^2 and so forth are being investigated. Doses of 20/56 mg/m^2 are currently being given in two separate cohorts of patients with advanced MM and advanced solid tumors; the lower doses were well tolerated. Preliminary tolerability information at this dose level (20/56 mg/m^2) indicated that it is reasonably well tolerated, with minimal infusion reactions. In some cases at 20/56 mg/m^2, dexamethasone was increased from 4 mg/dose to 8 mg with the 56 mg/m2 doses in order to reduce fevers and hypotension. Patients with advanced, refractory MM being treated at 36mg/m^2 and 45mg/m^2 have shown very good tolerability (>6 months in some cases) with documented minimal and partial responses in these heavily pretreated patients. These data indicate that carfilzomib 30-minute infusion can be given at very high levels, with >95% inhibition of blood proteasome levels achievable and with (at least) acute tolerability. All protocols using ≥36mg/m^2 carfilzomib are now administering the drug as a 30-minute infusion.

In addition to the above observations, a phase I study of carfilzomib in patients with relapsed and refractory multiple myeloma was reported in abstract form at the 2009 American Society of Hematology meeting which demonstrated that carfilzomib can be safely administered to patients with substantial renal impairment (CrCl < 30, including patients on dialysis) without dose adjustment.[12] These data indicate that carfilzomib does not exacerbate underlying renal dysfunction, and confirm the "pre-renal" etiology of the BUN/creatinine elevations observed with IV bolus carfilzomib.

1.6 Daratumumab Background

Daratumumab is a human immunoglobulin G1 kappa (IgG1ba monoclonal antibody (mAb) that binds with high affinity to a unique epitope on CD38. It is a targeted immunotherapy that binds to tumor cells that overexpress CD38, a transmembrane glycoprotein. Plasma cells from patients with multiple myeloma express high levels of CD38. This target is distinct from those of other approved agents for multiple myeloma therapy. Daratumumab is an FDA approved treatment for multiple myeloma in combination with lenalidomide and dexamethasone. However, daratumumab is not FDA approved when used in combination with lenalidomide, dexamethasone and carfilzomib so this drug combination will be considered experimental.

1.6.1 Daratumumab Toxicology Studies

At the time of the initial protocol development, there were 2 ongoing clinical studies of daratumumab for which preliminary data are available (Study GEN501 and GEN503). Study GEN501 is an open-label, Phase 1/2, first-in-human, single-agent study in subjects with multiple myeloma whose disease is relapsed or refractory to at least 2 prior lines of therapies. Enrollment in Part 1, a dose-escalation ("3+3") design to evaluate daratumumab at doses ranging from 0.005 mg/kg to 24 mg/kg, is complete. Part 2 is a dose expansion design to further examine daratumumab at doses and schedules based on the findings from Part 1. At the time of this protocol development, 26 subjects have been treated in Part 2. Data from Part 1 of Study GEN501 were presented at the American Society of Clinical Oncology (ASCO) Annual Conference in June 2013 (Lokhorst 2013). Among the 32 subjects treated in Part 1, the median number of prior treatment lines was 6. Pharmacokinetic analysis showed plasma peak levels as expected but relatively rapid clearance at low dose levels. At ≥4 mg/kg doses, observed pharmacokinetic values approximated

those predicted by a target-mediated clearance model. Among subjects treated at doses ≥4mg/kg (n=12), 5 partial responses (PRs) and 3 minimal responses (MRs) were observed. No immunogenicity to daratumumab was detected. In Part 1, the most commonly reported adverse events were infusion-related and occurred predominantly during the first full infusion. Across all dose groups, 44% of subjects had infusion-related reactions (IRRs) with a toxicity Grade 1 to 3; 2 events were Grade 3. Six serious adverse events considered to be related to treatment with daratumumab (1 anemia, 1 thrombocytopenia, 2 bronchospasm, 1 cytokine release, 1 aspartate aminotransferase [AST] increase) were reported. Study GEN503 is an open-label, Phase 1/2 multicenter, dose-escalating study investigating the safety of daratumumab in combination with lenalidomide and dexamethasone in subjects with relapsed or refractory multiple myeloma. At the time of this protocol development, 9 subjects have been treated in GEN503: 3 subjects each with 2 mg/kg, 4 mg/kg, and 8 mg/kg daratumumab in combination with the approved doses of lenalidomide (25 mg daily) and dexamethasone (40 mg weekly). No dose-limiting toxicity (DLT) has been observed in the 6 subjects treated in the first 2 cohorts of 2 and 4 mg/kg daratumumab. For the most comprehensive nonclinical and clinical information regarding daratumumab, refer to the latest version of the Investigator's Brochure for daratumumab.

1.6.2 Daratumumab Preclinical Antitumor Activity

Based on preclinical data, daratumumab may utilize multiple effector cell functions, resulting in immune mediated killing of tumor cells. In ex vivo experiments utilizing human bone marrow stromal cells co-cultured with primary multiple myeloma cells, complement-dependent cytotoxicity (CDC) occurs rapidly and demonstrates maximal myeloma cell killing by daratumumab within 1 hour of antibody-mediated activation of the complement proteins (de Wears 2011[9]). Daratumumab-induced antibody-dependent cell-mediated cytotoxicity (ADCC) is slower in its action, with maximal ADCC by daratumumab observed at 4 hours in vitro (de Weers 2011[9]). Daratumumab has also been shown to induce antibody-dependent cellular phagocytosis (ADCP) in the presence of macrophages within 4 hours in vitro (Overdijk 2013[26]). The precise role of some or all of these effector functions in reducing tumor burden in patients is unknown.

1.7 Study Rationale

This study will evaluate using the combination of Daratumumab (Darzalex) with carfilzomib (Kyprolis), lenalidomide (Revlimid), and dexamethasone (D-KRd) as initial treatment for subjects diagnosed with

MM. The proposed strategy is based on the following rationale: (1) The results indicate that the KRd combination (formerly CRd) is highly active in newly diagnosed multiple myeloma (NDMM). In the initial study of KRd treatment of NDMM patients of all ages with protocol-recommended delayed transplant for transplant candidates, best response for all patients on ITT (N=53) was 98% PR, 87% VGPR, 72% >nCR, 64% CR, 55% sCR, and 51% MRD-negative rate, 4-year PFS of 69%.[1] In a more recent and ongoing study of the treatment of transplant candidates with NDMM with KRd pre- and post-autologous stem cell transplant (KRd+ASCT), candidates with 4 KRd cycles before ASCT followed by 4 cycles of KRd consolidation, followed by 10 cycles of KRd maintenance, an addition of transplant appeared to provide statistically higher rates of sCR at the end of 8 cycles (69% vs 30% for KRd+ASCT vs KRd without ASCT) and overall higher rates of sCR, reaching over 80% at the end of KRd treatment and high rates of MRD-negative disease, which are exceeding 70% by either multi-color flow cytometry (MFC) or by next gene sequencing (NGS) at the end of extended total 18 cycles of KRd treatment[1,2]. The extended treatment overall was well tolerated, with comparable toxicity rates between these two studies, and low rates of discontinuation due to toxicities (<5%), and no treatment-related death, which corroborates with observations of generally acceptable safety profile of KRd vs Rd based on randomized ASPIRE study results in relapsed myeloma.[1,2,3] (2) Combination of daratumumab with lenalidomide and dexamethasone is highly active and well tolerated in relapsed MM based on POLLUX randomized study (D-Rd vs Rd), with response rates among the highest, if not the highest in this patient population (>PR 92.9%, CR rate 43.1%, and MRD-negative rate 22.4%), associated with best-to-date PFS in relapsed myeloma of 85.7% at 12 months, with median not reached at median 13.5 months of follow-up, which performed well in all categories significantly higher than in control Rd arm with PFS superior by hazard ratio 0.37; 95% confidence interval [CI], 0.27 to 0.52; P<0.001 by stratified log-rank test. Toxicities were for most categories not significantly different or only slightly higher in daratumumab arm, mostly related to longer treatment on daratumumab arm, with the exception of higher rates of neutropenia and infusion reaction, although these events did not result in higher rates of discontinuation or death.[4] (3) Similarly, the combination of daratumumab with proteasome inhibitor (bortezomib) and dexamethasone (D-Vd) shows superior activity compared to Vd in randomized CASTOR trial, with PR rate in D-Vd arm 82.9%, and with significantly superior PFS compared to Vd arm (not reached vs 7.2 months; hazard ratio 0.39, 95% confidence interval, 0.28 to 0.53; P<0.001). Other than daratumumab-related infusion reactions, adverse events in daratumumab arm were mostly comparable, except higher rates of neutropenia and thrombocytopenia in daratumumab arm, which did not affect discontinuation rates.[5] While there is no data

in public domain on efficacy and safety of combination of daratumumab with carfilzomib or carfilzomib combinations, initial safety data has been collected from the ongoing study MMY1001, which has completed enrollment into D-KRd cohort of 20 patients with weekly infusion of carfilzomib in NDMM, and is completing safety cohort of D-Kd with bi-weekly carfilzomib dosing. Based on analysis of these data, we hypothesize that an addition of daratumumab to KRd (D-KRd combination) will improve treatment outcome of KRd without ASCT, including the rates of CR/sCR and rate of MRD-negative disease. Considering that the results of KRd+ASCT appear to show, as a proof of principle, that KRd results without ASCT (or with deferred ASCT) can further be improved, we hypothesize that the D-KRd treatment without ASCT (or with deferred ASCT) might achieve at least comparable results to the results observed for KRd+ASCT. Since the achievement of CR and MRD-negative status is associated with improved long-term control of the disease[6,7] and daratumumab (in particular) improves both depth of response and time to event in combination with IMiD and PI, this regimen may further improve time to event and overall treatment outcome in NDMM. In addition, we anticipate that the study will inform whether this regimen warrants further evaluation, as the study design is very similar to study designs of completed frontline KRd regimens in new MM. We propose to use the established doses and schedule of KRd as published frontline KRd trials recommended for phase 2[1] and a dose of daratumumab, selected for CASTOR and POLLUX randomized studies and MMY1001 cohorts.

1.7.1 Study Population and Sample Size Justification

A total of 45 non-transplant candidates and/or transplant candidates who agree to defer transplant will be enrolled. The sample size is selected to allow for a collection of sufficient data for evaluation of sCR and MRD rates at the end of 8 cycles, which assumes that we may not have successful MRD evaluation in up to 25% of patients (primary endpoint), and that up to 15% of transplant candidates may change their mind and want to proceed to transplant, both based on historical experience in our prior KRd studies and based on historical rates of sCR from KRd study without ASCT (or deferred transplant) of 30% at the end of 8 cycles, and preliminary rates of sCR and MRD by NGS at the end of Cycle 8 of 69% and 66%, respectively (both to be updated at ASH 2016).

1.8 Assessment for Response

Response will be determined according to the IMWG response criteria for multiple myeloma (2006). Disease assessment for response will include serum protein electrophoresis (SPEP), urine protein

electrophoresis (UPEP), serum free light chains (SFLC), Hevylite test, and immunoglobulins (Igs). Bone marrow procedures should be completed at the time of suspected complete response. Additionally, for subjects who have a plasmacytoma or disease measured by imaging at baseline, plasmacytoma evaluations and radiographic imaging should be completed as per standard of care and in accordance with IMWG response criteria for multiple myeloma. Patients who achieve SD, PR, or CR will continue therapy until disease progression. Patients with disease progression will discontinue the treatment regimen and be removed from protocol.

2 OBJECTIVES

2.1 Primary Objective

The primary objective of this study is to evaluate the rate of sCR response as well as the rate of MRD-negative disease by NGS at the end of 8 cycles of D-KRd.

2.2 Secondary Objectives

Secondary objectives include characterization of efficacy variables:

- Rate of MRD by next generation gene sequencing (NGS) by clonoSEQ® (Adaptive Biotechnologies) at the end of Cycle 8 and at the end of Cycle 24, and then yearly for as long as no PD up to 2 years.
- Duration of response (DOR), progression free survival (PFS), time to progression (TTP), and overall survival (OS).
- Overall response rate defined as partial response or better (>PR) including the rate of VGPR or better (>VGPR) and near-complete response or better (sCR/CR/nCR) across entire treatment in high-risk and low-risk patients at indicated time points and as best response.
- Safety and tolerability of combination of D-KRd.

2.3 Exploratory Objectives

The exploratory objectives of this study are to conduct GEP, proteomics, RNASeq, and gene sequencing studies on pre-treatment patient samples to evaluate the correlation between treatment outcome and pre-treatment patient profile.

3 STUDY ENDPOINTS

3.1 Primary Endpoints

The evaluation of sCR and MRD-negative rates at the end of 8 cycles will be achieved by measuring the time to sCR response and MRD-negative result from start of treatment as defined by IMWG criteria.

3.2 Secondary Endpoints

Secondary endpoints include the following:

- Determining the correlation between sCR response and MRD status at Cycles 8 and 24 and then yearly for as long as no PD up to 2 years as well as PFS.
- Duration of response (DOR), progression free survival (PFS), time to progression (TTP), and overall survival (OS).
- Safety and tolerability of combination of D-KRD measured by frequencies of adverse events, serious adverse events, and adverse events leading to discontinuation of study treatment.

3.3 Tertiary/Exploratory Endpoints

The exploratory objectives of this study are to conduct GEP, proteomics, RNASeq, and gene sequencing studies on pre-treatment patient samples to evaluate the correlation between treatment outcome and pre-treatment patient profile.

4 INVESTIGATIONAL PLAN

4.1 Overview of Study Design and Dosing Regimen

This study will be a multi-center, open-label, phase 2 study, enrolling subjects with newly diagnosed MM requiring systemic chemotherapy. All dosing schedule is based on 28-day cycles. Subjects will receive treatment until disease progression or unacceptable toxicity (whichever occurs first).

Enrolled subjects will initially receive lenalidomide at 25 mg per dose (see below if previously unable to tolerate 25 mg) on Days 1-21. Carfilzomib will be dosed at 20 mg/m^2 on Days 1 and 2 and 36mg/m^2 thereafter (Days 8,9,15, and16). Alternative escalation will be allowed on Cycle 1 if the treating

investigator considers it necessary as follows: 20 mg/m^2 on Days 1 and 2 escalated to 27 mg/m^2 on Days 8 and 9, and then to 36 mg/m^2 on Days 15 and 16. Daratumumab will be administered by subcutaneous injection, provided as a fixed-dosed of 1800 mg and 30,000 units hyaluronidase per 15 mL (120 mg and 2,000 units/mL) for all cycles. The injection will be given by manual push over 3 to 5 minutes in the abdominal SC tissues in left/right locations, alternating between individual doses. Dexamethasone will be dosed at 40 mg PO per week for subjects < 75 years and 20 mg PO per week for subjects ≥ 75 years during Cycles 1-4. Dose modifications will be mandated based on aggressive schedule of dose modification for toxicities as per specific guidelines. Otherwise, patients will continue at their best tolerated dose of lenalidomide on Days 1-21 and their best tolerated dose of carfilzomib on Days 1, 2, 8, 9, 15, 16 in Cycles 1-4 and Days 1, 2, 15,16 in Cycles 5-8.

All subjects will have monthly clinic visits for disease assessments. Treatment will continue on protocol for up to 24 months or if carfilzomib, lenalidomide and daratumumab are permanently discontinued. If dexamethasone is discontinued due to toxicity, carfilzomib, lenalidomide and daratumumab may be continued if tolerated without dexamethasone. Toxicity will be graded using the National Cancer Institute Common Terminology Criteria for Adverse Events version 4. Patients who complete 24 cycles will then continue single-agent lenalidomide at best tolerated dose (up to 25 mg) in 28-day cycles. This regimen will continue until there is progression of the disease or the toxicities require discontinuation of the drug.

Minimal residual disease analysis will be performed using NGS. MRD will be assessed in all bone marrow samples at the beginning of the study (after Cycles 8 and 24), yearly until progression and at any time a bone marrow biopsy is completed to assess response for suspected CR. If a CR is suspected after Cycle 8, a bone marrow biopsy and MRD testing will be completed at Cycle 12 and/or 18 to confirm. From the time CR is established, MRD analysis should continue at 12-month intervals from randomization up to 5 years in patients with ongoing sCR. The MRD samples will be evaluated by Adaptive Biotechnologies using LymphoSIGHT® platform after isolation of DNA at the University of Chicago site. For this purpose (and to establish a baseline), sites will be required to provide unstained pre-treatment slides to assess MRD status by Adaptive Biotechnologies. MRD analysis will be used only for assessment of treatment response at specified timepoints, and not for decision-making purposes.

Treatment responses will be assessed by serum free light chains (SFLC), Hevylite test, quantitative immunoglobulin levels (Igs), and serum and urine monoclonal protein starting at Cycle 2 Day 1 and at the

beginning of each subsequent cycle. Subjects with stable disease or better will continue treatment until disease progression or the development of unacceptable toxicities. All patients will then undergo a final visit (end of treatment visit).

4.1.1 Number of Centers

Between 4 and 6 study centers will be selected for participation in the study.

4.1.2 Definition of Treatment Cycle and Duration

4.1.2.1 Cycle Duration

Each cycle will be 28 days.

4.1.2.2 Treatment Phase

Treatment will be continued until progression of disease according to IMWG criteria, unacceptable toxicities occur in individual subjects or consent is withdrawn. Cycles 1 through 4 are considered D-KRd induction cycles, Cycle 5-8 D-KRd consolidation, and Cycles 9-24 D-KRd maintenance cycles. After Cycle 4, subjects who are deemed candidates for ASCT will proceed to stem cell harvest as per institutional guidelines. However, the study design recommends a "delayed" transplant for ASCT candidates, as in subject having completed frontline KRd study without or with delayed transplant. This means that patients who are transplant candidates enrolled in this study will not proceed to ASCT; rather, they will have cells frozen down for a potential future transplant at progression or unacceptable toxicity after Cycle 4. After completion of stem cell harvest, patients will resume protocol treatment.

In the event that a subject's risk status is unknown at the time of study entry, the subject will be required to re-consent to continue participation in this study if it is later determined that the subject has high-risk disease status and therefore may benefit from ASCT.

4.1.3 End of Treatment Visit

Patients that discontinue from treatment will undergo an end-of-treatment visit, regardless of the reason of discontinuation, 28 days after the last dose of study medication.

4.1.4 Long Term Follow Up

Patients will be followed up every 3 months for progression and survival from the completion of the safety follow-up visit. Patients who progress during treatment will be followed for survival for 2 years from the completion of the safety follow-up visit or until death, whichever occurs first. The follow up can be done over the phone. For additional details on follow up duration see Section 6.6.1.

5 Patient Selection

5.1 Inclusion Criteria

Subjects must meet all of the following inclusion criteria to be eligible to enroll in this study. No enrollment waivers will be granted.

1. Newly diagnosed, previously untreated myeloma requiring systemic chemotherapy
    - Prior treatment of hypercalcemia or spinal cord compression or active and/or aggressively progressing myeloma with corticosteroids or lenalidomide or bortezomib-based regimens does not disqualify the patient (the treatment dose should not exceed the equivalent of 160 mg of dexamethasone in a 4-week period or not more than 1 cycle of PI/IMiD-based therapy).

2. Both transplant and non-transplant candidates are eligible

3. Diagnosis of symptomatic multiple myeloma as per current IMWG uniform criteria prior to initial treatment

4. Monoclonal plasma cells in the BM ≥ 10% or presence of a biopsy-proven plasmacytoma

5. Measurable disease, prior to initial treatment including any emergent treatment of myeloma. Measurable disease will be indicated by one or more of the following:

    - Serum M-protein ≥ 1 g/dL
    - Urine M-protein ≥ 200 mg/24 hours
    - If serum protein electrophoresis is felt to be unreliable for routine M-protein measurement, then quantitative immunoglobulin levels are acceptable

- Serum Freelite measurable disease as per current IMWG criteria

6. Bone marrow specimen will be required at study entry; available DNA sample from pre-induction BM will be used for calibration step for MRD evaluation by gene sequencing

7. Males and females ≥ 18 years of age

8. ECOG performance status of 0-1

9. Adequate hepatic function, with bilirubin ≤ 1.5 x ULN and aspirate aminotransferase (AST) and alanine aminotransferase (ALT) ≤ 3 xULN

10. ANC ≥ 1.0 x 10^9/L, hemoglobin ≥ 8 g/dL, platelet count ≥ 75 x 10^9/L

11. Calculated creatinine clearance (by Cockcroft-Gault) ≥ 50 mL/min or serum creatinine below 2 g/dL

12. FCBP must have 2 negative pregnancy tests (sensitivity of at least 50 mIU/mL) prior to initiating lenalidomide. The first pregnancy test must be performed within 10-14 days before and the second pregnancy test must be performed within 24 hours before lenalidomide is prescribed for Cycle 1 (prescriptions must be filled within 7 days).

13. FCBP must agree to use 2 reliable forms of contraception simultaneously or to practice complete abstinence from heterosexual intercourse during the following time periods related to this study: 1) for at least 28 days before starting lenalidomide; 2) while participating in the study; and 3) for at least 90 days after discontinuation from the study.

14. Male subjects must agree to use 1 highly-effective method of contraception and 1 effective method of contraception at the **same time** during sexual contact with females of childbearing potential while participating in the study and for at least 90 days following discontinuation from the study even if he has undergone a successful vasectomy.

15. All study participants in the US must be consented to and registered into the mandatory Revlimid REMS® program and be willing and able to comply with the requirements of Revlimid REMS®.

16. Voluntary written informed consent

5.2 Exclusion Criteria

Patients meeting any of the following exclusion criteria are not eligible to enroll in this study. No enrollment waivers will be granted.

1. Frail non-transplant candidates, defined as in Palumbo et al, Blood 2015
2. Non-secretory or hyposecretory multiple myeloma, prior to initial treatment defined as <1.0 g/dL M-protein in serum, <200 mg/24 hr urine M-protein, and no measurable disease as per IMWG by Freelite®
3. POEMS syndrome (polyneuropathy, organomegaly, endocrinopathy, monoclonal protein, and skin changes)
4. Amyloidosis
5. Plasma cell leukemia
6. Waldenström's macroglobulinemia or IgM myeloma
7. Radiotherapy to multiple sites or immunotherapy within 4 weeks before start of protocol treatment (localized radiotherapy to a single site at least 1 week before start is permissible)
8. Participation in an investigational therapeutic study within 3 weeks or within 5 drug half-lives (t1/2) prior to first dose, whichever time is greater
9. Patients not able to tolerate daratumumab, carfilzomib, lenalidomide or dexamethasone
10. Peripheral neuropathy ≥ Grade 2 at screening
11. Diarrhea > Grade 1 in the absence of antidiarrheals
12. CNS involvement
13. Pregnant or lactating females
14. Major surgery within 3 weeks prior to first dose

15. Myocardial infarction within 6 months prior to enrollment, NYHA Class III or IV heart failure, uncontrolled angina, severe uncontrolled ventricular arrhythmias, or electrocardiographic evidence of acute ischemia or active conduction system abnormalities

16. Prior or concurrent pulmonary embolism

17. Known moderate or severe persistent asthma or known chronic obstructive pulmonary disease (COPD)

    a. Known or suspected chronic obstructive pulmonary disease (COPD) with a forced expiratory volume in 1 second (FEV1) <50% of predicted normal

    b. Moderate or severe persistent asthma within the past 2 years, or currently has uncontrolled asthma of any classification. Note that subjects who currently have controlled intermittent asthma or controlled mild persistent asthma are allowed in the study.

18. Rate-corrected QT interval of electrocardiograph (QTc) > 470 msec on a 12-lead ECG during screening

19. Uncontrolled hypertension or diabetes

20. Acute infection requiring systemic antibiotics, antivirals, or antifungals within two weeks prior to first dose

21. Known seropositive for or active viral infection with human immunodeficiency virus (HIV) or hepatitis C virus (HCV)

22. Seropositive for hepatitis B (defined by a positive test for hepatitis B surface antigen [HBsAg]). Subjects with resolved infection (ie, subjects who are HBsAg negative but positive for antibodies to hepatitis B core antigen [anti-HBc] and/or antibodies to hepatitis B surface antigen [anti-HBs]) must be screened using real-time polymerase chain reaction (PCR) measurement of hepatitis B virus (HBV) DNA levels. Those who are PCR positive will be excluded. EXCEPTION: Subjects with serologic findings suggestive of HBV vaccination (anti-HBs positivity as the only serologic

marker) AND a known history of prior HBV vaccination, do not need to be tested for HBV DNA by PCR.

23. Non-hematologic malignancy or non-myeloma hematologic malignancy within the past 3 years except a) adequately treated basal cell, squamous cell skin cancer, thyroid cancer, carcinoma in situ of the cervix, or prostate cancer < Gleason Grade 6 with stable prostate-specific antigen levels or cancer considered cured by surgical resection alone.

24. Any clinically significant medical disease or condition that, in the investigator's opinion, may interfere with protocol adherence or a subject's ability to give informed consent.

5.3 Subject Enrollment and Registration

5.3.1 Registration Process

Prior to registration and any study-specific evaluations being performed, all patients must have given written informed consent for the study and must have completed the pre-treatment evaluations. Patients must meet all of the eligibility requirements listed in Section 5.

Registration will happen separately between the sites. All patients who have signed consent, regardless of if they are enrolled or not, will be entered into the University of Chicago Clinical Trials Management database, eVelos, within 24 hours of signing consent. Subjects will be assigned a unique subject number that will remain consistent for the duration of the study. Sites should make all enrollment requests at least 72 hours before the anticipated start date of Cycle 1 Day 1. Screening and on-treatment assessments are expected to be entered into eVelos within two weeks of study required visits.

The Lead PI or designee will be responsible for approving the eligibility of all patients at corresponding sites. Specifically, once all required screening assessments have been performed, the information will have to be reviewed by the Lead Principal Investigator or designee. When the subject's study eligibility has been confirmed by the Lead Principal Investigator or designee, they or authorised site staff, will finalize data entry into eVelos. Treatment may not start until the approved enrollment form is sent back to the site.

When a potential patient has been identified, notify the CRA via phone or email to ensure a reservation on the study: [redacted]. Reservations for potential subjects will only be held for subjects who have signed consent for that particular study.

When registering a subject, the following must occur:

- Confirm that the institution has a current IRB approval letter for the correct version of protocol/consent and has an annual update on file, if appropriate.
- Submit all required materials (eligibility checklist, source documentation, and full copy of the signed informed consent form) to confirm eligibility and required pre-study procedures to the CRA a minimum of 48 hours prior to the subject's scheduled therapy start date.
- Source documentation includes copies of all original documents that support each inclusion/exclusion criteria. The eligibility checklist does not serve as source documentation but rather as a checklist that original source documentation exists for each criterion.
- Communicate with the CRA to ensure all necessary supporting source documents are received and the potential subject is eligible to start treatment on schedule. If there are questions about eligibility, the CRA will discuss it with the Lead PI. Lead PI may clarify, but not overturn, eligibility criteria.
- Affiliate sites must confirm registration of subjects by obtaining a subject study ID number from the CRA via phone, fax or email.
- If a subject does not start on the scheduled Day 1 treatment date, promptly inform the CRA as the delay in start may deem the subject ineligible and/or require further or repeat testing to ensure eligibility.
- The date the patient's eligibility is confirmed by the CRA will be considered the patient's "Enrolled/On Study Date." The patient's subject ID will be assigned and a confirmation of registration will be issued by the Lead Site CRA on this date. Subjects that sign consent and do not go "On Study" will be recorded in the database as "Not Enrolled" with the date they signed consent and the reason they were not enrolled (e.g., Ineligible, Screen Failure or Withdrawn Consent).

6 TREATMENT PLAN

Please refer to the study calendar (Appendix 8) for an overview.

After screening, eligibility determination and enrollment, subjects will receive daratumumab, carfilzomib, lenalidomide, and dexamethasone, in 28-day cycles until progression, unacceptable toxicity or subject withdraws consent. The total study enrollment period is expected to be 12-18 months. A subject is considered to have completed the treatment phase of the study 4 weeks (28 days) after the end of the last treatment cycle. Excluding long-term follow up, the treatment phase of the study will be completed once the last subject completes t the 28-day safety follow-up visit. Subjects who have not progressed will be followed for progression for up to 2 years from the completion of the safety follow-up visit. Subjects who have progressed, or have been removed from treatment for another reason, will be followed for survival for up to 2 years from the completion of the safety follow-up visit. An attempt will also be made to follow ASCT subjects for progression.

A subject is considered to be off-treatment following a 28-day safety follow-up period after the last treatment. Long-term follow-up for survival will be 2 years from the end-of-treatment visit.

6.1 Study Procedures

6.1.1 Screening Procedures

The screening period is 21 days in length. The screening period starts only after the patient has signed the informed consent form. Refer to the Schedule of Events (Appendix 8).

**Signed Written Informed Consent**	Obtained prior to any study-specific assessments
**Demographics and Medical History**	• Age, gender, ethnic background<br>• Details on myeloma diagnosis<br>• Details on prior cancer therapy, including start and stop dates, disease progression during or after therapy, as well as discontinuation due to toxicities

	• Previous and concurrent relevant diseases<br>• Current symptoms and/ or residual toxicities from prior therapies
**Pregnancy Test (if applicable)**	FCBP must have two negative pregnancy tests (sensitivity of at least 50 mIU/mL) prior to starting study treatment. The first pregnancy test must be performed within 10-14 days prior to the start of study treatment and the second pregnancy test must be performed 24 hours before lenalidomide is prescribed.
**Physical Examination and Vital Signs**	• Body height and weight<br>• BSA<br>• ECOG Performance Status (Appendix 2)<br>• Blood pressure, pulse, temperature<br>• Physical examination
**Cardiac evaluation**	12-lead ECG
**Echocardiogram**	
**Urinalysis**	Urine bilirubin, glucose, hemoglobin, ketones, pH, protein
**Hematology (CBC)**	Hemoglobin, hematocrit, white blood cell (WBC) count; WBC differential, red blood cell count, platelets. WBC differential may be automated or manual as per institutional standards.
**Clinical chemistry**	Sodium, potassium, chloride, bicarbonate, BUN, creatinine, glucose, calcium, phosphate, magnesium, ALT, AST, alkaline phosphatase, total bilirubin, total protein, albumin, urate, LDH, CRP
**β2 Microglobulin**	

**Myeloma Disease Assessment Laboratory**	M-protein determination:<br>• Serum protein electrophoresis and immunofixation<br>• Urine protein electrophoresis and immunofixation<br>• Serum free light chains and Hevylite test<br>• Serum quantitative immunoglobulins<br>All of the above assessments are required at screening regardless of the disease classification.
**Bone Marrow Biopsy**	Quantify percent myeloma cell involvement, obtain bone marrow aspirate for MRD analysis by NGS, and obtain bone marrow aspirate for conventional cytogenetics and fluorescent *in situ* hybridization. This is required at screening. For subjects who sign consent for correlative samples, an additional aspirate sample should be collected at screening. Calibration sample for MRD is required
**CT-PET**	A CT-PET must be performed to confirm MRD-negative disease per standard of care, at every time point when MRD is checked
**Skeletal Survey**	May be within 30 days of starting planned treatment (does not need to be repeated if within 30 days). Includes: lateral radiograph of the skull, anteroposterior and lateral views of the spine, and anteroposterior views of the pelvis, ribs, femora, and humeri, whole body MRI, or whole body CT.
**Neurotoxicity Assessment**	Includes Neurotoxicity Questionnaire (Appendix 5)
**QOL Assessment**	Patient reported survey (Appendix 6)
**Adverse Events**	Only SAEs considered related to study procedure need to be reported.

6.1.2 Treatment Phase Procedures

The following assessments should be performed on Day 1 of each cycle before administering drug unless otherwise noted. Please refer to Appendix 4 for details of response evaluation to be completed at any time throughout the trial when a CR or better is suspected.

**Complete Physical Examination and Vital Signs** on Day 1<br>**Symptom-directed Physical Exam** Days 8 and15<br>**Vital Signs** on each treatment day with carfilzomib	• Body weight<br>• BSA<br>• ECOG Performance Status<br>• Blood pressure, pulse, temperature<br>• Pulse oximetry to investigator discretion
**Hematology**<br>Days 1, 8, and15 before carfilzomib administration	Hemoglobin, hematocrit, WBC count; WBC differential, red blood cell count, platelets. WBC differential may be automated or manual as per institutional standards.
**Complete Clinical Chemistry**<br>Days 1, 8, and 15 before carfilzomib administration	Sodium, potassium, chloride, bicarbonate, BUN, creatinine, glucose, uric acid, total protein, albumin, calcium, phosphorus, magnesium, total bilirubin, alkaline phosphatase, ALT, AST, LDH. Results must be reviewed before dosing in Cycles 1 and 2.
**Limited Clinical Chemistry**<br>Days 2, 9, and 16 if clinically indicated	Sodium, chloride, bicarbonate, BUN, creatinine, glucose, uric acid
**Pregnancy Test**	FCBP with regular or no menstrual cycles must agree to have pregnancy tests every 7 days (weekly) for the first 28 days of study participation and then every 28 days while on study. If menstrual cycles are irregular, the pregnancy testing must occur weekly for the first 28 days and then every 14 days while on study.
**Myeloma Disease Assessment Laboratory**	M-protein determination:<br>• Serum protein electrophoresis and immunofixation<br>• Urine protein electrophoresis and immunofixation<br>• Serum free light chains and Hevylite test<br>• Serum quantitative immunoglobulins<br>Only those assessments used to follow the myeloma disease are required past screening. All assessments are required for confirmation of response.
**Aspirate/Biopsy**	All BM aspirates are SOC. Quantify percent myeloma cell involvement, and obtain bone marrow aspirate for MRD analysis by NGS. This is required at end of Cycle 8, 24 and confirmation of CR. If a CR is suspected after Cycle 8, a bone marrow biopsy and MRD testing will be completed at Cycle 12 to confirm. For subjects who sign consent for correlative

	samples, an additional aspirate sample should be collected at the time of the SOC BMA.
**CT-PET**	A CT-PET must be performed to confirm MRD-negative disease per standard of care (SOC), at every timepoint when MRD is checked
**Neurotoxicity Assessment**	Including Neurotoxicity Questionnaire (Appendix 5)
**QOL Assessment**	Patient reported survey (Appendix 6)
**Adverse Events**	Assessed on an ongoing basis
**Study Treatment**	Section 7 and Appendix 8 (Schedule of Events)

6.1.3 End of Treatment Procedures and Long-Term Follow-Up

Patients who discontinue therapy for any reason must have and end of treatment (EOT) visit completed 28 days (± 7 days) after the last application of study drug. Following the end of treatment, subjects will be followed for survival and progression for 2 years.

At the EOT visit, the patients will undergo the following assessments:

**Pregnancy Test (if applicable)**	FCBP pregnancy testing required at treatment discontinuation and at 28 days following treatment discontinuation. If menstrual cycles are irregular, additional testing 14-days following treatment discontinuation is required
**Physical Examination and Vital Signs**	• Body weight<br>• Blood pressure, pulse, temperature<br>• Physical examination<br>• Pulse oximetry to investigator discretion
**Hematology**	Hemoglobin, hematocrit, WBC count, WBC differential, red blood cell count, platelets. WBC differential may be automated or manual as per institutional standards.
**Clinical Chemistry**	Sodium, potassium, chloride, bicarbonate, BUN, creatinine, glucose, uric acid, total protein, albumin, calcium, phosphorus, magnesium, total bilirubin, alkaline phosphatase, ALT, AST, LDH.
**Myeloma Disease Assessment–Laboratory**	M-protein determination:<br>• Serum protein electrophoresis and immunofixation

	• Urine protein electrophoresis and immunofixation<br>• Serum free light chains and Hevylite test<br>• Serum quantitative immunoglobulins (Igs)
**Bone Marrow Aspirate/Biopsy**	All BM aspirates are SOC. Quantify percent myeloma cell involvement, and obtain bone marrow aspirate for MRD analysis by NGS. This is required at end of treatment, yearly after EOT up to 2 years, and confirmation of CR. For subjects who sign consent for correlative samples, an additional aspirate sample should be collected at the time of the SOC BMA
**CT-PET**	A CT-PET must be performed to confirm MRD-negative disease per standard of care, at every time-point when MRD is checked
**Neurotoxicity Assessment**	Including Neurotoxicity Questionnaire (Appendix 5)
**QOL Assessment**	Patient reported survey (Appendix 6)
**Adverse Events**	Record through 30-days after last treatment. All SAEs considered related to treatment must be followed until resolution.

6.2 Pretreatment Preparation

6.2.1 Hydration

IV hydration will be given immediately prior to carfilzomib during Cycle 1. This will consist of 250 mL normal saline or other appropriate IV fluid. If lactate dehydrogenase (LDH) or uric acid is elevated (and/or in subjects considered still at risk for TLS) additional IV hydration can be considered, but with caution to avoid over hydration.

6.2.2 Concomitant Medications

Concomitant medication is defined as any prescription or over-the-counter preparation, including vitamins and supplements.

The following are **required** concomitant medications to be started on Cycle 1 Day 1 or up to 24 hours prior to Cycle 1 Day 1:

- Valacyclovir 500 mg PO QD or equivalent HZV prophylaxis, continuing for the duration of treatment. Additional prophylaxis is at the treating investigator's discretion.

- Patients receiving multi-agent chemotherapy are inherently at greater risk of thrombosis and should follow the anticoagulation recommendations from International Myeloma Working Group guidelines. This includes LMWH 40 mg subcutaneously daily or warfarin target INR 2-3. As an alternative to LMWH or warfarin, we would accept prophylactic doses of direct oral anticoagulants (apixaban 2.5 mg twice daily or rivaroxaban 10 mg daily). If patient is unable to receive any of the previously listed options and has no risk of thrombosis/no prior thrombosis the patient may proceed with aspirin, preferably 325 mg, after discussing with the Lead Study PI. **Patients with platelets < 30,000/mm^3 or risk of platelets dropping to Grade 3 thrombocytopenia or with active bleeding should have all anticoagulation treatment held.**

The following are **recommended** medications to be started on Cycle 1 Day 1 or up to 24 hours prior to Cycle 1 Day 1:

- Lansoprazole (Prevacid®) 15 mg PO QD, or other PO proton-pump inhibitor or H1 blocker to prevent peptic disease for the duration of treatment. Note that this is a recommended (optional) treatment.

- Mycostatin or nystatin to prevent oral thrush. Note that this is a recommended (optional) treatment.

- Allopurinol (or other approved uric acid-lowering agent) in subjects at high risk for tumor lysis syndrome due to high tumor burden may be prescribed at the treating investigator’s discretion. Allopurinol should be prescribed according to the package insert.

-

Recommended Concomitant Therapy for Daratumumab SC Dosing:

All participants will receive the following medications 1 to 3 hours prior to each study drug administration:

- An antipyretic: Acetaminophen 650 mg PO
- An antihistamine: diphenhydramine 25-50 mg IV or PO or equivalent. Avoid IV use of promethazine. After Cycle 6, if a participant has not developed an infusion-related reaction and is intolerant to antihistamines, modifications are acceptable as per investigator discretion.

- Corticosteroids as indicated in 6.3.3.
- Montelukast 10 mg PO

- For patients considered high-risk for an infusion reaction because of underlying pulmonary conditions, inhaled or nebulized albuterol along with inhaled combined long-acting beta agonist + corticosteroid can be considered at the discretion of the investigator.

6.2.2.1 Contraception

Females of childbearing potential must:

- Avoid pregnancy for at least 4 weeks before beginning lenalidomide.

- Have 2 negative pregnancy tests prior to starting treatment, the first test within 10 to 14 days and the second test 24 hours before prescribing lenalidomide.

- Take pregnancy tests weekly for the first 28 days of treatment.

- Take pregnancy test every 28 days during treatment after the first month. For women of childbearing potential with irregular menstruation, pregnancy tests must be taken weekly for the first 28 days of treatment and every 14 days during treatment.

- Agree to abstain from heterosexual sexual intercourse or to use 2 methods of effective contraception beginning 4 weeks prior to initiating treatment with lenalidomide, during therapy, during dose interruptions and for 90 days following the last dose of drug (more frequent pregnancy tests may be conducted if required per local regulations). Carfilzomib is prohibited in breast-feeding women.
- Abstain from donating eggs during the study and for 90 days after last dose of study drug.

A FCBP is defined as a sexually mature female who: 1) has not undergone a hysterectomy or bilateral oophorectomy, or 2) has not been naturally postmenopausal for at least 24 consecutive months (i.e., no menses at any time in the preceding 24 consecutive months). Amenorrhea following cancer therapy does not rule out childbearing potential.

Male subjects and their partners must use 1 highly effective method of birth control plus 1 additional effective method of birth control (contraception) at the **SAME TIME** during treatment and for 90 days following the last dose of drug, even if they have undergone a successful vasectomy. Male subjects must not donate sperm during the study and for 90 days after your last dose of study drug.

Highly effective methods of contraception include:

- Intrauterine device (IUD)
- Hormonal therapy (birth control pills, injections, implants)
- Tubal ligation
- Vasectomy

Additional effective methods include:

- Latex condom
- Diaphragm
- Cervical Cap

6.2.3 Prohibited Concomitant Medications

Concurrent therapy with a marketed or investigational anti-cancer therapeutic is not allowed.

Corticosteroids for non-malignant conditions (e.g., asthma, inflammatory bowel disease) equivalent to a dexamethasone dose ≥ 4 mg/day or prednisone > 20 mg/day are not permitted. Other investigational agents are not to be used during the study.

6.2.4 Use of Blood Products

Subjects may receive red blood cell or platelet transfusions, if clinically indicated, per institutional guidelines. Patients who require repeated transfusion support should be discussed with the Lead Principal Investigator.

Appropriate anti-coagulation is allowed during the study (e.g.: LMW heparin, direct factor Xa inhibitors, etc.). Warfarin is allowed during the study provided that patients are monitored for INR twice a week during the first two cycles of therapy, then weekly to bi-weekly thereafter.

Patients may receive supportive care with bisphosphonates, erythropoietin, darbepoetin, G-CSF or GM-CSF, pegylated growth factors, and platelet stimulatory factors, in accordance with clinical practice or institutional guidelines prior to entry and throughout the study.

6.2.5 Radiation Treatment

If clinically indicated, palliative radiation therapy to non-target lesions is permitted but study drugs should be held for 3-5 days before the start of palliative radiation therapy and 3-5 days after palliative radiation therapy.

6.2.6 Management of Hepatitis B Virus Reactivation

Primary antiviral prophylaxis is permitted as per local standard of care. Per protocol, HBV DNA testing by PCR is mandatory for subjects at risk for HBV reactivation (see Appendix 8).

For subjects who are diagnosed with HBV reactivation while on treatment, study treatment should be interrupted until the infection is adequately controlled. If the benefits outweigh the risks, study treatment

may be resumed with concomitant antiviral prophylaxis as per local standard of care. Consult a liver disease specialist as clinically indicated.

6.3 Study Drug Administration

6.3.1 Carfilzomib Administration

Carfilzomib for injection is supplied as a lyophilized parenteral product in single-use vials. The lyophilized product is reconstituted with water for injection to a final carfilzomib concentration of 2.0 mg/mL prior to administration. The dose will be calculated using the subject's actual BSA at baseline. Subjects with a BSA > 2.2 m^2 will receive a dose based upon a 2.2 m^2 BSA. Dose adjustments do not need to be made for weight gains/losses of ≤ 10%. Subjects with a Body Surface Area (BSA) of greater than 2.2 m^2 will receive a capped dose of 44 mg of carfilzomib (at the 20 mg/m^2 dose level), 59.4 mg of carfilzomib (at the 27 mg/m^2 dose level), or 79.2 (at the 36 mg/m^2 dose level).

Carfilzomib will be given as an IV infusion over 30 minutes on Days 1, 2, 8, 9, 15 and 16 of Cycles 1- 8 and Days 1, 2, 15 and 16 of Cycles 9-24, and must be administered prior to daratumumab. If the subject has a dedicated line for carfilzomib administration, the line must be flushed with a minimum of 20 mL of normal saline prior to and after drug administration. The dose will be administered at a facility capable of managing hypersensitivity reactions. Subjects will be pre-hydrated with 250mL normal saline or other appropriate IV fluid formulation on cycle 1 Day 1. Other hydration procedures will be optional and per the discretion of the treating investigator. Subjects will remain at the clinic under observation for at least 1 hour following each dose of carfilzomib in Cycle 1 and following the dose on Cycle 2, Day 1. Subjects should be monitored periodically during this period for evidence of fluid overload. Serum chemistry values, including creatinine, must be obtained and reviewed prior to each dose of carfilzomib during Cycles 1 and 2. Refer to Table 6-8 for guidance regarding dose reduction in subjects with compromised renal function.

Doses of carfilzomib may be rescheduled up to 2 days if the scheduled day falls upon a holiday or with approval from the Lead Principal Investigator. If Day 2 of Carfilzomib dosing is delayed (i.e., Day 2, 9, 16) 4 mg of dexamethasone premedication is required to be used prior to second treatment. Missed doses will not be replaced during a cycle. Carfilzomib will be escalated from 20 mg/m^2 (Cycle 1 Days 1 and 2)

to 36mg/m^2 (Cycle 1 Days 8, 9, 15, 16 - see below) as long as the subject does not present with cytokine release symptoms (fever, rash, dyspnea, etc.) after Cycle 1 Day 2. If the subject does have concerning symptoms, they will be treated at 27mg/m^2 on Days 8 and 9 before being escalated to 36mg/m^2.

- Cycle 1: 20 mg/m^2 Days 1, 2; 36 mg/m^2 Days 8, 9, 15, 16. Alternatively, intermediate dose escalation (to 27mg/m^2 on Days 8, 9 of Cycle 1) will be allowed at the treating investigator's discretion.

- Cycle 2-8: 36 mg/m^2 (or best tolerated dose) Days 1, 2, 8, 9, 15 and 16

- Cycles 9-24: 36 mg/m^2 (or best tolerated dose) Days 1, 2, 15 and 16

6.3.2 Lenalidomide Administration

US: Lenalidomide will be prescribed and obtained commercially in accordance with the Revlimid REMS® program of Celgene Corporation. Per standard Revlimid REMS® requirements, all investigators who prescribe lenalidomide for research subjects enrolled into this trial must be registered and must comply with all requirements of the Revlimid REMS® program. Prescriptions must be filled within 7 days. Only enough lenalidomide for one cycle of therapy will be supplied to the patient each cycle. Unused lenalidomide will be counted and documented by each site. Unused lenalidomide will be returned to Celgene by the site using instructions provided by Celgene.

Subjects will receive lenalidomide as follows in 28-day cycles:

- Cycles 1-24: 25 mg (or best tolerated dose) PO Days 1-21

Lenalidomide should be taken each evening at approximately the same time. Lenalidomide is taken with water on a full or empty stomach. Subjects should not break, chew or open capsules. If possible, late doses of lenalidomide should be taken on the assigned day but should not be made up the next day. Vomited doses will not be made up. Subjects should be instructed to never take lenalidomide past Day 21 of each cycle.

6.3.3 Dexamethasone Administration

Dexamethasone will be administered between 30 minutes and 4 hours preceding carfilzomib (on days that they coincide), as follows:

Dexamethasone will be administered between 30 minutes and 4 hours preceding carfilzomib and between 1 to 3 hours preceding daratumumab (on days that they coincide), as follows:

- Cycles 1-4: 40 mg PO (subjects < 75 years) or 20 mg PO (subjects ≥ 75 years) per week
- Cycles 5-24: 20 mg PO per week

For weeks of daratumumab injection, dexamethasone dose is split into two doses: half the dose prior to daratumumab infusion and half the dose the day after.

- Split weekly dosing for all other days (e.g. 10 mg on Day 1 and 10 mg on Day 2, etc.) is permitted with approval from Lead Principal Investigator.
- Dexamethasone given on days without carfilzomib (on Days 22 and 23 of Cycles 1-8) may be self-administered by the subject on an outpatient basis.
- If Day 2 of carfilzomib dosing is delayed (i.e., Day 2, 9, 16) 4 mg of dexamethasone premedication is required to be used prior to second treatment. Missed doses will not be replaced during a cycle. Missed doses of dexamethasone will not be made up.

6.3.4 Daratumumab IV Administration *(IV administration is discontinued and not available after the approval of version 7 of protocol dated February 20, 2023)*

Daratumumab can be administered by IV infusion or subcutaneous injection as assessed by the treating investigator. Method of daratumumab administration will be patient-specific and based on treating investigator's assessment.

Daratumumab (16 mg/kg) can be administered as an IV infusion. Each subject's dose will be calculated based on the subject's weight at Cycle 1 Day 1 rounded to the nearest kilogram. The dose of daratumumab will remain constant throughout the study, unless the subject's weight changes more than 10% from Cycle 1 Day 1. All infusions will be planned as outpatient visits. On daratumumab infusion days, subjects will receive the following medications 1 hour prior to infusion; however, premedication up to 3 hours before

the dose of daratumumab is permitted:

- oral antipyretics (acetaminophen 650 to 1000 mg)
- oral or intravenous antihistamine (diphenhydramine 25 to 50 mg or equivalent).

Daratumumab IV will be administered as follows:

- Cycle 1:16 mg/kg weekly **OR** Cycle 1 Day 1: 8 mg/kg Day 2: 8mg/kg, Day 8, 15, 22: 16 mg/kg
- Cycle 2: 16 mg/kg weekly
- Cycles 3-8: 16 mg/kg IV infusion every 2 weeks
- Cycles 9-24: 16 mg/kg IV infusion Day 1

The dilution volumes, initial infusion rates, and increment for the first, second, and subsequent doses are provided in Table 6-1. The first infusion, cycle 1, with a volume of 1,000 mL, takes approximately 8 hours or with a volume of 500 mL, takes approximately 4 hours; the second and subsequent infusions, with volumes of 500 mL, take approximately 4 hours. The maximum infusion rate for all infusions is 200 mL/hour for cycle 1 and any subjects experiencing a Grade 1 or greater infusion reaction. Beginning cycle 2, subjections without a Grade 1 or greater infusion reaction during cycle 1 may receive daratumumab over 90 minutes. Infuse at a rate so that 20% of the calculated dose is infused over the first 30 minutes (200 mL/hour). If tolerated, infuse the remaining 80% of the dose over 60 minutes (400 mL/hour), resulting in an estimated 90 minute
infusion (total volume=500 mL). The supporting company may modify the infusion rates or the pre-infusion medications prospectively based upon the information collected to date from this and other studies.

For all infusions, daratumumab will be prepared in sterile, pyrogen free 0.9% NaCl. Preparation of the infusion bags should be done on the day of the planned infusion. Daratumumab must be administered as an IV infusion given through a well-functioning IV catheter by using an infusion pump. The study drug must be filtered by using an inline filter (0.2 μM) during the infusion. Manuals with detailed descriptions for preparation and administration of daratumumab will be supplied to each pharmacy and site.

As noted in the Schedule of Events, all subjects should have vital signs monitored at the start and end of the infusion. If a subject experiences any significant medical event, then the investigator should assess whether the subject should stay overnight for observation. If the subject has not experienced a significant medical event but is hospitalized overnight only for observation, then the hospitalization should not be reported as a serious adverse event.

Patients with a history of obstructive pulmonary disorders may require additional post-infusion medications to manage respiratory complications. Consider prescribing short- and long-acting bronchodilators and inhaled corticosteroids for patients with obstructive pulmonary disorders.

The supporting company may modify the infusion rates (Table 6-1) prospectively based upon the information collected to date from this and other studies. Additional details for administration times and rates, as well as pre-infusion medications, will be provided in the administration guidelines.

Table 6-1 Daratumumab Infusion Rates

	Dilution volume	Initial rate (first hour)	Rate increment	Maximum rate
**First infusion**	1000 mL	50 mL/hour	50 mL/hour every hour	200 mL/hour
**Second infusion**[a]	500 mL	50 mL/hour	50 mL/hour every hour	200 mL/hour
**Subsequent infusions**[b]	500 mL	100 mL/hour	50 mL/hour every hour	200 mL/hour

a Escalate only if there were no Grade 1 (mild) or greater infusion reactions during the first 3 hours of the first infusion.

b Escalate only if there were no Grade 1 (mild) or greater infusion reactions during a final infusion rate of ≥100 mL/hr in the first two infusions.

6.3.5 Daratumumab Administration (Subcutaneous Injection)

Daratumumab will be administered by subcutaneous (SC) injection as a fixed-dosed of 1800 mg by manual push over 3 to 5 minutes in the abdominal SC tissues in left/right locations, alternating between individual doses. The volume of the SC solution will be 15 mL for the 1800 mg dose. The dose of daratumumab will remain constant throughout the study.

As noted in the Schedule of Events, all subjects will be observed for 1 hour after the first dose of subcutaneous daratumumab during Cycle 1 Day 1 and, if deemed necessary by the treating investigator,

after subsequent injections (all patients will be switching from the IV formulation; there will be no new starts). If a subject experiences any significant medical event, then the treating investigator should assess whether the subject should stay overnight for observation. If the subject has not experienced a significant medical event but is hospitalized overnight only for observation, then the hospitalization should not be reported as a serious adverse event. Reasons for continued observation on subsequent daratumumab injections may include but are not limited to the following: subjects with a higher risk of respiratory complications (eg, subjects with mild asthma or subjects with COPD who have an FEV1 < 80% at screening or developed FEV1 <80% during the study without any medical history), subjects with injection-related reactions (IRRs) with first injection of study drug, or subjects with a decreased condition on day of dosing compared to the prior dosing day.

Patients with a history of obstructive pulmonary disorders may require additional post-infusion medications to manage respiratory complications. Consider prescribing short- and long-acting bronchodilators and inhaled corticosteroids for patients with obstructive pulmonary disorders.

6.3.5.1 Daratumumab SC Dosing

Daratumumab SC injection will be administered as follows:

- Cycles 1-2: Days 1, 8, 15, 22
- Cycles 3-8: Days 1, 15
- Cycles 9-24: Day 1

All daratumumab administrations will be in an outpatient setting. Subjects will receive concomitant medications as outlined in Section 6.2.2.

Vital signs should be monitored extensively on Cycle 1 Day 1 before, and after the first administration of daratumumab. For all other administrations, vital signs should be measured before the start of injection and at the end of the injection. If the subject experiences any significant medical event, then the investigator should assess whether the subject should stay overnight for observation. If the subject has not experienced a significant medical event but is hospitalized overnight only for observation, then the hospitalization should not be reported as an SAE.

If an infusion-related reaction (IRR) develops, then the injection should be temporarily interrupted or slowed down. In the event of a life-threatening IRR (which may include pulmonary or cardiac events) or anaphylactic reaction, dara-SC should be discontinued, and no additional dara-SC should be administered to the participant. See Section 6.5.1 for instructions on the management of IRRs.

6.4 Instructions for Initiation of a New Cycle

A new course of treatment may begin on the scheduled Day 1 of a new cycle if all of the following are met:

- ANC $\geq$ 1.0 x 10^9/L
- Platelet count $\geq$ 30 x 10^9/L
- Any other study drug-related adverse event must have resolved to Grade 1 or baseline (see Appendix 2)
- Serum uric acid and creatinine concentrations must return to baseline prior to carfilzomib doses during Cycles 1 and 2

If ANC < 1.0 x 10^9/L but > 0.75 x 10^9/L, the start of a new cycle is at the discretion of the treating investigator

If these conditions are not met on Day 1 of a new cycle, the subject will be evaluated weekly, and a new treatment cycle will not be initiated until the toxicity has resolved, as described above.

If daratumumab, carfilzomib, or lenalidomide, or daratumumab are held for the remainder of the previous cycle or the new cycle is delayed due to residual toxicity on the planned Day 1 of the next cycle, then the new cycle will be started at 1 dose decrement.

If a delay of starting a new cycle is greater than 21 days, the subject should be discontinued from treatment, unless continuing treatment is mutually agreed upon by the site Lead Principal Investigator at the University of Chicago and the co-investigator at the treating institution.

Dexamethasone may be discontinued without the subject discontinuing study treatment.

6.5 Dose-modification guidelines

The following sections and tables summarize dosing modifications of daratumumab, carfilzomib, lenalidomide and dexamethasone to manage possible toxicity. Dose modifications different from those stated in the protocol should be discussed with the Lead Principal Investigator. Administration of daratumumab, carfilzomib and lenalidomide will be discontinued in the event of any other toxicity that, in the opinion of the lead, secondary site, or treating investigator, warrants discontinuation.

In addition to dose reductions, administration of daratumumab, carfilzomib and lenalidomide will be held temporarily in the event of a treatment-related toxicity at the treating investigator's discretion.

Study treatment may be reintroduced if resolution of the event to the baseline value or to ≤ Grade 1 within 21 days; otherwise study drug will be permanently discontinued. Any deviations from this plan must be approved by the Lead Principal Investigator.

All clinically-significant non-hematologic toxicities must be resolved to Grade 1 or baseline.

Individual dose modification of daratumumab is not permitted. Dosing can be delayed to manage daratumumab-related toxicities. Dose de-escalation of daratumumab will be based only on the Data and Safety Monitoring Board (DSMB) recommendation. Dose reduction levels of carfilzomib, lenalidomide and dexamethasone for toxicity management of individual subjects are provided below:

Table 6-2 Dose Reductions for Carfilzomib

**Nominal carfilzomib dose**	**Dose -1**	**Dose -2**	**Dose -3**	**Dose -4**
36 mg/m^2	27 mg/m^2	20 mg/m^2	15 mg/m^2	11 mg/m^2

Table 6-3 Dose Reductions for Lenalidomide

**Nominal Lenalidomide Dose**	**Dose -1**	**Dose -2**	**Dose -3**	**Dose -4**	**Dose -5**
25 mg Days 1-21	20 mg	15 mg	10 mg	5 mg	5 mg every other day

Table 6-4 Dose Reductions for Dexamethasone

Nominal Dexamethasone Dose	Dose -1	Dose -2	Dose -3
40 mg	32 mg	24 mg	20 mg
20 mg	12 mg	8 mg	4 mg

*Split dosing of dexamethasone on Days 1, 2, 8, 9, 15, 16, 22, 23 may be implemented to control toxicities that do not require a dose reduction. Split dosing requires the approval of the Lead Principal Investigator before implementing.

6.5.1 Toxicity Management Guidelines

Treatment guidelines for specific hematologic toxicities are outlined in Section 6.5.1.1 and non-hematologic toxicities in Section 6.5.1.2. In addition to dose reductions, administration of daratumumab, carfilzomib and/or lenalidomide may be held temporarily in the event of a treatment-related toxicity at the treating investigator's discretion.

6.5.1.1 Toxicity Management for Daratumumab

Infusion-Related Reactions

Infusion-related reactions (IRRs) are systemic reactions related to daratumumab administration. Participants should be observed carefully during daratumumab administrations. Trained study staff at the clinic should be prepared to intervene in case of any IRRs, and resources necessary for resuscitation (e.g., agents such as epinephrine and aerosolized bronchodilator, medical equipment such as oxygen tanks, tracheostomy equipment, and a defibrillator) must be available at the bedside. Attention to staffing should be considered when multiple participants will be dosed at the same time. If an IRR develops during Dara SC administration, then the administration should be temporarily interrupted. Participants who experience AEs during Dara-SC administration must be treated for their symptoms. Participants should be treated with paracetamol (acetaminophen), antihistamine, or corticosteroids, as needed. Intravenous saline may be indicated. For bronchospasm, urticaria, or dyspnea, participants may

require antihistamines, oxygen, corticosteroids, or bronchodilators. For hypotension, participants may require vasopressors. In the event of a life-threatening IRR (which may include pulmonary or cardiac events) or an anaphylactic reaction, Dara-SC should be discontinued.

Management of Infusion-Related Reactions and Local Injection Site Reactions

Infusion-related Reactions Grade 1 or Grade 2:

If the investigator assesses a Grade 1-2 IRR to be related to administration of study intervention, then the Dara-SC administration should be interrupted. When the participant's condition is stable, Dara-SC administration may be restarted at the investigator's discretion. Refer to the USPI for further details regarding continuation of Dara-SC administration.

If the participant experiences a Grade 2 or higher event of laryngeal edema, or a Grade 2 or higher event of bronchospasm that does not respond to systemic therapy and does not resolve within 6 hours from onset, then the participant must be permanently discontinued from Dara-SC treatment.

Infusion-related Reactions Grade 3 or Higher:

For IRR AEs (other than laryngeal edema or bronchospasm) that are Grade 3, the Dara-SC administration must be stopped, and the participant must be observed carefully until resolution of the AE or until the intensity of the event decreases to Grade 1, at which point the Dara-SC administration may be restarted at the investigator’s discretion. Refer to the USPI for further details regarding continuation of Dara-SC administration.

If the intensity of the AE returns to Grade 3 after restart of the Dara-SC administration, then the participant must be permanently discontinued from Dara-SC treatment.

For IRR AEs that are Grade 4, the Dara-SC administration must be stopped, and the participant permanently discontinued from Dara-SC treatment.

Recurrent Infusion-related Reactions:

If a Grade 3 IRR (or Grade 2 or higher event of laryngeal edema, or a Grade 2 or higher event of bronchospasm) recurs during or within 24 hours after a subsequent Dara-SC administration, the participant must be permanently discontinued from Dara-SC treatment.

Injection Site Reactions:

In clinical studies, SC administration of daratumumab was associated with local injection site reactions, such as induration and erythema, in some subjects. The reactions usually resolved within 60 minutes. Local injection-site reactions should be managed per institutional standards.

Individual dose modification of Dara SC is not permitted. For managing Dara SC-related toxicities, either a dose interruption or a dose delay is recommended.
Daratumumab must be held if any of the following criteria below are met, to allow for recovery from toxicity, regardless of relationship to daratumumab:

- Grade 4 hematologic toxicity, except for Grade 4 lymphopenia;
- Grade 3 or higher thrombocytopenia;
- Febrile neutropenia;
- Neutropenia with infection, of any grade;
- Grade 3 or higher non-hematologic toxicities with the following exceptions:
  - Grade 3 nausea that responds to antiemetic treatment within 7 days
  - Grade 3 vomiting that responds to antiemetic treatment within 7 days
  - Grade 3 diarrhea that responds to antidiarrheal treatment within 7 days
  - Grade 3 fatigue that was present at baseline or that lasts for <7 days after the last administration of daratumumab
  - Grade 3 asthenia that was present at baseline or that lasts for <7 days after the last administration of daratumumab

Other than on Day 1 of a cycle, if any ‘within-cycle’ daratumumab and hyaluronidase-fihj administration does not commence within the prespecified window (see table 6-5 below) of the scheduled administration date then the dose will be considered a missed dose.. Administration may resume at the next planned dose date. A missed dose will not be made up.

Delay of Day 1 drug dosing in any given cycle should not result in a skipped dose but should lead to a delay of the entire cycle instead. A minimum of 4 days between daratumumab doses

must be observed. If a dose is delayed, then the dates of all subsequent doses must be adjusted. If a dose delay occurs, then blood samples should be collected on the actual day of study drug administration, not on the original scheduled drug administration day.

Table 6-5 Daratumumab Administration Window

Cycles	Frequency	Dose Held	Dosing Re-start
1 and 2	Weekly (q1wk)	>3 days	next planned weekly dosing date
3 to 6	Biweekly (q2wks)	>7 days	next planned biweekly dosing date
7+	Every 4 weeks (q4wks)	>7 days	next planned every 4 weeks dosing date

If a dose is delayed, then the dates of all subsequent doses must be adjusted. Any dose hold of more than 14 days due to toxicity will result in permanent discontinuation of daratumumab. Dose holds of more than 14 days for other reasons should be discussed with the Lead Principal Investigator. If a dose delay occurs, then pharmacokinetic and pharmacodynamic assessments should be performed on the actual day of study drug administration, not on the original scheduled administration day.

For infusion-related reactions of any grade/severity, immediately interrupt the daratumumab infusion and manage symptoms. Management of infusion reactions may further require reduction in the rate of infusion, or treatment discontinuation of daratumumab as outlined below:

- Grade 1-2 (mild to moderate): Once reaction symptoms resolve, resume the infusion at no more than half the rate at which the reaction occurred. If the patient does not experience any further reaction symptoms, infusion rate escalation may resume at increments and intervals as appropriate (Table 6-1).

- Grade 3 (severe): If the intensity of the reaction decreases to Grade 2 or lower, consider restarting the infusion at no more than half the rate at which the reaction occurred. If the patient does not experience additional symptoms, resume infusion rate escalation at increments and intervals as outlined in Table 6-1. Repeat the procedure above in the event of recurrence of Grade 3 symptoms. Permanently discontinue daratumumab upon the third occurrence of a Grade 3 or greater infusion reaction.

- Grade 4 (life threatening): Permanently discontinue daratumumab treatment.

6.5.1.2 Hematologic Toxicity Management for Lenalidomide and Carfilzomib

Please refer to **Table 6-6** and **Table 6-7** below for dose modifications for lenalidomide and carfilzomib regarding hematologic toxicities.

Table 6-6 Dose Modification Guidelines for Hematologic Toxicities

When Platelets:	Lenalidomide	Carfilzomib	
Fall to < 30 x 10^9/L	• Hold dose, follow CBC weekly<br>• Hold prophylactic anticoagulation until platelets return to $\geq 30 \times 10^9$/L<br>• Then resume at 1 dose decrement	If platelets 10-30 x10^9/L without evidence of bleeding	• Hold<br>• With resolution restart at previous dose
		If evidence of bleeding or platelets < 10 x10^9/L	• Hold<br>• With resolution restart at previous dose
For each subsequent drop to < 30 x 10^9/L	• Hold dose, follow CBC weekly<br>• Hold prophylactic anticoagulation until platelets return to $\geq 30 \times 10^9$/L<br>• Then resume at additional dose decrement	If platelets 10-30 x 10^9/L without evidence of bleeding	• Hold<br>• With resolution restart at previous dose
		If evidence of bleeding or platelets < 10 x10^9/L	• Hold<br>• With resolution restart at 1 dose decrement
Grade 4 thrombocytopenia without evidence of bleeding, carfilzomib dosing may occur at the discretion of the treating investigator. However, subjects should receive supportive measures in accordance with institutional guidelines.			

Table 6-7 Dose Modification Guidelines for Hematologic Toxicities

When ANC:	Lenalidomide	Carfilzomib	
Fall to < 0.75 x 10^9/L	Hold dose, administer myeloid growth factor<br>Follow CBC weekly<br>Resume at full dose when ANC ≥ 0.75 x 10^9/L	If ANC 0.5-0.75 x 10^9/L	Continue at full dose
		If ANC < 0.5 x10^9/L	Hold<br>Resume at 1 dose decrement when ANC returns to ≥ 0.5 x10^9/L
For each subsequent drop to < 0.75 x 10^9/L	Hold dose, administer myeloid growth factor<br>Follow CBC weekly<br>Resume at 1 dose decrement when ANC ≥ 0.75 x 10^9/L	If ANC 0.5-0.75 x 10^9/L	Continue at full dose
		If ANC < 0.5 x10^9/L	Hold<br>Resume at 1 dose decrement when ANC returns to ≥ 0.5 x10^9/L

6.5.1.3 Non-hematologic Toxicity for Lenalidomide and Carfilzomib

Please refer to **Table 6-8** below for dose modifications for lenalidomide and carfilzomib regarding Non-hematologic toxicities.

Table 6-8 Dose Modifications for Non-hematologic Toxicity

Recommended Action		
	**Lenalidomide**	**Carfilzomib**
**Non-Blistering Rash**		
Grade 3	Hold lenalidomide dose; follow weekly<br><br>If the toxicity resolves to ≤ Grade 1 prior to Day 21 of the current cycle, restart at 1 dose decrement and continue the cycle until Day 21 of the current cycle.	Hold (if treating investigator's opinion is possibly related to carfilzomib) until ≤ Grade 1, reinstitute at current dose
Grade 4	Discontinue lenalidomide study drug.	Hold until ≤ Grade 1, reinstitute at current dose.
**Desquamating (blistering) rash `– any grade**	Discontinue lenalidomide study drug.	Hold until ≤ Grade 1, reinstitute at current dose.
**Erythema multiforme ≥ Grade 3**	Discontinue lenalidomide study drug.	Hold until ≤ Grade 1, reinstitute at current dose.
**Sinus bradycardia/ other cardiac arrhythmia**		
≤ Grade 2	Hold lenalidomide dose. Follow at least weekly.<br><br>If the toxicity resolves to ≤ Grade 1 prior to Day 21, restart at 1 dose decrement and continue the cycle until Day 21.	Hold until ≤ Grade 1, reinstitute at current dose.
≥ Grade 3	Discontinue lenalidomide study drug	Hold until ≤ Grade 1, reinstitute at current dose.
**Allergic reaction/hypersensitivity**		

Table 6-8 Dose Modifications for Non-hematologic Toxicity

Recommended Action		
	**Lenalidomide**	**Carfilzomib**
Grade 2 – 3	Hold lenalidomide dose. Follow at least weekly.<br><br>If the toxicity resolves to ≤ Grade 1 prior to Day 21, restart at 1 dose decrement and continue the cycle until Day 21.	Hold until ≤ Grade 1, reinstitute at current dose.
Grade 4	Discontinue	Discontinue
**Tumor lysis syndrome**<br><br>≥ 3 of the following: ≥ 50% increase in creatinine, uric acid, or phosphate;≥ 30% increase in potassium;≥ 20% decrease in calcium; or ≥ 2-fold increase in LDH	Hold lenalidomide until all abnormalities in serum chemistries have resolved.<br><br>Reinstitute at full doses.	Hold carfilzomib until all abnormalities in serum chemistries have resolved.<br><br>Reinstitute at full doses.
**Infection Grade 3 or 4**	Hold lenalidomide until systemic treatment for infection is completed. If no neutropenia, restart both drugs at full dose. If neutropenic, follow neutropenic instructions.	Hold carfilzomib until systemic treatment for infection is completed If no neutropenia, restart both drugs at full dose. If neutropenic, follow neutropenic instructions.
**Herpes zoster or simplex of any grade**	Hold lenalidomide until lesions are dry.<br>Reinstitute at full doses.	Hold carfilzomib until lesions are dry.<br>Reinstitute at full doses.
**Grade 2 neuropathy with pain or any Grade 3 neuropathy**	Hold until ≤ Grade 2.<br>Then restart lenalidomide at 1 dose decrement	Hold until resolved to ≤ Grade 2.<br>Then restart carfilzomib at 1 dose decrement

Table 6-8 Dose Modifications for Non-hematologic Toxicity

Recommended Action		
	**Lenalidomide**	**Carfilzomib**
**Grade 4 neuropathy**	Discontinue	Discontinue
**Renal dysfunction**		
Serum creatinine > 2 mg/dL	Base dose reduction on calculated GFR (below)	Base dose reduction on calculated GFR (below)
CrCl>50 mL/min	Full dose	Full dose
CrCl <50 mL/min<br>> 30 mL/min	Reduce lenalidomide to 10 mg every 24 h; may reinstate prior dose if, after 2 cycles, CrCl normalizes	Full dose
CrCl< 30 mL/min	Reduce lenalidomide to 15 mg every 48 h	Hold carfilzomib until CrCl> 30 mL/min; restart at 1 dose decrement
CrCl< 30 mL/min requiring dialysis	5 mg. Once daily. On dialysis days the dose should be administered following dialysis.	Hold until resolved to ≤ Grade 2. Then restart carfilzomib at 1 dose decrement

Table 6-8 Dose Modifications for Non-hematologic Toxicity

	Recommended Action	
	**Lenalidomide**	**Carfilzomib**
**Venous thrombosis/embolism**<br><br>**≥ Grade 3**	Hold lenalidomide dose and adjust anticoagulation regimen; re-start at treating investigator's discretion at full dose	Hold carfilzomib dose and adjust anticoagulation regimen until resolution; re-start at 1 dose level reduction recommended
**Hyperthyroidism or hypothyroidism**	Omit lenalidomide for remainder of cycle, evaluate, and initiate appropriate therapy.<br><br>Restart lenalidomide next cycle at 1 dose decrement	No adjustment required
Congestive heart failure (CHF)	Any subject with symptoms of CHF, whether or not lenalidomide related, must have the dose held until resolution or return to baseline. If CHF was felt to be lenalidomide related, reinstate by one dose decrement after return to baseline. If no resolution of CHF after 2 weeks, the subject will be withdrawn from the study.	Any subject with symptoms of CHF, whether or not carfilzomib related, must have the dose held until resolution or return to baseline. If CHF was felt to be carfilzomib related, reinstate by one dose decrement after return to baseline. If no resolution of CHF after 2 weeks, the subject will be withdrawn from the study.
**Hypertension including Hypertensive Crises**	NA	≥ Grade 3: carfilzomib attribution, hold drug until resolved to ≤ Grade 2. Resume at one level dose reduction
**Heart problems including rapid, strong or irregular heartbeat**<br><br>**Heart attack, reduced blood flow to the heart, abnormal amount of fluid between the heart and lining around the heart, and swelling/irritation of the lining around the heart**	NA	≥ Grade 3: carfilzomib attribution, hold drug until resolved to ≤ Grade 1. Resume at one level dose reduction

Table 6-8 Dose Modifications for Non-hematologic Toxicity

	Recommended Action	
	**Lenalidomide**	**Carfilzomib**
**Pericardial Effusion**	NA	≥ Grade 3: carfilzomib attribution, hold drug until resolved to Grade 1. Resume at one level dose reduction
**Pericarditis**	NA	≥ Grade 3: carfilzomib attribution, hold drug until resolved to Grade 1. Resume at one level dose reduction
**Thrombotic Microangiopathy (TMA)**	NA	Monitor for signs and symptoms of TTP/HUS. If the diagnosis is suspected, stop carfilzomib and evaluate. If the diagnosis of TTP/HUS is excluded, carfilzomib may be restarted. If carfilzomib related TMA is confirmed, carfilzomib should be discontinued permanently.
**Posterior Reversible Encephalopathy Syndrome (PRES)**	NA	Discontinue carfilzomib if PRES is suspected and evaluate.
**Pulmonary Hypertension**	NA	= Grade 2: carfilzomib attribution, Reduce drug: one level dose reduction<br><br>≥ Grade 3: carfilzomib attribution, hold drug until resolved to ≤Grade 2. Resume at one level dose reduction
**Pulmonary Toxicities: Interstitial Lung Disease (inc. pneumonitis), Acute Respiratory Failure, and Adult Respiratory Distress Syndrome (ARDS), cough, and cough with phlegm**	NA	≥ Grade 2 for Pneumonitis<br><br>≥ Grade 3 for ARDS<br><br>≥ Grade 4 for Respiratory Failure<br><br>carfilzomib attribution, hold drug until resolved to ≤Grade 1. Resume at one level dose reduction

Table 6-8 Dose Modifications for Non-hematologic Toxicity

Recommended Action		
	**Lenalidomide**	**Carfilzomib**
**Blood clot in the lungs, fluid in the lungs, bleeding in the lungs**	NA	≥ Grade 3: carfilzomib attribution, hold drug until resolved to ≤ Grade 1. Resume at one level dose reduction
**Gastrointestinal Perforation**	NA	≥ Grade 3: carfilzomib attribution, hold drug until resolved to Grade 1. Resume at one level dose reduction
**Hepatic Toxicities (≥ Grade 3 elevation of AST or ALT, Bilirubin, or other ≥ Grade 3 liver abnormalities)**	Hold drug until resolved to ≤Grade 1 or baseline. Resume at the same dose or reduced dose as appropriate. If tolerated, the reduced dose may be escalated to the previous dose at the discretion of the physician. Frequent monitoring of liver function should then be implemented.	Hold drug until resolved to ≤Grade 1 or baseline. Resume at the same dose or reduced dose as appropriate. If tolerated, the reduced dose may be escalated to the previous dose at the discretion of the physician. Frequent monitoring of liver function should then be implemented.
**Other non-hematologic toxicity assessed as lenalidomide-related ≥ Grade 3**	Hold lenalidomide dose. Follow at least weekly.<br><br>If the toxicity ≤ Grade 1 before Day 21 of the current cycle, restart at 1 dose decrement and continue until Day 21 of the current cycle	Full dose
**Other non-hematologic toxicity assessed as carfilzomib-related ≥ Grade 3**	Full dose	Hold carfilzomib dose until toxicity resolves to ≤ Grade 1 or baseline. Restart at 1 dose decrement
**Other non-hematologic toxicity assessed as drug-related ≥ Grade 3**	Hold treatment and restart at 1 dose decrement when toxicity has resolved to ≤ Grade 1 or baseline	Hold treatment and restart at 1 dose decrement when toxicity has resolved to ≤ Grade 1 or baseline

6.5.1.4 Toxicity Management for Dexamethasone

Please refer to **Table 6-9** below for dose modifications for Dexamethasone regarding toxicities related to Dexamethasone.

Table 6-9 Dose Modification Guidelines for Toxicity Related to Dexamethasone

BODY SYSTEM	SYMPTOM	RECOMMENDED ACTION
**Gastrointestinal**	Dyspepsia, gastric or duodenal ulcer, gastritis Grade 1-2 (requiring medical management)	Treat with H2 blockers, sucralfate, or omeprazole. If symptoms persist despite above measures, decrease dexamethasone dose by 1 dose level.
**Gastrointestinal**	≥ Grade 3 (requiring hospitalization or surgery)	Hold dexamethasone until symptoms adequately controlled. Restart at 1 dose decrement along with concurrent therapy with H2 blockers, sucralfate, or omeprazole. If symptoms persist despite above measures, discontinue dexamethasone permanently.
**Gastrointestinal**	Acute pancreatitis	Discontinue dexamethasone permanently.
**Cardiovascular**	Edema ≥ Grade 3 (limiting function and unresponsive to therapy or anasarca)	Diuretics as needed, and restart dexamethasone at 1 dose decrement; if edema persists despite above measures, decrease dose another level. Discontinue dexamethasone permanently if symptoms persist despite second reduction.
**Neurology**	Confusion or mood alteration > Grade 2 (interfering with function +/- interfering with activities of daily living)	Hold dexamethasone until symptoms resolve. Restart at 1 dose decrement. If symptoms persist despite above measures, discontinue dexamethasone permanently.
**Musculoskeletal**	Muscle weakness > Grade 2 (symptomatic and interfering with function +/- interfering with activities of daily living)	Decrease dexamethasone by 1 dose level. If weakness persists, decrease dose by 1 more dose level. Discontinue dexamethasone permanently if symptoms persist.
**Metabolic**	Hyperglycemia ≥ Grade 3	Treatment with insulin or PO hypoglycemic agents as needed. If uncontrolled despite above measures, decrease dose by 1 dose level until levels are satisfactory.

6.5.2 Response Evaluation

The first response assessment should be completed at screening (for stratification purposes) and the M-spike value will be compared to pre-induction (prior to transplant) M-spike to determine response. If the pre-induction value is not available, the closest value to initiation of induction will be used as baseline for

all assessments, including response at screening and every cycle while on protocol treatment thereafter. If M-spike is not available, the respective pre-treatment immunoglobulin level and for light-chain-disease-only subjects, involved free light chain level or 24-hr total protein level, will be used to assess response. If at any time throughout the treatment a complete response or better is suspected, a complete disease assessment should be performed to confirm response according to IMWG criteria:

Myeloma Disease Assessment – laboratory	M-protein determination:<br>• Serum protein electrophoresis and immunofixation<br>• Urine protein electrophoresis and immunofixation<br>• Serum free light chains and Hevylite test<br>• Serum quantitative immunoglobulins
Bone Marrow Biopsy	Quantify percent myeloma cell involvement, and obtain bone marrow aspirate for conventional cytogenetics and fluorescent *in situ* hybridization. For subjects who give consent for correlative samples, an additional aspirate sample should be collected at screening, at time of complete response and/or progressive disease (if applicable), at the end of Cycles 8, 24, and then yearly for as long as no PD up to 2 years. If a CR is suspected after Cycle 8, a bone marrow biopsy will be completed at Cycle 12 to confirm.
Radiographic Imaging	As per standard of care

Progressive disease requires 2 consecutive assessments made at any time before classification of relapse or progression and/or institution of new therapy when clinically possible.

6.6 Treatment Discontinuation

Subjects will be free to discontinue treatment or withdraw from the study at any time, for any reason, or they may be withdrawn/ removed if necessary in order to protect their health (see reasons for withdrawal below).

Patients will be removed from further treatment for the following reasons:

- Disease Progression
- Non-compliance with study procedures
- Subject no longer consents to participate in the study
- Intercurrent illness that interferes with study assessments
- Treatment-related toxicity requiring treatment discontinuation
- Incidence or severity of AEs that indicates a potential health hazard to the subject
- For the fourth occurrence of the same Grade ≥ 3 non-hematological toxicity
- A delay in treatment > 21 days unless approved by the Lead Principal Investigator, or due to congestive heart failure unresolved for > 28 days the treatment shall be discontinued (see Table 6-8)
- Treating investigator discretion
- Requirement for alternative therapy
- Suspected or positive pregnancy
- Termination of the study by the sponsor

The Lead Principal Investigator should be contacted regarding any impending discontinuation of a study subject. If the reason for withdrawal is the occurrence of an AE, the subject will be followed until such events resolve, stabilize, and, according to the treating investigator's judgment, there is no need for further follow up. The reason for withdrawal from study must be documented in the case report form.

Any Grade 5 toxicity related to study treatment will prompt stopping of the trial until further review is completed. Additionally, development of Grade 4 of non-hematologic toxicities in 2 or more patients will prompt the DSMB to stop the study for review to determine whether the trial should be terminated due to excessive toxicity.

In case of premature discontinuation of the study treatment, the investigations scheduled for the EOT should be performed, if possible. Should a patient decide to withdraw, every effort will be made to complete and report the observations as thoroughly as possible. The treating investigator should contact the patient to determine as completely as possible the reason for the withdrawal. A complete final evaluation at the time of the patient's withdrawal should be made, with an explanation of why the patient is withdrawing from the study.

6.6.1 Duration of Follow Up

Long-term follow up will include an assessment for disease progression in subjects who did not progress during treatment. This should occur every 3 months (+/- 30 days) for 5 years from safety follow-up visit (28 days post-last study treatment). Subjects who do progress will be followed for survival for 2 years from the completion of the safety follow-up visit or until death, whichever occurs first. This should occur every 3 months (+/- 30 days) and can be done over the phone.

6.7 Safety Considerations & Supportive Care

Supportive measures for optimal medical care shall be provided during participation in this clinical trial. Supportive care including anti-nausea / anti-emetic therapy, acid suppression (proton pump inhibitors and/or H2-blockers), glucocorticoids, and other standard treatments may be administered as per institutional guidelines for symptomatic patients. As needed and per individual study site institutional guidelines, prophylactic therapies, including antivirals, antifungals, and antibiotics, may be administered to ameliorate risks associated with non-malignant disorders or of immune system compromise.

6.7.1 First Dose Effect (Carfilzomib)

A "first dose effect" has been seen, which is notable for fever, chills, rigors, and/or dyspnea occurring during the evening following the first day of infusion and an increase in creatinine on Day 2, which may be the clinical sequelae of rapid tumor lysis and/or cytokine release.

All subjects should be well hydrated Section 6.2.1. Clinically-significant electrolyte abnormalities should be corrected prior to dosing with carfilzomib. Renal function must be monitored closely during treatment.

Should a "first dose" effect occur at any point during Cycle 1 or 2, treatment with high dose glucocorticoids (e.g. methylprednisolone 50–100 mg) is recommended. In addition, intravenous fluids,

vasopressors, oxygen, bronchodilators, and acetaminophen should be available for immediate use and instituted, as medically indicated.

6.7.2 Safety Information for Carfilzomib

Hepatitis B Virus Reactivation

Cases of Hepatitis B Virus (HBV) reactivation have been reported in patients receiving Kyprolis.

Patients should be tested for HBV infection before initiating treatment. For patients who are carriers of HBV, prophylaxis with antivirals should be considered. Carriers of HBV who require treatment with Kyprolis should be closely monitored for signs and symptoms of active HBV infection throughout treatment and following the end of treatment.

Consider consulting a specialist for patients who test positive for HBV infection prior to or during treatment. The safety of resuming Kyprolis after HBV reactivation is adequately controlled is not known. Therefore, prescribers should weigh the risks and benefits when considering resumption of therapy in this situation.

Progressive Multifocal Leukoencephalopathy

Cases of Progressive Multifocal Leukoencephalopathy (PML) have been reported in patients treated with Kyprolis who have had prior or concurrent immunosuppressive therapy. The casual relationship with Kyprolis is unknown.

Patients should be monitored for any new or worsening neurologic, cognitive or behavioral signs or symptoms that may be suggestive of PML as part of the differential diagnosis of CNS disorders.

If PML is suspected, patients should be promptly referred to a specialist and appropriate diagnostic testing should be initiated. Discontinue Kyprolis if PML diagnosis is confirmed.

6.7.3 Tumor Lysis Syndrome (TLS)

TLS, which may be associated with multi-organ failure, has been observed in treatment Cycles 1 and 2 in some patients with MM who have been treated with carfilzomib. All subjects should follow the hydration

guidelines outlined in Section 6.2.1. If subjects are considered to be at risk for TLS, hydration should be continued into Cycle 2 if clinically indicated.

MM subjects with high tumor burden (e.g., Durie-Salmon or ISS Stage II/III), rapidly increasing M-protein or light chains, or compromised renal function (CrCl < 50 mL/min) should be considered to be at particularly high risk.

During Cycles 1 and 2, serum electrolytes and chemistries are closely monitored as outlined in Section 7.1.2. Subjects with laboratory abnormalities consistent with lysis of tumor cells (e.g. serum creatinine ≥ 50% increase, LDH ≥ 2-fold increase, uric acid ≥ 50% increase, phosphate ≥ 50% increase, potassium ≥ 30% increase, calcium ≥ 20% decrease) prior to dosing should not receive the scheduled dose. Subjects with such abnormalities should be re-evaluated as clinically indicated. The Lead Principal Investigator should be consulted if there are further delays.

If TLS occurs, cardiac rhythm, fluid, and serial laboratory monitoring should be instituted. Correct electrolyte abnormalities, monitor renal function and fluid balance, and administer therapeutic and supportive care, including dialysis, as clinically indicated.

All cases of TLS must be reported to the Lead Principal Investigator (who will, in turn be responsible for distributing this information to all sites) and to Amgen as a Serious Adverse Event (SAE) through the normal process within 24 hours of the clinical site becoming aware of the event.

6.7.4 Renal Function

Carfilzomib has not been fully characterized in subjects with creatinine clearance < 30 mL/min. It is critical that the subject's renal function is known at the time of dosing. Renal function, serum creatinine, and serum uric acid should be monitored closely during treatment with carfilzomib. Renal function must be monitored closely during treatment with carfilzomib. Serum chemistry values, including creatinine, must be obtained and reviewed prior to each dose of carfilzomib during Cycles 1 and 2. Refer to Table 6-8 for guidance regarding dose reduction in subjects with compromised renal function.

7 SAMPLES FOR MRD EVALUATION AND CORRELATIVE SAMPLES

Bone marrow samples for MRD evaluation are required and as per the standard of care and will be collected prior to initiation of any MM treatment (pre-induction), screening, at the completion of 8 cycles

of D-KRD, at 24 months and then yearly, or at the time of progression/relapse for up to 2 years. If a CR is suspected after Cycle 8, a bone marrow biopsy and MRD testing will be completed at Cycle 12.

Additionally, provided that the patient consents, correlative samples will be collected and banked for further research studies.

**MRD sample**<br><br>Bone Marrow Aspirate (MRD samples) or FFPE/BMA slides (ID/calibration sample from pre-treatment)	**Correlative Sample**	**Time Points**
	Bone Marrow Aspirate	Pre-treatment (calibration sample for MRD only), screening, at end of 8, and 24 cycles, or EOT, yearly after EOT up to 2 years, and at the time of complete response (if applicable: If a CR is suspected after cycle 8, a bone marrow biopsy and MRD testing will be completed at cycle 12 to confirm.)<br>Progression/Relapse
	Peripheral Blood	Screening, at end of 8 and 24 cycles or EOT, yearly after EOT up to 2 years, and at the time of complete response (if applicable; If a CR is suspected after cycle 8, a bone marrow biopsy and MRD testing will be completed at cycle 12 to confirm)<br>Progression/Relapse*
	Plasma/Serum	Screening, at end of 8 and 24 cycles or EOT, yearly after EOT up to 2 years, and at the time of complete response (if applicable; If a CR is suspected after cycle 8, a bone marrow biopsy and MRD testing will be completed at cycle 12 to confirm).<br>Progression/Relapse
	Buccal Swab	Screening only

*Correlative samples only

All bone marrow procedures (including those performed at screening, response and/or progression/relapse) are considered standard of care and a subject may give consent to have extra research samples collected at these visits.

Please refer to the Laboratory Manual for detailed processing and shipping instructions.

Label all specimens with the following:

1. Subject initials
2. Subject study number (will include protocol number)
3. Visit at which sample was drawn (i.e. C1D4)
4. Date sample drawn (i.e. mm/dd/yyyy)
5. Time sample drawn (24-hour clock)
6. Sample type (e.g. plasma, serum, bone marrow cells, tumor cells)

Shipping Instructions in the US:

1. An inventory sheet including a complete list of samples shipped (patient number, time point, study #) must accompany each shipment.
2. An electronic copy (Word or Excel) of the sample list must also be sent via email. The listing must also include a contact name, address and phone number of the person who is responsible for the shipment. They should sign and date the form.
3. Please contact lab technician to alert him/her of an incoming shipment by email:

   [redacted]

[redacted]

[redacted]

[redacted] 773-834-1592

Note: Please follow your institution's policy regarding destruction of patient samples upon withdrawal of informed consent.

8 ADVERSE EVENTS

An AE is any untoward medical occurrence in a study subject administered an investigational product and that does not necessarily have a causal relationship with this treatment.

An AE therefore can be any unfavorable and unintended sign (including laboratory finding), symptom or disease temporally associated with participation in an investigational study, whether or not considered drug-related. In addition to new events, any increase in the severity or frequency of a pre-existing condition that occurs after the subject signs a consent form for participation is considered an AE. This includes any side effect, injury, toxicity, or sensitivity reaction.

An unexpected AE is any adverse drug event, the specificity or severity of which is not consistent with the current IB or prescribing information for a marketed compound. Also, reports which add significant information on specificity or severity of a known, already documented AE constitute unexpected AEs. For example, an event more specific or more severe than described in the IB would be considered "unexpected".

Whenever possible, the Common Terminology Criteria for Adverse Events (CTCAE) version 4.0 should be used to describe the event and for assessing the severity of AEs (see Appendix 3). Any events representing a change in the CTCAE grade need to be reported on the AE case report form. This includes any change in laboratory values.

For AEs not adequately addressed in the CTCAE, the severity table below may be used:

Severity	Description
GRADE 1 – Mild	Transient or mild discomfort; no limitation in activity; no medical intervention/therapy required.
GRADE 2 – Moderate	Mild to moderate limitation in activity—some assistance may be needed; no or minimal medical intervention/therapy required.
GRADE 3 – Severe	Marked limitation in activity, some assistance usually required; medical intervention/therapy required, hospitalizations possible.
GRADE 4 – Life-threatening	Extreme limitation in activity, significant assistance required; life-threatening (immediate risk of death); significant medical intervention/therapy required, hospitalization or hospice care probable.
GRADE 5 – Fatal	Death

Any condition, laboratory abnormality, or physical finding with an onset date prior to the subject signing consent for study participation is considered to be pre-existing in nature and part of the subject's medical history.

8.1 Causality

Using the following criteria, the relationship of the AE to the study drug should be assessed as follows:

Definite (5) – The AE *is clearly related* to the study treatment.

Probable (4) – The AE *is likely related* to the study treatment.

Possible (3) – The AE *may be related* to the study treatment.

Unlikely (2) – The AE *is doubtfully related* to the study treatment.

Unrelated (1) – The AE *is clearly NOT related* to the study treatment.

All AEs will be considered for dose-limiting toxicity evaluation unless the event can clearly be determined to be unrelated to the drug.

8.2 Adverse Event Reporting Procedures

Information about all AEs, whether volunteered by the subject, discovered by the treating investigator questioning, or detected through physical examination, laboratory tests or other means, will be collected and recorded in eVelos and followed as appropriate.

All adverse events **must** be reported in routine study data submissions to the Lead PI who will review and will be responsible for alerting all participating sites about the AE as required. **AEs reported using the Serious Event Reporting Form and/or MedWatch Form discussed below must also be reported in routine study data submissions in eVelos.** Details of the event must include severity, relationship to study drug, duration, action taken, and outcome as shown on the eCRF in eVelos. All AEs must be followed to resolution or stabilization regardless of relationship to study drug.

All AEs that are considered related to study regimen must be followed to resolution or stabilization if improvement is not expected.

AEs must be reported from the date the subject signs consent through 30 days post-last dose of study treatment or initiation of a new anticancer therapy, whichever occurs first. If a subject is enrolled but discontinues study prior to receiving any study drug, only SAEs that are considered related to study procedures must be reported through the end-of-study visit. AEs that completely resolve and then recur should be recorded as a new AE. For subjects who complete the end of study visit less than 30 days following their last dose of study drug, a follow up of ongoing AEs should be attempted by telephone, and documented in the subject's source. AEs continuing at 30 days post-last dose should have a comment in the source by the treating investigator that the event has stabilized or is not expected to improve. SAEs continuing at 30 days post-last dose should be followed until resolution or stabilization.

The treating investigator is responsible for evaluating all AEs for relationship to study drug and for seriousness, obtaining supporting documents, and determining that documentation of the event is adequate. Adverse events will be assigned a severity grade using the NCI-CTCAE grading scale v4.0. The

treating investigator must assess all abnormal laboratory results for their clinical significance. Only Grade 1 and 2 abnormal laboratory values that are clinically significant, related to study treatment, and/or requiring concomitant medication will be recorded. If any abnormal laboratory result is considered clinically significant, the treating investigator must provide details about the action taken with respect to the test drug and about the patient's outcome. All Grade 3 and 4 laboratory abnormalities must be recorded as AEs on the CRF. Grade 1 and 2 abnormalities should only be recorded if they require treatment or are otherwise considered clinically significant by the treating investigator.

The Lead Principal Investigator may delegate these duties to sub-investigators and must ensure that these sub-investigators are qualified to perform these duties under the supervision of the Lead Principal Investigator, and that they are listed on the delegation log.

8.3 Serious Adverse Events

Information about all serious adverse events (SAE) will be collected independently.

All SAEs from US sites will be collected by the UC CRA and reviewed by the Lead Principal Investigator. Only those determined to be SUSARs by the Lead Principal Investigator will be distributed to all participating sites (using the completed MedWatch 3500 form). To ensure patient safety, each serious adverse event occurring in the US must be reported to the Lead Principal Investigator and he or a designee will in turn, report to the University of Chicago Comprehensive Cancer Center via entry on the SAE eCRF in eVelos within 24 hours of learning of its occurrence. In the event that direct entry into eVelos is not available, a paper SAE form is provided for this study and may be used and sent via fax or email.

The Lead Principal Investigator is responsible for notifying the ethics committees (ECs), and investigators, of any expedited, annual, or other periodic safety reports in accordance with applicable regulations.

The Site Investigator is also responsible for notifying the local ECs in accordance with local regulations. Additionally, the Lead Principal Investigator is responsible for reporting SAEs to Amgen and Celgene as described in Section 8.4.

8.3.1 Serious Adverse Event Definition

An SAE is one that meets the following criteria:

- Results in death
- Life-threatening experience defined as any adverse experience that places the subject, in the view of the treating investigator, at immediate risk of death at the time of occurrence; i.e., it does not include a reaction that, had it occurred in a more severe form, might have caused death.
- Requires inpatient hospitalization or prolongation of an existing hospitalization (except scheduled hospitalizations for non-acute, unrelated cause such as an elective surgery)
- Results in persistent or significant disability/incapacity or any other medicinal product effects, which the doctor by his state of knowledge deemed serious
- Is a congenital anomaly/birth defect in the offspring of an exposed subject
- Is a suspected transmission of any infectious agent via a medicinal product
- Important medical events that may not result in death, be life-threatening, or require hospitalization, may be considered an SAE, when, based upon appropriate medical judgment, it jeopardizes the subject and may require medical or surgical intervention to prevent one of the outcomes listed in this definition.

Any death occurring within 30 days of the subject receiving study drug, regardless of the subject having discontinued from the study must be reported to the Lead Principal Investigator as an SAE.

ALL Serious Adverse Events MUST be reported to the Lead Principal Investigator and to the University of Chicago Comprehensive Cancer Center (UC CCC), via entry on the SAE eCRF in eVelos or paper submission via fax or email. This is whether or not they are considered related to the study agent. Refer to Section 8.3.3 for reporting guidelines.

8.3.2 Serious and Unexpected Suspected Adverse Reaction (SUSAR)

A serious adverse event is considered to be a suspected adverse reaction if there is evidence to suggest a causal relationship to the study agent. This may include a single occurrence of an event strongly associated with drug exposure (e.g. Stevens Johnson Syndrome), one or more occurrence of an event otherwise

uncommon in the study population, or an aggregate analysis of specific events occurring at greater frequency than expected from historical controls.

Unexpected events are those not listed at the observed specificity or severity in the protocol, consent, Investigator brochure, or FDA package insert. This includes adverse events listed in the protocol or consent as occurring within the class of drugs or otherwise expected from the drug's pharmacological properties but which have not been previously observed with this agent.

The lead institution (University of Chicago) is responsible for notifying all participating investigators, when required, and in accordance with applicable laws and regulations of any Expedited Safety Reports that are determined by the Lead Principal Investigator to be Unexpected.

8.3.3 Serious Adverse Event Reporting and Documentation Requirements

8.3.3.1 Serious Adverse Event Reporting to the Coordinating Center

Use the UC CCC protocol number and the protocol-specific patient ID assigned during trial registration on all reports.

All serious adverse events (as defined above) occurring on this study require expedited reporting to the University of Chicago Comprehensive Cancer Center (UC CCC).

SAEs will be reviewed by the Lead Principal Investigator, who will determine whether they are SUSARs. The Lead Principal Investigator or designee will report all SUSARs to the UC CRA and UC CCC, who will report simultaneously to the UC IRB, and other US sites according to regulations (see below).

The responsible Research Nurse or other designated individual at the treating site should report the SAE to the Study Lead Investigator, the University of Chicago CRA and the UC CCC Cancer Clinical Trials Office (CCTO) by the end of the business day when s/he becomes aware of the event. Events occurring after business hours should be reported to the CCTO by 12pm (noon) the next business day. Reports should be made using the 'Serious Event Report' Form. Please scan and send via email (preferred) or fax to the following:

University of Chicago Phase II CRA General:

PhaseIICRA@medicine.bsd.uchicago.edu

Fax: 773-702-4889

UC CCC Cancer Clinical Trials Office Quality Assurance:

qaccto@bsd.uchicago.edu

All serious adverse events should also be reported to the local IRB of record according to their policies and procedures.

8.3.3.2 Serious and Unexpected Adverse Event Reporting by the Coordinating Center

The designated UC CCC Regulatory Manager will notify all participating sites of all SUSARs that occur on this clinical trial and which are reported to the UC Institutional Review Board (IRB).

SAE Reporting by the Lead Principal Investigator to Amgen

The Lead Principal Investigator must inform Amgen in writing by e-mail or fax at the contact information listed below for all SUSARs that are judged as reasonably related to the Amgen study drug. Site will transmit the final MedWatch form of that event to Amgen within twenty-four (24) hours. A line listing of SAEs should also be submitted to Amgen every 6 to 12 months in accordance with Amgen's SAE reporting requirements.

For regulatory reporting purposes, an event of "Death, Cause Unknown" from the study shall be processed as a SUSAR. All forms must be completed and provided to Amgen in English.

The Individual Case Safety Report (ICSR) may be referred to as an individual safety report or SAE Report, including Pregnancy Exposure Reports and Follow up Reports. The ICSR must be as complete as possible, at a minimum including event reference number, protocol name and number, investigator contact information, specific patient identifiers (e.g., initials, patient number, date of birth or age, or gender), the name of the suspect study drug, the date and dosage(s) of exposure, event, the date(s) of event, country of event, "Serious" criteria, relationship/causality of study drug, hospitalization history for the event, event

status/outcome, relevant history (including diagnostics, laboratory values, radiographs, concomitant medications, and event treatment, and narrative summary.

The Lead Principal Investigator shall be responsible for collecting all SAEs and Pregnancy and Lactation Exposure Reports and will exercise commercially reasonable due diligence to obtain follow-up information on incomplete SAE or Pregnancy and Lactation Exposure Reports. In the event that Amgen requires clarification or further information on individual SAE or Pregnancy and Lactation Exposure Reports, Amgen will not contact non-party investigators directly, but will route all such inquiries through the Lead Principal Investigator for forwarding to such investigator(s). The Lead Principal Investigator will be responsible to ensure such inquiries are completed and provided in a timely manner to Amgen.

Information not available at the time of the initial report (e.g., an end date for the SAE, discharge summaries, lot numbers, relevant laboratory values, scan data and autopsy reports, which are received after the initial report) must be documented on a follow-up form, and submitted to Amgen in the same timelines as outlined above. The Lead Principal Investigator shall be responsible for obtaining follow-up information for the SAEs and demonstrate diligence in attempting to obtain such information by, among other things, maintaining written records of such attempts.

Other aggregate analysis including reports containing safety data generated during the course of the study is to be submitted to Amgen at the time the Lead Principal Investigator submits to anybody governing research conduct i.e. RA, IRB etc. Final study report and reports of unauthorized use of a marketed product to be submitted to Amgen at the time the Lead Principal Investigator ISS submits to anybody governing research conduct i.e. RA, IRB etc. but not later than one calendar year of study completion.

Reports containing safety data generated during the course of the study are to be submitted to Amgen at the time the Lead Principal Investigator submits to anybody governing research conduct, i.e. regulatory authorities and IRBs. The Lead Principal Investigator will support reconciliation of all ICSRs at the end of the study at a minimum.

The Amgen protocol number **(20167943)** and the institutional protocol number should be included on SAE reports to Amgen.

Amgen Drug Safety and Pharmacovigilance Contact Information:

Amgen Global Safety
Toll-free #:1-888-814-8653
For countries where the U.S. toll-free # cannot be used: +44-20-7136-1046
Email (Only for sponsors with a secure email connection with Amgen):
svc-ags-in-us@amgen.com

SAE Reporting by Lead Principal Investigator to Celgene

For the purpose of regulatory reporting, Celgene Drug Safety will determine the expectedness of events of being related to lenalidomide based on the Investigator Brochure. In the United States, all suspected unexpected serious adverse reactions (SUSARs) will be reported in an expedited manner in accordance with 21 CFR 312.32.

Serious adverse events (SAE) are defined above. The investigator must inform Celgene in writing using a Celgene SAE form or MEDWATCH 3500A form of any SAE within 24 hours of being aware of the event. The written report must be completed and supplied to Celgene by facsimile within 24 hours. The initial report must be as complete as possible, including an assessment of the causal relationship between the event and the investigational product(s). Information not available at the time of the initial report (e.g., an end date for the adverse event or laboratory values received after the report) must be documented on a follow-up report. A final report to document resolution of the SAE is required. The Celgene protocol number (RV-CL-MM-PI-005610) and institutional numbers should be included on SAE reports to Celgene. A copy of the fax transmission or email confirmation of the SAE report (or on the fax cover letter) sent to Celgene should be attached to the SAE and retained with the patient records.
Participating study sub-sites must report SAEs to Celgene as described and within 24 hours of awareness. Participating sub-sites must also report SAEs to the University of Chicago as described in Section 8.3.3.2.

Celgene Drug Safety and Pharmacovigilance Contact Information:

Celgene Corporation
Global Drug Safety and Risk Management
556 Morris Avenue
Building S12
Summit, New Jersey 07901
Fax: (908) 673-9115 E-mail: drugsafety@celgene.com

SAE Reporting by the Lead Principal Investigator to Janssen Scientific Affairs, LLC

Overview

As the sponsor of the study, the Lead Principal Investigator shall be solely responsible for complying, within the required timelines, any safety reporting obligation to competent health authorities, IRB/ECs and any participating (co or sub) investigators, as defined in applicable laws and regulations. For the purposes of this section, safety data includes adverse events, product quality complaints (PQCs), and special situations including pregnancies.

The Lead Principal Investigator will provide safety information to Janssen Scientific Affairs, LLC on adverse events, special situations including pregnancies and product quality complaints as defined within this section. Additionally, MedWatch Forms will be used to report SAEs.

Management of Safety Data

This study has been designated as an interventional study. As such, all adverse events for Janssen medicinal products regardless of causality and special situations excluding those from subjects not exposed to a Janssen medicinal product and product quality complaints with or without an adverse event as described in this section will be reported from the time a subject has signed and dated an informed consent form until completion of the subject's last study-related procedure (which may include contact for follow-up safety). Serious adverse events will be reported for 30 days after the last dose of study drug.

For the purposes of this study, the Janssen medicinal product is: DARZALEX™ (daratumumab)

Definitions

Adverse Events of Special Interest

Adverse events of special interest are events that Janssen Scientific Affairs, LLC is actively monitoring as a result of a previously identified signal (even if non-serious). These adverse events are:

- Infusion reactions: ≥ Grade 3
- Infections: ≥ Grade 4
- Cytopenias: ≥ Grade 4

- HBV reactivation
- Other malignancies

Any adverse event of special Interest that is to be reported to Janssen should be recorded on a Serious Adverse Event Report Form and be reported to Janssen within 24 hours of knowledge of the event.

Individual Case Safety Report (ICSR)

A valid ICSR must contain the four minimum criteria required to meet regulatory reporting requirements.

- an identifiable subject (but not disclosing personal information such as the subject's name, initials or address)
- an identifiable reporter (investigational site)
- a Janssen medicinal product
- an adverse event, outcome, or certain special situations

The minimum information required is:

- suspected Janssen medicinal product (doses, indication)
- date of therapy (start and end date, if available)
- batch or lot number, if available
- subject details (subject ID and country)
- gender
- age at AE onset
- reporter ID
- adverse event detail (AE verbatim in English), onset date, relatedness, causality, action taken, outcome, (if available)
- Janssen protocol ID

Product Quality Complaint (PQC)

A product quality compliant is defined as any suspicion of a product defect related to a potential quality issue during manufacturing, packaging, release testing, stability monitoring, dose preparation, storage or distribution of the product, or delivery system. Not all PQCs involve a subject. Lot and batch numbers are of high significance and need to be collected whenever available.

Examples of PQC include but not limited to:

- Functional Problem: e.g., altered delivery rate in a controlled release product
- Physical Defect: e.g. abnormal odor, broken or crushed tablets/capsules
- Potential Dosing Device Malfunction: e.g., autoinjector button not working, needle detaching from syringe
- Suspected Contamination
- Suspected Counterfeit

*Medical and scientific judgment should be exercised in deciding whether expedited reporting is also appropriate in other situations, such as important medical events that may not be immediately life threatening or result in death or hospitalization but may jeopardize the subject or may require intervention to prevent one of the other outcomes listed in the definition above. These should usually be considered serious and should be reported using a MedWatch Form.

NOTE: DEATH FOR ANY REASON SHOULD BE REPORTED AS A SERIOUS ADVERSE EVENT.

Hospitalization

For reports of hospitalization, it is the sign, symptom or diagnosis which led to the hospitalization that is the serious event for which details must be provided.

Any event requiring hospitalization or prolongation of hospitalization that occurs during the study must be reported as a serious adverse event, except hospitalizations for the following:

- Hospitalizations not intended to treat an acute illness or adverse event (e.g., social reasons such as pending placement in long-term care facility)
- Surgery or procedure planned before entry into the study. [Note: Hospitalizations that were planned before the start of data collection and where the underlying condition for which the hospitalization was planned has not worsened will not be considered serious adverse events. Any adverse event that results in a prolongation of the originally planned hospitalization is to be reported as a new serious adverse event.]

- [For convenience the investigator may choose to hospitalize the subject for the duration of the treatment period.]

Life-Threatening Conditions

Disease progression should not be recorded as an adverse event or serious adverse event term; instead, signs and symptoms of clinical sequelae resulting from disease progression/lack of efficacy will be reported if they fulfill the serious adverse event definition.

Unlisted (Unexpected) Adverse Event/Reference Safety Information

An adverse event is considered unlisted if the nature or severity is not consistent with the applicable product reference safety information. For a medicinal product(s) with a marketing authorization, the expectedness of an adverse event will be determined by whether or not it is listed in the applicable product information.

http://www.darzalex.com/shared/product/darzalex/darzalex-prescribing-information.pdf

For DARZALEX™ (daratumumab), the expectedness of an adverse event will be determined by whether or not it is listed in the Investigator's Brochure

Special Reporting Situations

Safety events of interest for a Janssen medicinal product that require expediting reporting and/or safety evaluation include, but are not limited to:

- Drug exposure during pregnancy (maternal and paternal)
- Overdose of a Janssen medicinal product
- Exposure to a Janssen medicinal product from breastfeeding
- Suspected abuse/misuse of a Janssen medicinal product
- Inadvertent or accidental exposure to a Janssen medicinal product
- Any failure of expected pharmacological action (i.e., lack of effect) of a Janssen medicinal product
- Medication error involving a Janssen medicinal product (with or without patient exposure to the Janssen medicinal product, e.g., name confusion)

- Suspected transmission of any infectious agent via administration of a medicinal product
- Unexpected therapeutic or clinical benefit from use of a Janssen medicinal product

These safety events may not meet the definition of an adverse event; however, from a Janssen Scientific Affairs, LLC perspective, they are treated in the same manner as adverse events. Special situations should be recorded on the Adverse Event page of the CRF.

Any special situation that meets the criteria of a serious adverse event should be recorded on a Serious Adverse Event Report (or MedWatch) Form and be reported to Janssen Scientific Affairs, LLC within 24 hours of becoming aware of the event.

Maintenance of Safety Information

All safety data should be maintained in a clinical database in a retrievable format. The Lead Principal Investigator shall provide all adverse events, both serious and non-serious, in report format. However, in certain circumstances more frequent provision of safety data may be necessary, e.g. to fulfill a regulatory request, and as such the data shall be made available within a reasonable timeframe at Janssen Scientific Affairs, LLC request.

Procedures for Reporting Safety Data and Product Quality Complaints (PQCs) for Janssen Medicinal Products to Janssen Scientific Affairs, LLC

All adverse events and special situations, whether serious or non-serious, related or not related, following exposure to a Janssen medicinal product are to be documented by the investigator and recorded in the CRF and in the subject's source records. Investigators must record in the CRF their opinion concerning the relationship of the adverse event to a Janssen medicinal product.

All (serious and non-serious) adverse events reported for a Janssen medicinal product should be followed-up in accordance with clinical practice.

SAEs and Special Reporting Situations

All serious adverse events that have not resolved by the end of the study, or that have not resolved upon discontinuation of the subject's participation in the study, must be followed until any of the following occurs:

- The event resolves
- The event stabilizes
- The event returns to baseline, if a baseline value/status is available
- The event can be attributed to agents other than the study drug or to factors unrelated to study conduct
- It becomes unlikely that any additional information can be obtained (subject or health care practitioner refusal to provide additional information, lost to follow-up after demonstration of due diligence with follow-up efforts)

The Lead Principal Investigator will transmit all SAEs and special situations following exposure to a Janssen product under study in a form provided by Janssen Scientific Affairs, LLC in accordance with Section 10, Transmission Methods, in English within 24-hours of becoming aware of the event(s).

In the event the study is blinded, the Lead Principal Investigator will submit an unblinded SAE or pregnancy exposure report to Janssen Scientific Affairs, LLC.

All follow-up information for serious adverse events that are not resolved at the end of the study or by the time of patient withdrawal must be reported directly by the Lead Principal Investigator, within 24 hours becoming aware, to Janssen Scientific Affairs, LLC using the Janssen Scientific Affairs, LLC Serious Adverse Event Report.

All available clinical information relevant to the evaluation of a related SAE, serious ADR or special situation is required.

- The Lead Principal Investigator is responsible for ensuring that these cases are complete and if not are promptly followed-up. A safety report is not considered complete until all clinical details needed to interpret the case are received. Reporting of follow-up information should follow the same timeline as initial reports.

- Copies of any and all relevant correspondences with regulatory authorities and ethics committees regarding any and all serious adverse events, irrespective of association with the Janssen Product under study, are to be provided to Janssen Scientific Affairs, LLC using a transmission method in Section 10 from this Exhibit within 24 hours of such report or correspondence being sent to applicable health authorities.

Non-Serious AEs

All non-serious adverse events should be reported to Janssen Scientific Affairs, LLC according to the timeframe outlined in the Research Funding Agreement section entitled Reporting of Data.

PQC Reporting

A PQC may have an impact on the safety and efficacy of the product. Timely, accurate, and complete reporting and analysis of PQC information from studies are crucial for the protection of patients, investigators, and Janssen Scientific Affairs, LLC, and are mandated by regulatory agencies worldwide. Janssen Scientific Affairs, LLC has established procedures in conformity with regulatory requirements worldwide to ensure appropriate reporting of PQC information. Lot and/or Batch #s shall be collected or any reports failure of expected pharmacological action (i.e., lack of effect). The product should be quarantined immediately and if possible, take a picture.

All initial PQCs involving a Janssen medicinal product under study must be reported to Janssen Scientific Affairs, LLC by the Lead Principal Investigator within 24 hours after being made aware of the event. The Janssen contact will provide additional information/form to be completed.

If the defect for a Janssen medicinal product under study is combined with either a serious adverse event or non-serious adverse event, the Lead Principal Investigator must report the PQC to Janssen Scientific Affairs, LLC according to the serious adverse event reporting timelines. A sample of the suspected product should be maintained for further investigation if requested by Janssen Scientific Affairs, LLC.

Reporting Procedures for Reporting Safety Data and Product Quality Complaints (PQCs) for Non-Janssen Medicinal Products

For SAEs, special reporting situations and PQCs following exposure to a non- Janssen medicinal product under study, the Lead Principal Investigator should notify the appropriate regulatory/competent authority or the manufacturer of that medicinal product (in the absence of appropriate local legislation) as soon as possible.

Transmission Methods

The following methods are acceptable for transmission of safety information to Janssen Scientific Affairs, LLC:

- Electronically via Janssen SECURE Email service (preferred)
- For business continuity purposes, if SECURE Email is non-functional
- Facsimile (fax), receipt of which is evidenced in a successful fax transmission report
- Telephone (if fax is non-functional)

Please use the contact information and process information provided by Janssen Scientific Affairs, LLC.

8.3.4 Pregnancy Reporting

Pregnancy of a female subject or the female partner of a male subject occurring while the subject is on treatment or within 4 weeks after the subject's last dose of study drug are considered expedited reportable events. Study drugs—lenalidomide, dexamethasone, carfilzomib, and daratumumab—are to be discontinued immediately. The pregnancy must be reported within 24 hours of the treating investigator's knowledge of the pregnancy by phone and facsimile using the SAE form to the University of Chicago CRA either by fax or by email. The treating investigator must inform the University of Chicago in writing by email or facsimile of any pregnancy within 24 hours / 1 business day at the latest on the following workday of being aware of the event. The University of Chicago must report pregnancy as an SAE directly to Amgen, Janssen, and Celgene using expedited reporting procedures listed in Section 8.3.3 and 8.3.3.2.

Pregnancy Reporting by Lead Principal Investigator to Celgene

For lenalidomide pregnancies and suspected pregnancies (including a positive pregnancy test regardless of age or disease state) of a female subject occurring while the subject is on lenalidomide, or within 28 days of the subject's last dose of lenalidomide, are considered immediately reportable events. Lenalidomide is to be discontinued immediately and the subject instructed to return any unused portion of the lenalidomide to the investigator. The pregnancy, suspected pregnancy, or positive pregnancy test must be reported to the Lead Principal Investigator who will inform Celgene immediately using the Pregnancy Reporting Form provided by Celgene or an approved equivalent form. The exposure of any pregnant female (e.g., caregiver or pharmacist) to lenalidomide is also an immediately reportable event.

The female subject should be referred to an obstetrician-gynecologist, preferably one experienced in reproductive toxicity for further evaluation and counseling.

The Lead Principal Investigator will follow the female subject until completion of the pregnancy, and must notify Celgene immediately about the outcome of the pregnancy (either normal or abnormal outcome).

If the outcome of the pregnancy was abnormal (e.g., spontaneous or therapeutic abortion), the Investigator should report the abnormal outcome as an AE. If the abnormal outcome meets any of the serious criteria, it must be reported as an SAE within 24 hours of the investigator's knowledge of the event using the SAE Report Form, or MedWatch Form.

All neonatal deaths that occur within 28 days of birth should be reported, without regard to causality, as SAEs. In addition, any infant death after 28 days that the Investigator suspects is related to the in utero exposure to the lenalidomide should also be reported within 24 hours of the investigator's knowledge of the event using the SAE Report Form, or approved equivalent form.

If a female partner of a male subject taking investigational product becomes pregnant, the male subject taking IP should notify the investigator, and the pregnant female partner should be advised to call their healthcare provider immediately.

If a pregnancy related event is reported in a female partner of a male subject, the investigator should ask if the female partner is willing to share information with Celgene Drug Safety and allow the pregnancy related event to be followed up to completion.

The Lead Principal Investigator will inform Celgene immediately, using the Pregnancy Reporting Form provided by Celgene or an approved equivalent form, of any information related to pregnancies or suspected pregnancies (including a positive pregnancy test regardless of age or disease state) occurring in partner of patients while the patients are still treated with lenalidomide or within 28 days of the Patients' last dose of lenalidomide.

Overdose

Overdose, as defined for this protocol, refers to lenalidomide, carfilzomib, daratumumab and dexamethasone dosing only.

On a per dose basis, an overdose is defined as the following amount over the protocol-specified dose of lenalidomide, carfilzomib, daratumumab and dexamethasone assigned to a given patient, regardless of any associated adverse events or sequelae.

- PO any amount over the protocol-specified dose
- IV 10% over the protocol-specified dose

On a schedule or frequency basis, an overdose is defined as anything more frequent than the protocol required schedule or frequency.

On an infusion rate basis, an overdose is defined as any rate faster than the protocol-specified rate. Complete data about drug administration, including any overdose, regardless of whether the overdose was accidental or intentional, should be reported in the case report form.

Pregnancy Reporting by Lead Principal Investigator to Amgen

Report Pregnancy and potential infant exposure including lactation, within ten (10) calendar days of the Lead Principal Investigator awareness. Provide to Amgen the SAE reports associated with pregnancy.

Subjects, spouses, or partners will be followed through the outcome of the pregnancy.

If the outcome of the pregnancy meets a criterion for immediate classification as an SAE—spontaneous abortion (any congenital anomaly detected in an aborted fetus is to be documented), stillbirth, neonatal death, or congenital anomaly—the treating investigator should repeat the procedures for expedited reporting of SAEs as outlined above.

Pregnancy Reporting by Lead Principal Investigator to Janssen

All initial reports of pregnancy must be reported to Janssen Scientific Affairs, LLC by the Principal Investigator within 24 hours of becoming aware of the event using the Serious Adverse Event Form. Abnormal pregnancy outcomes (e.g. spontaneous abortion, fetal death, stillbirth, congenital anomaly, ectopic pregnancy) are considered serious adverse events and must be reported using the Serious Adverse Event Form.

Because the effect of the Janssen medicinal product on sperm is unknown, pregnancies in partners of male subjects exposed to a Janssen medicinal product will be reported by the Lead Principal Investigator within

24 hours of their knowledge of the event using the Serious Adverse Event Form. Depending on local legislation this may require prior consent of the partner.

Follow-up information regarding the outcome of the pregnancy and any postnatal sequelae in the infant will be required.

9 INVESTIGATIONAL MEDICINAL PRODUCT

9.1 Carfilzomib

9.1.1 Description

Carfilzomib is a synthetic small molecule peptide bearing the chemical name (2S)-N-((S)-1-((S)-4-methyl-1-((R)-2-methyloxiran-2-yl)-1-oxopentan-2-ylcarbamoyl)-2-phenylethyl)-2-((S)-2-(2-morpholinoacetamido)-4-phenylbutanamido)-4-methylpentanamide. The molecular formula is $C_{40}H_{57}N_5O_7$ and the molecular weight is 719.91. It specifically functions as an inhibitor of the chymotrypsin-like activity of the 20S proteasome, which leads to the accumulation of protein substrates within the cell and induction of apoptosis.

9.1.2 Formulation

Carfilzomib for Injection will be provided as a lyophilized powder which, when reconstituted, contains 2 mg/mL isotonic solution of carfilzomib Free Base in 10 mM sodium citrate buffer (pH 3.5) containing 10% (w/v) sulfobutylether--cyclodextrin (SBE--CD, Captisol®).

9.1.3 Storage

Lyophilized Carfilzomib for Injection must be stored at 2–8ºC under the conditions outlined in the separate Pharmacy Manual, in a securely locked area to which access is limited to appropriate study personnel.

9.1.4 Accountability

Amgen, Inc. and the Site Investigator will maintain records of each shipment of investigational product. The records will document shipment dates, method of shipment, batch numbers, and quantity of vials contained in the shipment. Upon receipt of the investigational product, the designated recipient at the study site will inspect the shipment, verify the number and condition of the vials, and prepare an inventory or drug accountability record.

Drug accountability records must be readily available for inspection.

Empty and partially used vials should be accounted for and destroyed at the study site in accordance with the internal standard operating procedures. Drug destruction records must be readily available for inspection.

Only sites that cannot destroy unused drug on-site will be required to return their unused supply of investigational product.

9.2 Lenalidomide

9.2.1 Description

Lenalidomide, a thalidomide analogue, is an immunomodulatory agent with anti-angiogenic and anti-neoplastic properties. The chemical name is 3-(4-amino-1-oxo 1, 3-dihydro-2H-isoindol-2-yl) piperidine-2, 6-dione. The empirical formula for lenalidomide is C13H13N3O3, and the gram molecular weight is 259.3.

Lenalidomide is off-white to pale-yellow solid powder. It is soluble in organic solvent/ water mixtures, and buffered aqueous solvents. Lenalidomide is more soluble in organic solvents and low pH solutions. Solubility was significantly lower in less acidic buffers, ranging from about 0.4 to 0.5 mg/mL. Lenalidomide has an asymmetric carbon atom and can exist as the optically active forms S(-) and R(+), and is produced as a racemic mixture with a net optical rotation of zero. Lenalidomide is available in 5 mg, 10 mg, 15 mg and 25 mg capsules for PO administration. Each capsule contains lenalidomide as the active ingredient and the following inactive ingredients: lactose anhydrous, microcrystalline cellulose, croscarmellose sodium, and magnesium stearate. The 5 mg and 25 mg capsule shell contains gelatin, titanium dioxide and black ink. The 10 mg capsule shell contains gelatin, FD&C blue #2, yellow iron oxide, titanium dioxide and black ink. The 15 mg capsule shell contains gelatin, FD&C blue #2, titanium dioxide and black ink.

9.2.2 Supply

Lenalidomide (Revlimid) will come from commercially-available supply. Lenalidomide will be provided in accordance with Celgene Corporation's Revlimid REMS® program. Per standard Revlimid REMS® program requirements, all physicians who prescribe lenalidomide for research subjects enrolled into this

trial, and all research subjects enrolled into this trial, must be registered in, and must comply with, all requirements of the Revlimid REMS®program.

Lenalidomide will be shipped to the subjects' homes directly. If any unused drug is returned to the site by the subject it should be destroyed according to the local pharmacy's destruction policy

Further information about the Revlimid REMS® program is available at www.celgeneriskmanagement.com.

9.2.3 Storage Conditions

Store lenalidomide at 25°C (77 °F) away from direct sunlight; excursions permitted to 15-30°C (59-86 °F).

9.2.4 Accountability

Bottles of lenalidomide will contain a sufficient number of capsules to last for one cycle of dosing. Sites will be required to record and document subject compliance regarding lenalidomide dosing.

9.2.5 Prescribing Information

Lenalidomide will be provided in accordance with the Revlimid REMS® program of Celgene Corporation. Per standard Revlimid REMS® requirements, all physicians who prescribe lenalidomide for research subjects enrolled into this trial must be registered in must comply with all requirements of the Revlimid REMS® program. Prescriptions must be filled within 7 days for females of childbearing potential and 14 days for all other risk categories. Only enough lenalidomide for one cycle of therapy will be supplied to the patient each cycle.

9.2.6 Special Handling Instructions

Females of child-bearing potential should not handle or administer lenalidomide unless they are wearing gloves.

9.3 Dexamethasone

Dexamethasone may be given IV or PO

9.3.1 Description

Dexamethasone, a synthetic adrenocortical steroid, is a white to practically white, odorless, crystalline powder. It is stable in air. It is practically insoluble in water.

9.3.2 Formulation

Dexamethasone is a commercially available PO drug, supplied as 2 and 4 mg tablets.

9.3.3 Storage Conditions

Store dexamethasone at controlled room temperature 20 to 25ºC (68 to 77ºF)

9.3.4 Accountability

Sites will be required to record and document subject compliance regarding dexamethasone dosing.

9.4 Daratumumab Subcutaneous Injection

9.4.1 Description

Daratumumab-SC will be provided as a fixed-dose (1800 mg), combination drug product containing rHuPH20 drug substance (2000 U/mL) and daratumumab drug substance (120 mg/mL) in a single vial.

9.4.2 Supply

9.4.3 The daratumumab supplied for this study is manufactured by Janssen Biotech Inc. and provided under the responsibility of Janssen Scientific Affairs, LLC.Storage Conditions

Daratumumab must be stored in the original carton at controlled temperatures in a refrigerator ranging from 2°C to 8°C under the conditions outlined in the separate Pharmacy Manual. The product must be protected from direct sunlight and must not be frozen.

9.4.4 Accountability

The Site Investigator will maintain records of each shipment of investigational product. The records will document shipment dates, method of shipment, batch numbers, and quantity of vials contained in the shipment. Upon receipt of the investigational product, the designated recipient at the study site will inspect

the shipment, verify the number and condition of the vials, and prepare an inventory or drug accountability record.

Drug accountability records must be readily available for inspection.
Empty and partially used vials should be accounted for and destroyed at the study site in accordance with the internal standard operating procedures. Drug destruction records must be readily available for inspection.

10 STATISTICAL CONSIDERATIONS

10.1 Objectives

10.1.1 Primary Objective

The primary objective of this study is to evaluate the rate of sCR and the rate MRD-negative disease by NGS at the end of 8 cycles.

10.1.2 Secondary Objectives

Secondary objectives include characterization of efficacy variables:

- Rate of MRD by next generation gene sequencing (NGS) by clonoSEQ (Adaptive Biotechnologies) at the end of Cycle 8 and at the end of cycle 24, and then yearly for as long no PD up to 2 years
- Duration of response (DOR), progression free survival (PFS), time to progression (TTP), and overall survival (OS)
- Overall response rate defined as partial response or better (>PR) including the rate of VGPR or better (>VGPR) and near complete response or better (sCR/CR/nCR) across entire treatment in high risk and low risk patients at indicated time points and as best response.
- Safety and tolerability of combination of D-KRD.

10.1.3 Exploratory Objectives

The exploratory objectives of this study are to conduct GEP, proteomics, RNASeq, and gene sequencing studies on pro-treatment patient samples to evaluate the correlation between treatment outcome and pre-treatment patient profile.

10.2 Sample Size Justification & Analysis Plan

10.2.1 Sample Size Justification

A total of 45 non-transplant candidates and/or transplant candidates who agreed to defer transplant will be enrolled and recruited. The sample size is selected to allow for a collection of sufficient data for evaluation of sCR and MRD rates at the end of 8 cycles, which assumes that we may not have successful MRD evaluation in up to 25% of pts (primary end-point), and that up to 15% transplant candidates may change their mind and want to proceed to transplant, both based on our historical experience in our prior KRd studies and is based on historical rates of sCR from KRd study without ASCT (or deferred transplant) of 30% at the end of 8 cycles, and preliminary rates of sCR and MRD by NGS at the end of cycle 8 of 69% and 66%, respectively (both to be updated at ASH 2016). Therefore, if 40% or 30 (75 * 0.40) recruited subjects cannot proceed with the study, our sample size will result in approximately 40 subjects for the primary and secondary endpoint analyses.

10.2.2 Primary Endpoint Analysis

The primary objective of this study is to evaluate the rate of sCR and the rate MRD-negative disease by NGS at the end of 8 cycles. Time to sCR and MRD-negative response will be calculated from the date of first treatment on protocol. Subjects at the time of analysis not having either endpoint will be censored. Adherence to protocol and ability to follow patient in follow-up is expected to be excellent; however, if any patient revokes consent for the study not due to disease progression, they will be censored on their last clinical visit follow-up date. Likewise, patients in follow-up that are lost or that refuse or revoke study participation will be censored on their last clinical visit follow-up date.

Primary analysis will require 45 non-transplant candidates and/or transplant candidates who agreed to defer transplant to test the null hypothesis that the sCR and/or negative MRD status by NGS rate after 8 cycles is, ≤30% against the alternative that it is,>30% using an exact one-sided binomial test at the alpha=0.10 significance level. The null hypothesis will be rejected if 17 or more sCR's and/or MRD-negative's responses are observed (>42.5%). The sample size of n=45 response evaluable subjects provide a little over 85% power if the true sCR and/or negative MRD status rate by NGS is 50%. Based on this analysis, the study will provide preliminary estimate of probability of improvement of outcome of treatment with KRd w/o ASCT or with deferred ASCT for transplant candidates. In addition, we will conduct secondary analysis of sCR and/or MRD rates at the end of 8 cycles to test the null hypothesis that the sCR and/or negative MRD status by NGS rate after 8 cycles is, ≤30% against the alternative that

it is, >60% using an exact one-sided binomial test at the alpha=0.10 significance level. The sample size of n=45 response evaluable subjects provide a little over 99% power if the true sCR and/or negative MRD status rate by NGS is 60%. Based on this analysis, the study will provide preliminary estimate of probability of similar efficacy of D+KRd to KRd+ASCT. The sample size is selected to also allow for collection of sufficient data for toxicity and tolerability evaluation of the D-KRd regimen. The frequency of adverse events will be summarized by type, grade, and attribution to the study drugs.

10.2.3 Secondary Endpoint Analysis

The rate of MRD-negative disease by NGS will be reported at screening, 8, 12, 18, 24 months and then yearly until progression up to 5 years. Additionally, sites will be required to provide unstained pre-treatment slides to assess MRD by Adaptive. Comparison between D+KRd and historical KRd+ASCT rates will be made using a standard chi-square or Fisher's exact test. At the conclusion of the trial, with all subjects evaluated for PFS assessment at 5-years, the rate of MRD-negative status will be compared between D+KRd and historical KRd+ASCT data. This analysis will be conducted by contingency tables, or by adjusted logistic regression models, which will explore for any important confounders. Improvement of response during therapy measured at 6 and 12 months first treatment on protocol will be compared between D+KRd and historical KRd+ASCT data. Proportions of improving patients will be reported by treatment group, and comparisons of those proportions will be conducted using the chi-square or Fisher's exact tests. Overall survival will be calculated from date of first treatment on protocol until death from any cause and measured based upon the intention-to-treat paradigm, and will be compared between D+KRd and historical KRd+ASCT data using Kaplan and Meier survival models and the log-rank test statistic. In addition, all secondary endpoints will be tested for differences to historical controls using the one-sample log-rank test. The duration of MRD-negative disease will be calculated between the date of first occurrence of MRD-negative status, until such time as MRD-positive disease assessment or date of disease progression. Comparisons of duration of MRD-negative status may require the use of interval censoring methods. And finally, safety of the regimens will be assessed by comparing CTCAE Grade toxicity occurrence between D+KRd and historical KRd+ASCT data. Toxicity of Grade 2 and higher will be tabulated for each system organ class (SOC) and common individual toxicities occurring with SOCs reported. The maximum graded toxicity overall and within SOC will be reported per patient with the results summarized by treatment group and frequencies compared using chi-square tests or Fisher's exact tests as appropriate.

11 DATA REPORTING

Data reporting will be performed utilizing the eVelos electronic data capture system for all patients. The University of Chicago CRA will provide the applicable user registration information.

All required data must be recorded in the eVelos database within two weeks of the completion of each cycle. AEs and SAEs are to be entered in eVelos in real time. SAEs in the US are also to be recorded on the paper Serious Adverse Event Form within 24 hours of the site's knowledge of the event and sent via email (preferred) or fax to the University of Chicago (PhaseIICRA@medicine.bsd.uchicago.edu or qaccto@bsd.uchicago.edu; Fax: 773-702-4889).

All case report forms must be completed by designated study personnel. Each screened patient (signed informed consent) is to be entered into eVelos within 48 hours of patient registration. In addition to direct data entry, providing supporting documentation is required as per Patient Enrollment and Registration policies. Source records are original documents, data, and records (e.g., medical records, raw data collection forms, pharmacy dispensing records, recorded data from automated instruments, laboratory data) that are relevant to the clinical trial. **Each site will prepare and maintain adequate and accurate source documents.** These documents are designed to record all observations and other pertinent data for each subject enrolled in this clinical trial. Source records must be adequate to reconstruct all data transcribed onto the case report form.

12 REGULATORY OBLIGATIONS

12.1 Informed Consent

No investigator may involve a human being as a subject in research unless the investigator has obtained the legally effective informed consent of the subject or the subject's legally authorized representative. An investigator shall seek such consent only under circumstances that provide the prospective subject or the subject's legally authorized representative sufficient opportunity to consider whether or not to participate, and that minimize the possibility of coercion or undue influence. The information that is given to the subject or the representative shall be in a language understandable to the subject or representative.

The Lead PI will provide the Site Investigator with an informed consent form (ICF) developed by the Lead PI. Local and/or institutional requirements may require disclosure of additional information in the ICF. Any changes to this document must be submitted to the University of Chicago CCTO for approval, prior to submission to the participating site IRB. The IRB will review the ICF for approval. A copy of the

IRB approval form must be submitted to the University of Chicago CCTO prior to initiation of the study at the participating site.

Before implementing any study procedure, informed consent shall be documented by the use of a written consent form approved by the IRB. A copy of signed ICF will be given to the subject or subject's legally authorized representative. The original signed consent must be maintained by the Site Investigator and available for inspection by the designated Lead Principal Investigator representative at any time.

The consent form will include the following:

1. The nature and objectives, potential risks and benefits of the intended study.
2. The length of study and the likely follow-up required.
3. Alternatives to the proposed study. (This will include available standard and investigational therapies. In addition, patients will be offered an option of supportive care for therapeutic studies.)
4. The name of the investigator(s) responsible for the protocol.
5. The right of the participant to accept or refuse study interventions/interactions and to withdraw from participation at any time.

12.2 Compliance with Laws and Regulations

The study will be conducted in accordance with U.S. Food and Drug Administration (FDA) and International Conference on Harmonization (ICH) Guidelines for Good Clinical Practice (GCP), the Declaration of Helsinki, Health Canada, and Institutional Review Board (IRB) or Ethics Committee requirements.

This study must have the approval of a properly constituted IRB or Ethics Committee. Before the investigational drug is shipped to the Site Investigator, the Lead Principal Investigator or designee will provide Amgen, Janssen and Celgene with a copy of the IRB or Ethics Committee and URPL approval letter stating that the study protocol and any subsequent amendments and informed consent form have been reviewed and approved.

The Lead Principal Investigator and Site Investigator or designee will be responsible for obtaining annual IRB or Ethics Committee re-approval, respectively, throughout the duration of the study, when necessary.

The Lead Principal Investigator and Site Investigator are also responsible for notifying their IRB or Ethics Committee and URPL respectively of any significant adverse events that are serious and unexpected as per their policies.

Amgen will provide the Lead Principal Investigator with any expedited safety reports generated from any ongoing studies with carfilzomib, changes to the Investigator's Brochure, and any other safety information which changes the risk/benefit profile of carfilzomib during the conduct of the study, to allow him/her to fulfill his/her obligation for timely reporting to the IRB/ECs, participating sites, and other Investigators participating in the study and URPL, when necessary.

Celgene will provide the Lead Principal Investigator with any expedited safety reports generated from any ongoing studies with lenalidomide, changes to the Investigator's Brochure, and any other safety information which changes the risk/benefit profile of lenalidomide during the conduct of the study, to allow him/her to fulfill his/her obligation for timely reporting to the IRB/ECs, participating sites, and other Investigators participating in the study and URPL, when necessary.

Janssen will provide the Lead Principal Investigator with any expedited safety reports generated from any ongoing studies with daratumumab, changes to the Investigator's Brochure, and any other safety information which changes the risk/benefit profile of daratumumab during the conduct of the study, to allow him/her to fulfill his/her obligation for timely reporting to the IRB/ECs, participating sites, and other Investigators participating in the study and URPL, when necessary.

Upon completion of the trial, the Lead Principal Investigator must provide the IRB or Ethics Committee and URPL, Amgen and Celgene with a summary of the trial's outcome.

12.3 Subject Confidentiality

Subject medical information obtained as part of this study is confidential, and must not be disclosed to third parties, except as noted below. The subject may request in writing that medical information be given to his/her personal physician.

The investigator/institution will permit direct access to source data and documents by the FDA and/or other applicable regulatory authority. The access may consist of trial-related monitoring, audits, IRB or Ethics Committee reviews, and FDA/URPL/EMA inspections.

Release of research results should preserve the privacy of medical information and must be carried out in accordance with Department of Health and Human Services Standards for Privacy of Individually Identifiable Health Information, 45 CFR 164.508.

12.4 Multicenter Guidelines

Clinical studies coordinated by The University of Chicago must be conducted in accordance with the ethical principles that are consistent with Good Clinical Practices (GCP) and in compliance with other applicable regulatory requirements

The Study Lead PI/Coordinating Center are responsible for distributing all official protocols, amendments, and Unexpected Event Safety Reports to all participating institutions for submission to their applicable local IRBs/EC as required.

13 ADMINISTRATION AND LEGAL OBLIGATIONS

13.1 Institutional Review Board (IRB) Approval and Consent

Unless otherwise specified, each participating institution must obtain approval from a valid IRB/EC before enrolling patients on this study. It is expected that the IRB/EC will have the proper representation and function in accordance with valid mandated regulations. The IRB/EC should approve the consent form and protocol.

In obtaining and documenting informed consent, the treating investigator should comply with the applicable regulatory requirement(s), and should adhere to Good Clinical Practice (GCP) and to ethical principles that have their origin in the Declaration of Helsinki.

Before recruitment and enrollment onto this study, the patient will be given a full explanation of the study and will be given the opportunity to review the consent form. Each consent form must include all the relevant elements currently required by the FDA Regulations and local or state regulations. Once this essential information has been provided to the patient and the treating investigator is assured that the patient understands the implications of participating in the study, the patient will be asked to give consent to participate in the study by signing an IRB/EC-approved consent form.

Prior to a patient's participation in the trial, the written informed consent form should be signed and personally dated by the patient and by the person who conducted the informed consent discussion.

13.1.1 Annual IRB Renewals, Continuing Review and Final Reports

A continuing review of the protocol will be completed by the University of Chicago IRB and the participating institutions' IRB/ECs at least once a year for the duration of the study. The annual IRB/EC renewal approvals for participating institutions should be forwarded promptly to the University

of Chicago's Regulatory Manager. If the institution's IRB/EC requires a new version of the consent form with the annual renewal, the consent form should be included with the renewal letter.

Final Reports will be provided to IRBs/IECs and the Lead Principal Investigator within 1 year since the end of long term follow-up.

13.2 Required Documentation

Before the study can be initiated at any site, the following documentation must be provided to the Cancer Clinical Trials Office at the University of Chicago Comprehensive Cancer Center.

- A copy of the official IRB approval letter for the protocol and informed consent
- IRB membership list
- CVs and medical licensure for the Site Investigator and any sub-investigators who will be involved in the study.
- CAP and CLIA Laboratory certification numbers and institution lab normal values
- Investigational drug accountability standard operating procedures
- Additionally, before the study can be initiated at any site, the required executed research contract/subcontract must be on file with the University of Chicago.

13.3 Protocol Amendments and Study Termination

All protocol amendments will be implemented by the Lead PI and must receive IRB/IEC and RA, approval before implementation, except where necessary to eliminate an immediate hazard to subjects. Amendments should only be submitted to the IRB/IEC/RA after consideration of Amgen, Janssen and Celgene.

All modifications to the protocol, consent form, and/or questionnaires will be submitted to the University of Chicago IRB for review and approval. A list of the proposed modifications or amendments to the protocol and/or an explanation of the need of these modifications will be submitted, along with a revised protocol incorporating the modifications.

Only the Study Lead PI can authorize any modifications, amendments, or termination of the protocol.

13.3.1 Amendments To The Protocol At Participating Sites

Once a protocol amendment has been approved by the University of Chicago IRB, the Regulatory Manager will send the amended protocol and consent form (if applicable) to the affiliate institutions electronically. Upon receipt of the packet the affiliate institution is expected to do the following:

- The affiliate must reply to the email from the Regulatory Manager indicating that the amendment was received by the institution and that it will be submitted to the local IRB.
- The amendment should be submitted to the affiliate institution's IRB as soon as possible after receipt. The amendment **must** be IRB approved by the institution **within 3 months** from the date that it was received.
- **The University of Chicago version date and/or amendment number must appear on the affiliate consent form and on the affiliate IRB approval letter.** The version dates can be found on the footer of every page of the protocol and consent form. The amendment number can be found on the University of Chicago IRB amendment approval letter that is sent with the protocol/amendment mailing.
- The IRB approval for the amendment and the amended consent form (if amended consent is necessary) for the affiliate institution must be sent to the designated UC Regulatory Manager as soon as it is received.

13.4 Study Documentation and Archive

13.4.1 Source Documents

Source records are original documents, data, and records (e.g., medical records, raw data collection forms, pharmacy dispensing records, recorded data from automated instruments, laboratory data) that are relevant to the clinical trial. The Lead Principal Investigator will prepare and maintain adequate and accurate source documents in the two countries separately (i.e. the Lead PI and his team will be responsible for keeping adequate source documents. These documents are designed to record all observations and other pertinent data for each subject enrolled in this clinical trial. Source records must be adequate to reconstruct all data transcribed onto the case report forms.

13.4.2 Record Retention

Study documentation includes all CRFs, data correction forms or queries, source documents, Lead PI-Investigator correspondence, monitoring logs/letters, and regulatory documents (e.g., protocol and amendments, IRB correspondence and approval, signed patient consent forms).

Source documents include all recordings of observations or notations of clinical activities and all reports and records necessary for the evaluation and reconstruction of the clinical research study.

Government agency regulations and directives require that all study documentation pertaining to the conduct of a clinical trial must be retained by the Study Lead Principal Investigator. Study documents should be kept on file until three years after the completion and final study report of this investigational study or five years after a marketing application is approved for the drug for the indications for which it is being investigated, whichever is longer.

13.4.3 Case Report Form Completion

The data collected for this study will be entered into a secure database eCRF. The Lead PI will provide the applicable user registration information. Source documentation must be available to support the computerized patient record. Source records are original documents, data, and records (e.g., medical records, raw data collection forms, pharmacy dispensing records, recorded data from automated instruments, laboratory data) that are relevant to the clinical trial. Each site will prepare and maintain adequate and accurate source documents. These documents are designed to record all observations and other pertinent data for each subject enrolled in this clinical trial. Source records must be adequate to reconstruct all data transcribed onto the case report form. Upon registration, source documentation including demographics, screening labs, subject demographics, physician's notes for confirmation of concurrent conditions, and confirmation of disease status and treatment history. Additional information may be requested on a case-by case basis.

AEs are to be entered in real time. SAEs are to be entered in eCRF on the SAE reporting form within 24 hours of the site's knowledge of the event (in addition to the paper SAE form). All other data is to be entered within 5 days of source acquisition.

The University of Chicago CRA is responsible for training affiliate sites. This will be done over a teleconference. Archival of Records

According to 21 CFR 312.62I, the Lead Principal Investigator shall retain records required to be maintained under this part for a period of 5 years following the date a marketing application is approved for the drug for the indication for which it is being investigated. If no application is to be filed or if the application is not approved for such indication, it is suggested that the Lead Principal Investigator these

records until 15 years after the investigation is discontinued and the FDA or applicable regulatory authorities are notified.

The Lead Principal Investigator must retain protocols, amendments, IRB/IEC/URPL approvals, copies of the Form FDA, signed and dated consent forms, medical records, case report forms, drug accountability records, all correspondence, and any other documents pertaining to the conduct of the study.

13.5 Clinical Monitoring Procedures

Clinical studies coordinated by the Lead Principal Investigator must be conducted in accordance with the ethical principles that are consistent with Good Clinical Practices (GCP) and in compliance with other applicable regulatory requirements.

Specifically, University of Chicago will be responsible for monitoring all sites, monitoring will be conducted to verify the following:

- Adherence to the protocol
- Completeness and accuracy of study data and samples collected
- Compliance with regulations
- Submission of required source documents

When reviewing data collection procedures, the discussion will include identification, agreement and documentation of data items for which the eCRF will serve as the source document. The investigator and the head of the medical institution (where applicable) agrees to allow the monitor direct access to all relevant documents and to allocate their time and the time to their staff to monitor to discuss findings and any issues.

Sites will be required to participate in monitoring as described in the Monitoring Plan.). Sites will be alerted to schedule a monitoring visit, when required, and to request source documentation.

13.5.1 Obligations of Study Site Investigators

The Study Site Investigator is responsible for the conduct of the clinical trial at the site in accordance with Title 21 of the Code of Federal Regulations, valid local regulations and the Declaration of Helsinki. The Study Site Investigator is responsible for personally overseeing the treatment of all study patients. He/she must assure that all study site personnel, including sub-investigators and other study staff members, adhere

to the study protocol and all FDA/GCP/NCI/local regulations and guidelines regarding clinical trials both during and after study completion.

The Study Site Investigator at each institution or site will be responsible for assuring that all the required data will be collected and entered into the CRFs. If monitoring visits or audits are conducted, he/she must provide access to original records to permit verification of proper entry of data.

13.5.2 Protocol Deviations

Protocol deviations are to be documented using the Protocol Deviation Form and sent via email to [redacted]

Deviations that are considered major because they impact subject safety or alter the risk/benefit ratio, compromise the integrity of the study data, and/or affect subjects' willingness to participate in the study must be reported within 7 days. Please contact the University of Chicago CRA [redacted] if you have questions about how to report deviations. All major protocol deviations should also be reported to the local IRB/valid EC of record according to their policies and procedures.

13.6 Data Safety and Monitoring

The sites participating on this study will be remotely monitored by the designated University of Chicago Clinical Research Associate (CRA) in accordance with the University of Chicago, Section of Hematology/Oncology standard operating procedure titled Monitoring of Multi-Institutional Investigator Initiated Clinical Trials.

Prior to subject recruitment, and unless otherwise specified, any participating site will undergo a Site Initiation Teleconference to be conducted by the designated University of Chicago research team. The site's Principal Investigator and his or her study staff must attend the site initiation meeting.

Participating sites will also undergo a site close-out teleconference upon completion, termination or cancellation of a study to ensure fulfillment of study obligations during the conduct of the study, and to ensure that the Site Investigator is aware of his/her ongoing responsibilities.

Unless otherwise specified, this protocol will undergo weekly review at the multi-institutional data and safety monitoring teleconference as per procedures specified by the UC CCC NCI.

The conference will review:

- Enrollment rate relative to expectations, characteristics of participants
- Safety of study participants (Serious Adverse Event & Adverse Event reporting)
- Adherence to protocol (protocol deviations)
- Completeness, validity and integrity of study data
- Retention of study participants

13.6.1 Data Safety and Monitoring Board

An independent Data and Safety Monitoring Board (DSMB) composed of two medical oncologists and a biostatistician will be established to review the safety and efficacy data on average every 6 months. Reports will be generated and provided to the DSMB by the protocol statisticians. The reports will contain enrollment/ registration numbers, AEs, response data, and other efficacy data. After each meeting, the DSMB will produce a report with a recommendation for the steering committee.

13.6.2 DSMB Member Scope of Work

The Data and Safety Monitoring Board is an independent body composed of two medical oncologists and a biostatistician. Their main responsibilities are to review data and safety monitoring reports prepared by the Coordinating Center at periodic intervals. They will also monitor patient accrual, submit requests for additional analyses as deemed necessary, evaluate the performance of the participating clinical sites, evaluate the performance of the Coordinating Center, and provide recommendations to the steering committee regarding protocol modifications and whether the study should continue as planned. This DSMB will be provided with the toxicity data from the completed and published KRd trial (Jakubowiak et al, Blood 2012;120:1801-9), and they will review reports of adverse events to ensure patient safety as well as efficacy analyses to determine whether sufficient evidence has emerged that toxicity in the D-KRd trial exceeds historical toxicity in the KRd trial, which would prompt consideration of early termination of the trial. The DSMB will participate in conference calls and/or face-to-face meetings with the Lead Principal investigator and study statisticians approximately every six months. The DSMB may also call for additional meetings and data reviews if they feel it is necessary for the safety of patients or the progress of the study. The DSMB is expected to approve meeting minutes and maintain confidentiality of all study results.

13.6.3 Steering Committee

There will be a steering committee composed of the Lead Principal Investigator and the statisticians. This committee will meet monthly to discuss the progress of the trial. Additionally, the steering committee will make final decisions on the evolution of the trial according to its progress.

13.7 Quality Assurance & Auditing

University of Chicago will be responsible for quality assurance at all sites.

In addition to the clinical monitoring procedures, the University of Chicago Comprehensive Cancer Center will perform routine Quality Assurance Audits of Investigator-initiated clinical trials at the University of Chicago as described in the NCI-approved UC CCC DSM Plan. Audits provide assurance that trials are conducted and study data are collected, documented and reported in compliance with the protocol. Further, quality assurance audits ensure that study data are collected, documented and reported in compliance with Good Clinical Practices (GCP) Guidelines and regulatory requirements. The audit will review subjects enrolled at the University of Chicago in accordance with audit procedures specified in the UC CCC Data and Safety Monitoring plan. For institutions who are formal members of the Personalized Cancer Care Consortium (PCCC), the UC CCC will conduct on site quality assurance audits on average every two years during the enrollment and treatment phase of the study.

Auditing procedures for participating sites that are not full members of the PCCC must be specified and approved by the UC CCC Clinical Research Advisory Committee. In general, for sites that are not full members of the PCCC, auditing responsibility will be delegated to the participating center, with the annual audit report forwarded to the University of Chicago for review.

A regulatory authority (e.g. FDA, URPL) may also wish to conduct an inspection of the study, during its conduct or even after its completion. If an inspection has been requested by a regulatory authority, the Site Investigator must immediately inform the University of Chicago Cancer Clinical Trials Office and Regulatory Manager that such a request has been made.

REFERENCES

Alsina M, T. S., Vallone M, Molineaux C, Kunkel L, and Goy A (2007). Phase 1 Single Agent Antitumor Activity of Twice Weekly Consecutive Day Dosing of the Proteasome Inhibitor Carfilzomib (PR-171) in Hematologic Malignancies. In, (Blood (ASH Annual Meeting Abstracts)), p. 110:411.

Anderson KC, J. S., Jakubowiak AJ (2008). Phase II Study of Lenalidomide (Len), Bortezomib (Bz), and Dexamethasone (Dex) in Patients (pts) with Relapsed and Refractory Multiple Myeloma (MM). In, (J Clin Oncol), p. 26 (suppl); Abstract 8545.

Arastu-Kapur S, S. K., Parlati F, and Bennett M (Nov 2008). Non-Proteasomal Targets of Proteasome Inhibitors Bortezomib and Carfilzomib In, (Blood (ASH Annual Meeting Abstracts)), p. 112: 2657.

Attal, M., Lauwers-Cances, V., Marit, G., Caillot, D., Moreau, P., Facon, T., Stoppa, A. M., Hulin, C., Benboubker, L., Garderet, L*., et al.* (2012). Lenalidomide maintenance after stem-cell transplantation for multiple myeloma. N Engl J Med *366*, 1782-1791.

Benboubker, L., Dimopoulos, M. A., Dispenzieri, A., Catalano, J., Belch, A. R., Cavo, M., Pinto, A., Weisel, K., Ludwig, H., Bahlis, N*., et al.* (2014). Lenalidomide and dexamethasone in transplant-ineligible patients with myeloma. N Engl J Med *371*, 906-917.

Bross, P. F., Kane, R., Farrell, A. T., Abraham, S., Benson, K., Brower, M. E., Bradley, S., Gobburu, J. V., Goheer, A., Lee, S. L*., et al.* (2004). Approval summary for bortezomib for injection in the treatment of multiple myeloma. Clin Cancer Res *10*, 3954-3964.

Corral, L. G., Haslett, P. A., Muller, G. W., Chen, R., Wong, L. M., Ocampo, C. J., Patterson, R. T., Stirling, D. I., and Kaplan, G. (1999). Differential cytokine modulation and T cell activation by two distinct classes of thalidomide analogues that are potent inhibitors of TNF-alpha. J Immunol *163*, 380-386.

Davies, F. E., Raje, N., Hideshima, T., Lentzsch, S., Young, G., Tai, Y. T., Lin, B., Podar, K., Gupta, D., Chauhan, D*., et al.* (2001). Thalidomide and immunomodulatory derivatives augment natural killer cell cytotoxicity in multiple myeloma. Blood *98*, 210-216.

Demo, S. D., Kirk, C. J., Aujay, M. A., Buchholz, T. J., Dajee, M., Ho, M. N., Jiang, J., Laidig, G. J., Lewis, E. R., Parlati, F*., et al.* (2007). Antitumor activity of PR-171, a novel irreversible inhibitor of the proteasome. Cancer Res *67*, 6383-6391.

de Weers M, Tai YT, van der Veer MS, Bakker JM, Vink T, Jacobs DC, et al. Daratumumab, a novel therapeutic human CD38 monoclonal antibody, induces killing of multiple myeloma and other hematological tumors. J Immunol. 2011;186(3):1840-1848.

Dimopoulos, M., Spencer, A., Attal, M., Prince, H. M., Harousseau, J. L., Dmoszynska, A., San Miguel, J., Hellmann, A., Facon, T., Foa, R*., et al.* (2007). Lenalidomide plus dexamethasone for relapsed or refractory multiple myeloma. N Engl J Med *357*, 2123-2132.

Dimopoulos, M. A., Moreau, Philippe, Palumbo, Antonio, Joshua, Douglas E., Pour, Ludek, Hajek, Roman, Facon, Thierry, Ludwig, Heinz, Oriol, Albert, Goldschmidt, Hartmut, Rosinol, Laura, Straub, Jan, Suvorov, Aleksandr, Araujo, Carla, Pika, Tomas, Gaidano, Gianluca, Weisel, Katja, Goranova-Marinova, Vesselina, Gillenwater, Heidi H., Chng, Wee Joo (2015). Carfilzomib and dexamethasone (Kd) vs bortezomib and dexamethasone (Vd) in patients (pts) with relapsed multiple myeloma (RMM): Results from the phase III study ENDEAVOR. In, (J Clin Oncol 33), p. (suppl; abstr 8509).

Dimopoulos, M. A., Oriol, A., Nahi, H., San-Miguel, J., Bahlis, N. J., Usmani, S. Z., Rabin, N., Orlowski, R. Z., Komarnicki, M., Suzuki, K., *et al.* (2016). Daratumumab, Lenalidomide, and Dexamethasone for Multiple Myeloma. New England Journal of Medicine *375*, 1319-1331.

Dredge, K., Horsfall, R., Robinson, S. P., Zhang, L. H., Lu, L., Tang, Y., Shirley, M. A., Muller, G., Schafer, P., Stirling, D., *et al.* (2005). Orally administered lenalidomide (CC-5013) is anti-angiogenic in vivo and inhibits endothelial cell migration and Akt phosphorylation in vitro. Microvasc Res *69*, 56-63.

FDA (2003). Bortezomib Drug Details, http://www.accessdata.fda.gov/scripts/cder/drugsatfda/index.cfm?fuseaction=Search.DrugDetails. In.

Jagannath S, V. R., Stewart K, Somlo G, Jakubowiak AJ, Trudel S, Shwartz T, Siegel D, and Kunkel L,The Multiple Myeloma Research Consortium (MMRC) (2009). Final Results of PX-171-003-A0, Part 1 of an Open-label, Single-arm, Phase II Study of Carfilzomib (CFZ) in Patients (pts) with Relapsed and Refractory Multiple Myeloma (MM) In, (ClinOncol.), p. 27:15s (suppl. abstr 8504).

Jakubowiak, A. J., Dytfeld, D., Griffith, K. A., Lebovic, D., Vesole, D. H., Jagannath, S., Al-Zoubi, A., Anderson, T., Nordgren, B., Detweiler-Short, K., *et al.* (2012). A phase 1/2 study of carfilzomib in combination with lenalidomide and low-dose dexamethasone as a frontline treatment for multiple myeloma. Blood *120*, 1801-1809.

Jakubowiak, A. J., Dytfeld, Dominik , Griffith, Kent A., Jasielec, Jagoda, McDonnell, Kathryn, Lebovic, Daniel, Vesole, David H, Jagannath, Sundar, Chottiner, Elaine G., Anderson, Tara B., Detweiler-Short, Kristen, Stockerl-Goldstein, Keith , Ahmed, Asra Z., Jobkar, Terri L., Durecki, Diane E., Mietzel, Melissa A, Couriel, Daniel R., Vij, Ravi, Stefan Kaminski, Mark (2013). TREATMENT OUTCOME WITH THE COMBINATION OF CARFILZOMIB, LENALIDOMIDE, AND LOW-DOSE DEXAMETHASONE (CRD) FOR NEWLY DIAGNOSED MULTIPLE MYELOMA (NDMM) AFTER EXTENDED FOLLOW-UP. In, (J Clin Oncol), pp. 31, 2013 (suppl; abstr 8543).

J & J Research & Development, L. (2016). An Open-label, Multicenter, Phase 1b Study of JNJ-54767414 (HuMax® CD38) (Anti-CD38 Monoclonal Antibody) in Combination with Backbone Regimens for the Treatment of Subjects with Multiple Myeloma Amendment INT-8. In. https://clinicaltrials.gov/ct2/show/NCT01998971

Kirk CJ, J. J., Muchamuel T, Dajee M, Swinarski D, Aujay M, Bennett MK, Yang J, Lewis E, Laidig G, and Molineaux CJ (Nov 2008). The Selective Proteasome Inhibitor Carfilzomib is Well Tolerated in Experimental Animals with Dose Intensive Administration. In, (Blood (ASH Annual Meeting Abstracts)), p. 112: 2765.

Kuhn, D. J., Chen, Q., Voorhees, P. M., Strader, J. S., Shenk, K. D., Sun, C. M., Demo, S. D., Bennett, M. K., van Leeuwen, F. W., Chanan-Khan, A. A., and Orlowski, R. Z. (2007). Potent activity of carfilzomib, a novel, irreversible inhibitor of the ubiquitin-proteasome pathway, against preclinical models of multiple myeloma. Blood *110*, 3281-3290.

Kumar, S. K., Rajkumar, S. V., Dispenzieri, A., Lacy, M. Q., Hayman, S. R., Buadi, F. K., Zeldenrust, S. R., Dingli, D., Russell, S. J., Lust, J. A., *et al.* (2008). Improved survival in multiple myeloma and the impact of novel therapies. Blood *111*, 2516-2520.

McCarthy, P. L., and Hahn, T. (2013). Strategies for induction, autologous hematopoietic stem cell transplantation, consolidation, and maintenance for transplantation-eligible multiple myeloma patients. Hematology Am Soc Hematol Educ Program *2013*, 496-503.

McCarthy, P. L., Owzar, K., Hofmeister, C. C., Hurd, D. D., Hassoun, H., Richardson, P. G., Giralt, S., Stadtmauer, E. A., Weisdorf, D. J., Vij, R., *et al.* (2012). Lenalidomide after stem-cell transplantation for multiple myeloma. N Engl J Med *366*, 1770-1781.

Niesvizky, e. a. (April 15, 2013). Phase 1b Dose-Escalation Study (PX-101-006) of Carfilzomib, Lenalidomide, and Low-Dose Dexamethasone in Relapsed or Progressive MM. . In, (Clin Cancer Res), pp. 19(18): 2248-2256.

Nooka, A. K., Kaufman, J. L., Muppidi, S., Langston, A., Heffner, L. T., Gleason, C., Casbourne, D., Saxe, D., Boise, L. H., and Lonial, S. (2014). Consolidation and maintenance therapy with lenalidomide, bortezomib and dexamethasone (RVD) in high-risk myeloma patients. Leukemia *28*, 690-693.

Overdijk MB, Verploegen S, Bogels M, et al. Phagocytosis is a potent mechanism of action for the therapeutic human monoclonal antibody daratumumab in lymphoma and multiple myeloma. Submitted to Blood. 13 May 2013.

Paiva, B., Vidriales, M. B., Cervero, J., Mateo, G., Perez, J. J., Montalban, M. A., Sureda, A., Montejano, L., Gutierrez, N. C., Garcia de Coca, A., *et al.* (2008). Multiparameter flow cytometric remission is the most relevant prognostic factor for multiple myeloma patients who undergo autologous stem cell transplantation. Blood *112*, 4017-4023.

Palumbo, A., Bringhen, S., Kumar, S. K., Lupparelli, G., Usmani, S., Waage, A., Larocca, A., van der Holt, B., Musto, P., Offidani, M., *et al.* (2014). Second primary malignancies with lenalidomide therapy for newly diagnosed myeloma: a meta-analysis of individual patient data. Lancet Oncol *15*, 333-342.

Palumbo, A., Chanan-Khan, A., Weisel, K., Nooka, A. K., Masszi, T., Beksac, M., Spicka, I., Hungria, V., Munder, M., Mateos, M. V., *et al.* (2016). Daratumumab, Bortezomib, and Dexamethasone for Multiple Myeloma. New England Journal of Medicine *375*, 754-766.

Rajkumar P, J. S., Taje N, et al. (2007). Phase III Trial of Lenalidomide Plus High-Dose Dexamethasone versus Lenalidomide Plus low-Dose Dexamethasone in Newly Diagnosed Multiple Myeloma (E4A03): A Trial Coordinated by the Eastern Cooperative Oncology Group. In, (J Clin Oncol), p. 25 (18S): Abstract LBA 8025.

Rajkumar, S. V., Hayman, S. R., Lacy, M. Q., Dispenzieri, A., Geyer, S. M., Kabat, B., Zeldenrust, S. R., Kumar, S., Greipp, P. R., Fonseca, R., *et al.* (2005). Combination therapy with lenalidomide plus dexamethasone (Rev/Dex) for newly diagnosed myeloma. Blood *106*, 4050-4053.

Richardson P, J. S., Jakubowiak AJ (Dec 6, 2008). Lenalidomide, Bortezomib, and Dexamethasone in Patients with Relapsed or Relapsed/Refractory Multiple Myeloma (MM): Encouraging Response Rates and Tolerability with Correlation of Outcome and Adverse Cytogenetics in a Phase II Study. In, (American Hematology Society Congress # 1742, San Francisco, CA, USA).

Richardson P, J. S., Raje N, et al. (2007). Lenalidomide, Bortezomib, and Dexamethasone (Rev/Vel/Dex) as Front-Line Therapy for patients with Multiple Myeloma (MM): Preliminary Results of a Phase 1/2 Study. In, (Blood), p. 110 (111): Abstract 187.

Richardson, P. G., Weller, E., Lonial, S., Jakubowiak, A. J., Jagannath, S., Raje, N. S., Avigan, D. E., Xie, W., Ghobrial, I. M., Schlossman, R. L., *et al.* (2010). Lenalidomide, bortezomib, and dexamethasone combination therapy in patients with newly diagnosed multiple myeloma. Blood *116*, 679-686.

Ries LAG, M. D., Krapcho M, et al (eds.) (2007). SEER Cancer Statistics Review, 1975-2004, National Cancer Institute. Bethesda, MD, http://seer.cancer.gov/csr/1975-2004/, based on November 2006 SEER data submission, posted to the SEER website, . In.

Rosinol, L., Oriol, A., Teruel, A. I., Hernandez, D., Lopez-Jimenez, J., de la Rubia, J., Granell, M., Besalduch, J., Palomera, L., Gonzalez, Y., *et al.* (2012). Superiority of bortezomib, thalidomide, and dexamethasone (VTD) as induction pretransplantation therapy in multiple myeloma: a randomized phase 3 PETHEMA/GEM study. Blood *120*, 1589-1596.

Schafer, P. H., Gandhi, A. K., Loveland, M. A., Chen, R. S., Man, H. W., Schnetkamp, P. P., Wolbring, G., Govinda, S., Corral, L. G., Payvandi, F., *et al.* (2003). Enhancement of cytokine production and AP-1 transcriptional activity in T cells by thalidomide-related immunomodulatory drugs. J Pharmacol Exp Ther *305*, 1222-1232.

Siegel D, W. L., Orlowski RZ, Kaufman JL, Stewart AK, Kukreti V, Alsina M, Jakubowiak AJ, Jagannath D, McDonagh KT, Belch A, Bahlis NJ, Shustik C, Le MH, Kunkel L, Bennett MK, Kauffman M, Vij R, and the Multiple Myeloma Research Consortium (MMRC) (Nov 2009). PX-171-004, An Ongoing Open-Label, Phase II Study of Single-Agent Carfilzomib (CFZ) in Patients with Relapsed of Refractory Myeloma (MM); Updated Results From the Bortezomib-Treated Cohort In, (Blood (ASH Annual Meeting Abstracts)), p. 114: 3877.

Siegel, D. S., Martin, T., Wang, M., Vij, R., Jakubowiak, A. J., Lonial, S., Trudel, S., Kukreti, V., Bahlis, N., Alsina, M., *et al.* (2012). A phase 2 study of single-agent carfilzomib (PX-171-003-A1) in patients with relapsed and refractory multiple myeloma. Blood *120*, 2817-2825.

Sonneveld, P., Schmidt-Wolf, I. G., van der Holt, B., El Jarari, L., Bertsch, U., Salwender, H., Zweegman, S., Vellenga, E., Broyl, A., Blau, I. W., *et al.* (2012). Bortezomib induction and maintenance treatment in patients with newly diagnosed multiple myeloma: results of the randomized phase III HOVON-65/ GMMG-HD4 trial. J Clin Oncol *30*, 2946-2955.

Spencer, A., Prince, H. M., Roberts, A. W., Prosser, I. W., Bradstock, K. F., Coyle, L., Gill, D. S., Horvath, N., Reynolds, J., and Kennedy, N. (2009). Consolidation therapy with low-dose thalidomide and prednisolone prolongs the survival of multiple myeloma patients undergoing a single autologous stem-cell transplantation procedure. J Clin Oncol *27*, 1788-1793.

Stewart, A. K., Rajkumar, S. V., Dimopoulos, M. A., Masszi, T., Spicka, I., Oriol, A., Hajek, R., Rosinol, L., Siegel, D. S., Mihaylov, G. G., *et al.* (2015). Carfilzomib, lenalidomide, and dexamethasone for relapsed multiple myeloma. N Engl J Med *372*, 142-152.

Stewart, A. K., Trudel, S., Bahlis, N. J., White, D., Sabry, W., Belch, A., Reiman, T., Roy, J., Shustik, C., Kovacs, M. J.*, et al.* (2013). A randomized phase 3 trial of thalidomide and prednisone as maintenance therapy after ASCT in patients with MM with a quality-of-life assessment: the National Cancer Institute of Canada Clinicals Trials Group Myeloma 10 Trial. Blood *121*, 1517-1523.

Wang (Oct 31, 2013). Phase 2 Dose-Explansion Study (PX-171-006) of Carfilzomib, Lenalidomide, and Low-Dose Dex in Relapsed or Progressive MM. In, (Blood), pp. Vol 122 no 118: 3122-3128.

Wang L, S. D., Kaufman JL, Stewart AK, Jakubowiak A, Alsina M, Kukreti V, Bahlis NJ, McDonagh KT, Belch A, Sebag M, Gabrail N, Le MH, Bennett MK, Kunkel L, Kauffman M, Orlowski RZ, Vij R, and The Multiple Myeloma Research Consortium (MMRC) (2009). Updated Results of Bortezomib-Naïve Patients in PX-171-004, An Ongoing Open-Label, Phase II Study of Single-Agent Carfilzomib (CFZ) in Patients with Relapsed or Regractory Myeloma (MM) In, (Blood (ASH Annual Meeting Abstracts)), p. 114: 302.

Weber, D. M., Chen, C., Niesvizky, R., Wang, M., Belch, A., Stadtmauer, E. A., Siegel, D., Borrello, I., Rajkumar, S. V., Chanan-Khan, A. A.*, et al.* (2007). Lenalidomide plus dexamethasone for relapsed multiple myeloma in North America. N Engl J Med *357*, 2133-2142.

Zimmerman, T. M., Griffith, Kent A., Jasielec, Jagoda, Rosenbaum, Cara A., McDonnell, Kathryn, Waite-Marin, Jessica, Berdeja, Jesus G., Raje, Noopur S., Reece, Donna E., Vij, Ravi, Alonge, Mattina, Rosebeck, Shaun, Gurbuxani, Sandeep, Faham, Malek, Kong, Katherine A., Levy, Joan, Jakubowiak, Andrzej J. (2015). Phase II MMRC trial of extended treatment with carfilzomib (CFZ), lenalidomide (LEN), and dexamethasone (DEX) plus autologous stem cell transplantation (ASCT) in newly diagnosed multiple myeloma (NDMM). In, (J Clin Oncol 33,), p. (suppl; abstr 8510).

Zimmerman, T. M., Griffith, Kent, Jasielec, Jagoda K. , Rosenbaum, Cara A. McDonnell, Kathryn, Waite-Marin, Jessica, Anastazi, John, Berdeja, Jesus G., Vij, Ravi, Jakubowiak, Andrzej J. (2014). INITIAL RESULTS FROM A PHASE 2 STUDY OF CARFILZOMIB, LENALIDOMIDE, AND LOW-DOSE DEXAMETHASONE (KRD) PLUS AUTOLOGOUS STEM CELL TRANSPLANTATION IN NEWLY DIAGNOSED MULTIPLE MYELOMA. In, (EHA (Abstract: P347)).

APPENDIX 1: MULTIPLE MYELOMA STAGING

A. Durie-Salmon Staging

Stage I

All of the following must be present:

- Hemoglobin > 10.5 g/dL or hematocrit >32%
- Serum calcium level normal (≤12 mg/dL)
- Low serum myeloma protein production rates as evidenced by all of the following:
  - IgG peak < 5g/dL
  - IgA peak < 3g/dL
  - Bence Jones protein < 4g/24 h
- No bone lesions

Stage II

All patients who do not meet criteria for Stage I or III are considered Stage II.

Stage III

One of the following abnormalities must be present:

- Hemoglobin < 8.5 g/dL, hematocrit < 25%
- Serum calcium >12 mg/dL
- Very high serum or urine myeloma protein production rates as evidenced by one or more of the following:
  - IgG peak > 7g/dL
  - IgA peak > 5g/dL
  - Bence Jones protein > 12g/24 h
  - > 3 lytic bone lesion on bone survey (bone scan not acceptable)

Sub-classification

a. Serum creatinine <2.0 mg/dL
b. Serum creatinine >2.0 mg/dL

B. International Myeloma Working Group International Staging System (ISS)

1. Stage I: B2M < 3.5 plus serum albumin ≥3.5 (med S 62m)
2. Stage II: B2M < 3.5 but serum alb. < 3.5 OR B2M 3.5 - < 5.5 (med S 44m)
3. Stage III: B2M ≥ 5.5 (med S 29m)
4. Sub-classify stages 1+2 according to cr< or ≥ 2 and stage 3 according to low platelets (< 130k) or high LDH

IMWG criteria for symptomatic myeloma:
All three criteria must be met:

1. Clonal bone marrow plasma cells and/or documented clonal plasmacytoma
2. Presence of serum and/or urinary monoclonal protein
3. Evidence of end-organ damage that can be attributed to the underlying plasma cell proliferative disorder, specifically:

a. Hypercalcemia: serum calcium ≥11.5 mg/dL or
b. Renal insufficiency: serum creatinine >2mg/dL or
c. Anemia: hemoglobin at least 2 g/dL below the lower limit of normal or a hemoglobin <10 g/dL or
Bone lesions: lytic lesions, osteopenia or pathologic fractures

APPENDIX 2: ECOG PERFORMANCE STATUS

Grade	Description
0	Normal activity, fully active, able to carry on all pre-disease performance without restriction.
1	Symptoms, but fully ambulatory, restricted in physically strenuous but ambulatory and able to carry out work of a light or sedentary nature (e.g., light housework, office work).
2	Ambulatory and capable of all self-care but unable to carry out any work activities. Up and about more than 50% of waking hours.
3	Capable of only limited self-care, confined to bed or chair more than 50% of waking hours.
4	Completely disabled. Cannot carry on any self-care. Totally confined to bed or chair.
5	Dead

APPENDIX 3: NCI CTCAE VERSION 4.0

Common Terminology Criteria for Adverse Events (CTCAE) of the National Cancer Institute (NCI) v4.0

Publish Date: September 15, 2009

http://evs.nci.nih.gov/ftp1/CTCAE/CTCAE_4.03_2010-06-14_QuickReference_5x7.pdfhttp://evs.nci.nih.gov/ftp1/CTCAE/CTCAE_4.03_2010-06-14_QuickReference_5x7.pdf

http://evs.nci.nih.gov/ftp1/CTCAE/CTCAE_4.03_2010-06-14_QuickReference_5x7.pdf

APPENDIX 4: RESPONSE CRITERIA FOR MULTIPLE MYELOMA

IMWG Criteria

Response	***IMWG criteria***[1,2]
sCR<br><br>Stringent Complete Response	CR as defined below plus:<br>normal FLC ratio and<br>absence of clonal cells in bone marrow by immunohistochemistry or 2 – 4 color flow cytometry
CR<br><br>Complete Response	Negative immunofixation on the serum and urine and<br>disappearance of any soft tissue plasmacytomas and<br>< 5% plasma cells in bone marrow.<br>In patients with only FLC disease, a normal FLC ratio of 0.26–1.65 is required.
VGPR<br><br>Very Good Partial Response	Serum and urine M-protein detectable by immunofixation but not on electrophoresis or<br>≥ 90% reduction in serum M-protein plus urine M-protein level < 100 mg/24 h.<br>In patients with only FLC disease, >90% decrease in the difference between involved and uninvolved FLC levels is required.
PR<br><br>Partial Response	50% reduction of serum M-protein and reduction in 24 hours urinary M-protein by ≥90% or to < 200 mg/24 h<br>If the serum and urine M-protein are unmeasurable,[3] a ≥ 50% decrease in the difference between involved and uninvolved FLC levels is required in place of the M-protein criteria<br>If serum and urine M-protein are not measurable, and serum free light assay is also not measurable, ≥ 50% reduction in plasma cells is required in place of M-protein, provided baseline bone marrow plasma cell percentage was ≥ 30%<br>In addition to the above listed criteria, if present at baseline, a ≥ 50% reduction in the size of soft tissue plasmacytomas is also required
Stable Disease	Not meeting criteria for CR, VGPR, PR or progressive disease

Progressive disease	Increase of ≥ 25% from lowest response value in any one of the following:<br>Serum M-component (the absolute increase must be ≥ 0.5 g/dL)[4]and/or<br>Urine M-component (the absolute increase must be ≥ 200 mg/24 h)and/or<br>Only in patients without measurable serum and urine M-protein, the difference between involved and uninvolved FLC levels. The absolute increase must be > 10 mg/dL<br>Only in patients without measurable serum and urine M-protein and without measurable disease by FLC levels, bone marrow plasma cell percentage (absolute % must be ≥ 10%)<br>Definite development of new bone lesions or soft tissue plasmacytomas or definite increase in the size of existing bone lesions or soft tissue plasmacytomas<br>Development of hypercalcemia (corrected serum calcium >11.5 mg/dL) that can be attributed solely to the plasma cell proliferative disorder

All relapse categories (CR, sCR, VGPR, and PD) require two consecutive assessments made at any time before the institution of any new therapy; complete response and PR and SD categories also require no known evidence of progressive or new bone lesions if radiographic studies were performed. VGPR and CR categories require serum and urine studies regardless of whether disease at baseline was measurable in serum, urine both or either. Radiographic studies are not required to satisfy these response requirements. Bone marrow assessments need not be confirmed. For progressive disease, serum M-component increases of ≥ I gm/dl are sufficient to define response if starting M-component is ≥ 5 g/dl.

IMWG clarification for coding PD:

- Clarified that bone marrow criteria for PD are to be used only in patients without measurable disease by M protein and by FLC levels.
- Clarified that 25% increase refers to M protein, FLC, and bone marrow results and does not refer to bone lesions, soft tissue plasmacytomas or hypercalcemia. Note the lowest response value does not need to be a confirmed value.

Additional response criteria for specific disease states[1,2,3,4]

Minor response in patients with relapsed and refractory myeloma adapted from the EMBT criteria3	≥ 25% but < 49% reduction of serum M protein and reduction in 24-hour urine M protein by 50–89%, which still exceeds 200 mg/24hrs.<br>In addition to above; if present at baseline, 25-49% reduction in the size of soft tissue plasmacytomas is also required<br>No increase in size or number of lytic bone lesions (development of compression fractures does not exclude response)
Near Complete Response nCR	The absence of myeloma protein on electrophoresis, with positive immunofixation, stable bone disease, and a normal serum calcium concentration
Immunophenotypic CR	Stringent CR plus<br>Absence of phenotypic aberrant PC (clonal) in bone marrow with a minimum of one million of total BM

	cells analyzed by multiparametric flow cytometry (with ≥4 colors)
Molecular CR	Stringent CR plus negative ASO-PCR (sensitivity 10^{-5})

1. Durie et al. International uniform response criteria for multiple myeloma. Leukemia 2006;20:1467-73
2. S. Vincent Rajkumar, Jean-Luc Harousseau, Brian Durie, Kenneth C. Anderson, Meletios Dimopoulos, Robert Kyle, Joan Blade, Paul Richardson, Robert Orlowski, David Siegel, Sundar Jagannath, Thierry Facon, Hervé Avet-Loiseau, Sagar Lonial, Antonio Palumbo, Jeffrey Zonder, Heinz Ludwig, David Vesole, Orhan Sezer, Nikhil C. Munshi, and Jesus San Miguel. Consensus recommendations for the uniform reporting of clinical trials: report of the International Myeloma Workshop Consensus Panel 1 Blood First Edition Paper, prepublished online February 3, 2011;DOI 10.1182/blood-2010-10-299487
3. Richardson PG, Barlogie B, Berenson J, Singhal S, Jagannath S, Irwin D, Rajkumar SV, Srkalovic G, Alsina M, Alexanian R, Siegel D, Orlowski RZ, Kuter D, Limentani SA, Lee S, Hideshima T, Esseltine DL, Kauffman M, Adams J, Schenkein DP, Anderson KC. A Phase 2 study of bortezomib in relapsed, refractory myeloma. N Engl J Med 348:2609, June 2, 2003.
4. Richardson et al. Bortezomib or High-Dose Dexamethasone for Relapsed Multiple Myeloma. N Eng J Med. 352:2487-98, 2005

APPENDIX 5: FACT/GOG-NEUROTOXICITY QUESTIONNAIRE, V. 4.0

By circling one (1) number per line, please indicate how true each statement has been for you during the past 7 days.

**ADDITIONAL CONCERNS**	**Not at all**	**A little bit**	**Some-what**	**Quite a bit**	**Very much**
I have numbness or tingling in my hands………	0	1	2	3	4
I have numbness or tingling in my feet………..	0	1	2	3	4
I feel discomfort in my hands………………….	0	1	2	3	4
I feel discomfort in my feet……………………	0	1	2	3	4
I have joint pain or muscle cramps……………	0	1	2	3	4
I feel weak all over……………………………	0	1	2	3	4
I have trouble hearing…………………………	0	1	2	3	4
I get a ringing or buzzing in my ears………..	0	1	2	3	4
I have trouble buttoning buttons………………	0	1	2	3	4
I have trouble feeling the shape of small objects when they are in my hand………………………	0	1	2	3	4
I have trouble walking…………………………	0	1	2	3	4

Sources: Cella DF, Tulsky DS, Gray G, Sarafian B, Lloyd S, Linn E, et al. The functional assessment of cancer therapy (FACT) scale: development and validation of the general measure. *J ClinOncol* 1993;11(3):570-79.

APPENDIX 6: QUALITY OF LIFE ASSESSMENT TOOL

EORTC QLQ-C30 (version 3)

We are interested in some things about you and your health. Please answer all of the questions yourself by circling the number that best applies to you. There are no "right" or "wrong" answers. The information that you provide will remain strictly confidential.

Please fill in your initials:
Your birthdate (Day, Month, Year):
Today's date (Day, Month, Year):

	Not at All	A Little	Quite a Bit	Very Much
1. Do you have any trouble doing strenuous activities, like carrying a heavy shopping bag or a suitcase?	1	2	3	4
2. Do you have any trouble taking a <u>long</u> walk?	1	2	3	4
3. Do you have any trouble taking a <u>short</u> walk outside of the house?	1	2	3	4
4. Do you need to stay in bed or a chair during the day?	1	2	3	4
5. Do you need help with eating, dressing, washing yourself or using the toilet?	1	2	3	4

During the past week:

	Not at All	A Little	Quite a Bit	Very Much
6. Were you limited in doing either your work or other daily activities?	1	2	3	4
7. Were you limited in pursuing your hobbies or other leisure time activities?	1	2	3	4
8. Were you short of breath?	1	2	3	4
9. Have you had pain?	1	2	3	4
10. Did you need to rest?	1	2	3	4
11. Have you had trouble sleeping?	1	2	3	4
12. Have you felt weak?	1	2	3	4
13. Have you lacked appetite?	1	2	3	4

14.	Have you felt nauseated?	1	2	3	4
15.	Have you vomited?	1	2	3	4
16.	Have you been constipated?	1	2	3	4

Please go on to the next page

**During the past week:**	**Not at All**	**A Little**	**Quite a Bit**	**Very Much**
17. Have you had diarrhea?	1	2	3	4
18. Were you tired?	1	2	3	4
19. Did pain interfere with your daily activities?	1	2	3	4
20. Have you had difficulty in concentrating on things, like reading a newspaper or watching television?	1	2	3	4
21. Did you feel tense?	1	2	3	4
22. Did you worry?	1	2	3	4
23. Did you feel irritable?	1	2	3	4
24. Did you feel depressed?	1	2	3	4
25. Have you had difficulty remembering things?	1	2	3	4
26. Has your physical condition or medical treatment interfered with your family life?	1	2	3	4
27. Has your physical condition or medical treatment interfered with your social activities?	1	2	3	4
28. Has your physical condition or medical treatment caused you financial difficulties?	1	2	3	4

For the following questions please circle the number between 1 and 7 that best applies to you

29. How would you rate your overall health during the past week?

1 2 3 4 5 6 7

Very poor Excellent

30. How would you rate your overall quality of life during the past week?

1 2 3 4 5 6 7

Very poor Excellent

EORTC QLQ – MY20

Patients sometimes report that they have the following symptoms or problems. Please indicate the extent to which you have experienced these symptoms or problems <u>during the past week</u>. Please answer by circling the number that best applies to you.

**During the past week:**		**Not at All**	**A Little**	**Quite a Bit**	**Very Much**
31.	Have you had bone aches or pain?	1	2	3	4
32.	Have you had pain in your back?	1	2	3	4
33.	Have you had pain in your hip?	1	2	3	4
34.	Have you had pain in your arm or shoulder?	1	2	3	4
35.	Have you had pain in your chest?	1	2	3	4
36.	If you had pain did it increase with activity?	1	2	3	4
37.	Did you feel drowsy?	1	2	3	4
38.	Did you feel thirsty?	1	2	3	4
39.	Have you felt ill?	1	2	3	4
40.	Have you had a dry mouth?	1	2	3	4
41.	Have you lost any hair?	1	2	3	4
42.	Answer this question only if you lost any hair: Were you upset by the loss of your hair?	1	2	3	4
43.	Did you have tingling hands or feet?	1	2	3	4
44.	Did you feel restless or agitated?	1	2	3	4
45.	Have you had acid indigestion or heartburn?	1	2	3	4
46.	Have you had burning or sore eyes?	1	2	3	4

Please turn to next page

**During the past week:**	**Not at All**	**A Little**	**Quite a Bit**	**Very Much**
47. Have you felt physically less attractive as a result of your disease or treatment?	1	2	3	4
48. Have you been thinking about your illness?	1	2	3	4
49. Have you been worried about dying?	1	2	3	4
50. Have you worried about your health in the future?	1	2	3	4

© Copyright 1999 EORTC Study Group on Quality of Life. All rights reserved.

APPENDIX 7: Lenalidomide Pregnancy Risk Minimization Plan for Celgene Clinical Trials

Lenalidomide Pregnancy Prevention Plan for Subjects in Clinical Trials

The Pregnancy Prevention Plan (PPP) applies to all subjects receiving lenalidomide within a clinical trial. The following PPP documents are included:

1. The Lenalidomide Risks of Fetal Exposure, Pregnancy Testing Guidelines and Acceptable Birth Control Methods document (Section 0) provides the following information:

- Potential risks to the fetus associated with lenalidomide exposure
- Definition of female of childbearing potential (FCBP)/female not of childbearing potential (FNCBP)
- Requirements for counseling of all subjects receiving lenalidomide about pregnancy precautions and the potential risks of fetal exposure to lenalidomide
- Acceptable birth control methods for both female subjects of childbearing potential and male subjects receiving lenalidomide in the study
- Pregnancy testing requirements for subjects receiving lenalidomide who are FCBP

2. The Lenalidomide Education and Counseling Guidance Document for each gender (female and male; Section 2 and Section 3 respectively) must be completed and signed by a trained counselor at the participating clinical center prior to each dispensing of lenalidomide. A copy of this document must be maintained in the subject's records for each dispense.

3. The Lenalidomide Information Sheet (Section 4) will be given to each subject receiving lenalidomide. The subject must read this document prior to starting lenalidomide and each time the subject receives a new supply of lenalidomide.

Lenalidomide Risks of Fetal Exposure, Pregnancy Testing Guidelines and Acceptable Birth Control Methods

1.1. Risks Associated with Pregnancy

Lenalidomide is structurally related to thalidomide. Thalidomide is a known human teratogenic active substance that causes severe life-threatening birth defects. An embryofetal development study in animals indicates that lenalidomide produced malformations in the offspring of female monkeys who received the drug during pregnancy. A teratogenic effect of lenalidomide in humans cannot be ruled out. Therefore, a pregnancy prevention program must be followed.

1.1.1. Definition of Females of Childbearing Potential

A FCBP is a female who: 1) has achieved menarche at some point, 2) has not undergone a hysterectomy or bilateral oophorectomy or 3) has not been naturally postmenopausal (amenorrhea following cancer therapy does not rule out childbearing potential) for at least 24 consecutive months (i.e., has had menses at any time in the preceding 24 consecutive months).

1.1.2. Definition of Females Not of Childbearing Potential

Females who do not meet the above definition of FCBP should be classified as FNCBP.

1.2. Counseling

1.2.1. Females of Childbearing Potential

For a FCBP, lenalidomide is contraindicated unless all of the following are met (i.e., all FCBP must be counseled concerning the following risks and requirements prior to the start of lenalidomide):

- She understands the potential teratogenic risk to the unborn child
- She understands the need for effective contraception, without interruption, 28 days before starting lenalidomide, throughout the entire duration of lenalidomide, during dose interruptions and for at least 28 days after the last dose of lenalidomide
- She understands and agrees to inform the Investigator if a change or stop of method of contraception is needed
- She must be capable of complying with effective contraceptive measures
- She is informed and understands the potential consequences of pregnancy and the need to notify her study doctor immediately if there is a risk of pregnancy
- She understands the need to commence lenalidomide as soon as it is dispensed following a negative pregnancy test

- She understands and accepts the need to undergo pregnancy testing based on the frequency outlined in this plan (Section 1.4) and in the Informed Consent
- She acknowledges that she understands the hazards lenalidomide can cause to an unborn fetus and the necessary precautions associated with the use of lenalidomide.

The Investigator must ensure that a FCBP:

- Complies with the conditions of the pregnancy prevention plan, including confirmation that she has an adequate level of understanding
- Acknowledges the aforementioned requirements.

1.2.2. Females Not of Childbearing Potential

For a FNCBP, lenalidomide is contraindicated unless all of the following are met (i.e., all FNCBP must be counseled concerning the following risks and requirements prior to the start of lenalidomide):

- She acknowledges she understands the hazards lenalidomide can cause to an unborn fetus and the necessary precautions associated with the use of lenalidomide.

1.2.3. Males

Traces of lenalidomide have been found in semen. Male subjects taking lenalidomide must meet the following conditions (i.e., all males must be counseled concerning the following risks and requirements prior to the start of lenalidomide):

- Understand the potential teratogenic risk if engaged in sexual activity with a pregnant female or a FCBP
- Understand the need for the use of a condom even if he has had a vasectomy, if engaged in sexual activity with a pregnant female or a FCBP
- Understand the potential teratogenic risk if the subject donates semen or sperm.

1.3. Contraception

1.3.1. Female Subjects of Childbearing Potential

Females of childbearing potential enrolled in this protocol must agree to use two reliable forms of contraception simultaneously or to practice complete abstinence (True abstinence is acceptable when this is in line with the preferred and usual lifestyle of the subject. Periodic abstinence [e.g. calendar, ovulation, symptothermal or post-ovulation methods] and withdrawal are not acceptable methods of contraception.) from heterosexual contact during the following time periods related to this study: 1) for at least 28 days before starting lenalidomide; 2) while taking lenalidomide; 3) during dose interruptions; and 4) for at least 28 days after the last dose of lenalidomide.

The two methods of reliable contraception must include one highly effective method and one additional effective (barrier) method. If the below contraception methods are not appropriate for the FCBP, she must be referred to a qualified provider of contraception methods to determine the medically effective contraception method appropriate to the subject. The following are examples of highly effective and additional effective methods of contraception:

- Examples of highly effective methods:

– Intrauterine device (IUD)

– Hormonal (birth control pills, injections, implants, levonorgestrel-releasing intrauterine system [IUS], medroxyprogesterone acetate depot injections, ovulation inhibitory progesterone-only pills [e.g. desogestrel])

– Tubal ligation

– Partner's vasectomy

- Examples of additional effective methods:

– Male condom

– Diaphragm

– Cervical Cap

Because of the increased risk of venous thromboembolism in subjects with multiple myeloma taking lenalidomide and dexamethasone, combined oral contraceptive pills are not recommended. If a subject is currently using combined oral contraception the subject should switch to another one of the highly effective methods listed above. The risk of venous thromboembolism continues for 4 to 6 weeks after discontinuing combined oral contraception. The efficacy of contraceptive steroids may be reduced during co-treatment with dexamethasone.

Implants and levonorgestrel-releasing intrauterine systems are associated with an increased risk of infection at the time of insertion and irregular vaginal bleeding. Prophylactic antibiotics should be considered particularly in subjects with neutropenia.

1.3.2. Male Subjects

Male subjects must practice complete abstinence (True abstinence is acceptable when this is in line with the preferred and usual lifestyle of the subject. Periodic abstinence [e.g. calendar, ovulation, symptothermal or post-ovulation methods] and withdrawal are not acceptable methods of contraception.) or agree to use a condom during sexual contact with a pregnant female or a FCBP while taking lenalidomide, during dose interruptions and for at least 28 days after the last dose of lenalidomide, even if he has undergone a successful vasectomy.

1.4. Pregnancy Testing

Medically supervised pregnancy tests with a minimum sensitivity of 25 mIU/mL must be performed for FCBP.

Females of childbearing potential must have two negative pregnancy tests (sensitivity of at least 25 mIU/mL) prior to starting lenalidomide. The first pregnancy test must be performed within 10 to 14 days prior to the start of lenalidomide and the second pregnancy test must be performed within 24 hours prior to the start of lenalidomide. The subject may not receive lenalidomide until the study doctor has verified that the results of these pregnancy tests are negative.

Females of childbearing potential with regular or no menstrual cycles must agree to have pregnancy tests weekly for the first 28 days of study participation and then every 28 days while taking lenalidomide, at study discontinuation, and at Day 28 following the last dose of lenalidomide.

Females of childbearing potential with irregular menstrual cycles must agree to have pregnancy tests weekly for the first 28 days of study participation and then every 14 days while taking lenalidomide, at study discontinuation, and at Days 14 and 28 following the last dose of lenalidomide.

1.5. Pregnancy Precautions for Lenalidomide Use

1.5.1. Before Starting Lenalidomide

1.5.1.1. Female Subjects of Childbearing Potential

Females of childbearing potential must have two negative pregnancy tests (sensitivity of at least 25 mIU/mL) prior to starting lenalidomide. The first pregnancy test must be performed within 10 to 14 days prior to the start of lenalidomide and the second pregnancy test must be performed within 24 hours prior to the start of lenalidomide. The subject may not receive lenalidomide until the study doctor has verified that the results of these pregnancy tests are negative.

Females of childbearing potential must use two reliable forms of contraception simultaneously, or practice complete abstinence (True abstinence is acceptable when this is in line with the preferred and usual lifestyle of the subject. Periodic abstinence [e.g. calendar, ovulation, symptothermal or post-ovulation methods] and withdrawal are not acceptable methods of contraception.) from heterosexual contact for at least 28 days before starting lenalidomide.

1.5.1.2. Male Subjects

Male subjects must agree to practice complete abstinence (True abstinence is acceptable when this is in line with the preferred and usual lifestyle of the subject. Periodic abstinence [e.g. calendar, ovulation, symptothermal or post-ovulation methods] and withdrawal are not acceptable methods of contraception.) or agree to use a condom during sexual contact with a pregnant female or a FCBP while taking

lenalidomide, during dose interruptions and for at least 28 days after the last dose of lenalidomide, even if he has undergone a successful vasectomy.

1.5.2. During and After Study Participation

1.5.2.1. Female Subjects

- Females of childbearing potential with regular or no menstrual cycles must agree to have pregnancy tests weekly for the first 28 days of study participation and then every 28 days while taking lenalidomide, at study discontinuation, and at Day 28 following the last dose of lenalidomide.
- Females of childbearing potential with irregular menstrual cycles must agree to have pregnancy tests weekly for the first 28 days of study participation and then every 14 days while taking lenalidomide, at study discontinuation, and at Days 14 and 28 following the last dose of lenalidomide.
- At each visit, the Investigator must confirm with the FCBP that she is continuing to use two reliable methods of birth control if not committing to complete abstinence, or confirm commitment to complete abstinence.
- If a FCBP considers the need to change or to stop a method of contraception, the Investigator must be notified immediately.
- Counseling about pregnancy precautions and the potential risks of fetal exposure must be conducted at a minimum of every 28 days.
- If pregnancy or a positive pregnancy test does occur in a subject, lenalidomide must be immediately discontinued.
- Pregnancy testing and counseling must be performed if a subject misses her period or if her pregnancy test or her menstrual bleeding is abnormal. Lenalidomide must be discontinued during this evaluation.
- Females must agree to abstain from breastfeeding while taking lenalidomide and for at least 28 days after the last dose of lenalidomide.

1.5.2.2. Male Subjects

- Must practice complete abstinence (True abstinence is acceptable when this is in line with the preferred and usual lifestyle of the subject. Periodic abstinence [e.g. calendar, ovulation, symptothermal or post-ovulation methods] and withdrawal are not acceptable methods of contraception.) or use a condom during sexual contact with a pregnant female or a FCBP while taking lenalidomide, during dose interruptions and for at least 28 days after the last dose of lenalidomide, even if he has undergone a successful vasectomy.
- Must not donate semen or sperm while receiving lenalidomide, during dose interruptions or for at least 28 days after the last dose of lenalidomide.
- Counseling about pregnancy precautions and the potential risks of fetal exposure must be conducted at a minimum of every 28 days.

- If pregnancy or a positive pregnancy test does occur in the partner of a male subject while taking lenalidomide, the Investigator must be notified immediately.

1.5.3. Additional Precautions

- Subjects should be instructed to never give lenalidomide to another person.
- Subjects should be instructed to return any unused capsules to the study doctor.
- Subjects should not donate blood while receiving lenalidomide, during dose interruptions and for at least 28 days after the last dose of lenalidomide.
- No more than a 28-day lenalidomide supply may be dispensed with each cycle of lenalidomide.

2. LENALIDOMIDE EDUCATION AND COUNSELING GUIDANCE DOCUMENT FOR FEMALE SUBJECTS

To be completed prior to each dispensing of lenalidomide.

Protocol Number: ____

Subject Name (Print): __________________ DOB: _____/_____/_____ (dd/mmm/yyyy)

Check one risk category:

☐ FCBP (Female of childbearing potential): a female who: 1) has achieved menarche (first menstrual cycle) at some point, 2) has not undergone a hysterectomy (the surgical removal of the uterus) or bilateral oophorectomy (the surgical removal of both ovaries) or 3) has not been naturally postmenopausal (amenorrhea following cancer therapy does not rule out childbearing potential) for at least 24 consecutive months (i.e., has had menses at any time during the preceding 24 consecutive months)

☐ NOT FCBP

2.1. Female of Childbearing Potential:

1. I have verified and counseled the subject regarding the following:

☐ Potential risk of fetal exposure to lenalidomide: A teratogenic potential of lenalidomide in humans cannot be ruled out. If lenalidomide is taken during pregnancy, it may cause birth defects or death to any unborn baby. Females are advised to avoid pregnancy while taking lenalidomide. Females of childbearing potential must agree not to become pregnant while taking lenalidomide.

☐ That the required pregnancy tests performed are negative.

☐ The subject confirmed that she is using TWO reliable methods of birth control at the same time, or complete abstinence (True abstinence is acceptable when this is in line with the preferred and usual lifestyle of the subject. Periodic abstinence [e.g. calendar, ovulation, symptothermal or post-ovulation methods] and withdrawal are not acceptable methods of contraception.) from heterosexual contact (at least 28 days prior to receiving lenalidomide, while receiving lenalidomide, during dose interruptions and for at least 28 days after the last dose of lenalidomide).

One highly effective method and one additional method of birth control must be used AT THE SAME TIME. The following are examples of highly effective and additional effective methods of contraception:

– Examples of highly effective methods:

- o Intrauterine device (IUD)
- o Hormonal (birth control pills, injections, implants, levonorgestrel-releasing intrauterine system [IUS], medroxyprogesterone acetate depot injections, ovulation inhibitory progesterone-only pills [e.g. desogestrel])
- o Tubal ligation

- o Partner’s vasectomy

  – Examples of additional effective methods:

- o Male condom
- o Diaphragm
- o Cervical Cap

☐ The subject confirmed that even if she has amenorrhea she must comply with advice on contraception.

☐ Pregnancy tests before, during administration of lenalidomide and at the last dose of lenalidomide, even if the subject agrees not to have reproductive heterosexual contact.

☐ Frequency of pregnancy tests to be done:

– Two pregnancy tests will be performed prior to receiving lenalidomide, one within 10 to 14 days, and a second within 24 hours of the start of lenalidomide.

– Every week during the first 28 days of this study and a pregnancy test every 28 days while the subject is taking lenalidomide if menstrual cycles are regular.

– Every week during the first 28 days of this study and a pregnancy test every 14 days while the subject is taking lenalidomide if menstrual cycles are irregular.

– If the subject missed a period or has unusual menstrual bleeding.

– When the subject is discontinued from the study and at Day 28 after the last dose of lenalidomide if menstrual cycles are regular. If menstrual cycles are irregular, pregnancy tests will be done at discontinuation from the study and at Days 14 and 28 after the last dose of lenalidomide.

☐ The subject confirmed that she will stop taking lenalidomide immediately in the event of becoming pregnant and to call her study doctor as soon as possible.

☐ The subject confirmed that she has not and will not breastfeed a baby while taking lenalidomide and for at least 28 days after the last dose of lenalidomide.

☐ The subject has not and will never share lenalidomide with anyone else.

☐ The subject has not and will not donate blood while taking lenalidomide, during dose interruptions and for at least 28 days after the last dose of lenalidomide.

☐ The subject has not and will not break, chew, or open lenalidomide capsules at any point.

☐ The subject confirmed that she will return unused lenalidomide capsules to the study doctor.

2. I have provided the Lenalidomide Information Sheet to the subject.

2.2. Female Not of Childbearing Potential (Natural Menopause for at Least 24 Consecutive Months, a Hysterectomy, or Bilateral Oophorectomy):

1. I have verified and counseled the subject regarding the following:

- ☐ Potential risk of fetal exposure to lenalidomide: A teratogenic potential of lenalidomide in humans cannot be ruled out. If lenalidomide is taken during pregnancy, it may cause birth defects or death to any unborn baby.
- ☐ The subject has not and will never share lenalidomide with anyone else.
- ☐ The subject has not and will not donate blood while taking lenalidomide, during dose interruptions and for at least 28 days after the last dose of lenalidomide.
- ☐ The subject has not and will not break, chew, or open lenalidomide capsules at any point.
- ☐ The subject confirmed that she will return unused lenalidomide capsules to the study doctor.

2. I have provided the Lenalidomide Information Sheet to the subject.

<u>**Do Not Dispense Lenalidomide if:**</u>

- **The subject is pregnant.**
- **No pregnancy tests were conducted for a FCBP.**
- **The subject states she did not use TWO reliable methods of birth control (unless practicing complete abstinence from heterosexual contact) at least 28 days prior to receiving lenalidomide, while receiving lenalidomide and during dose interruptions.**
- **The subject stated that she has or does not want to adhere to pregnancy precautions outlined within this PPP.**

Counselor Name (Print): ____________________

Counselor Signature: _______________________ Date: _____/_____/_____(dd/mmm/yyyy)

Maintain a copy of the Education and Counseling Guidance Document in the subject's records.

3. LENALIDOMIDE EDUCATION AND COUNSELING GUIDANCE DOCUMENT FOR MALE SUBJECTS

To be completed prior to each dispensing of lenalidomide.

Protocol Number: ____

Subject Name (Print): _________________ DOB: _____/_____/_____ (dd/mmm/yyyy)

1. I have verified and counseled the subject regarding the following:

- ☐ Potential risk of fetal exposure to lenalidomide: A teratogenic potential of lenalidomide in humans cannot be ruled out. If lenalidomide is taken during pregnancy, it may cause birth defects or death to any unborn baby.
- ☐ The subject confirmed that he has practiced complete abstinence (True abstinence is acceptable when this is in line with the preferred and usual lifestyle of the subject. Periodic abstinence [e.g. calendar, ovulation, symptothermal or post-ovulation methods] and withdrawal are not acceptable methods of contraception.) or used a condom when engaging in sexual contact (including those who have had a vasectomy) with a pregnant female or FCBP, while taking lenalidomide, during dose interruptions and for at least 28 days after the last dose of lenalidomide.
- ☐ The subject confirmed that he has not impregnated his female partner while in the study.
- ☐ The subject confirmed that he will notify his study doctor if his female partner becomes pregnant and the female partner of a male subject taking lenalidomide confirmed that she will call her healthcare provider immediately if she becomes pregnant.
- ☐ The subject has not and will never share lenalidomide with anyone else.
- ☐ The subject confirmed that he has not donated and will not donate semen or sperm while taking lenalidomide or during dose interruptions and that he will not donate semen or sperm for at least 28 days after the last dose of lenalidomide.
- ☐ The subject has not and will not donate blood while taking lenalidomide, during dose interruptions and for at least 28 days after the last dose of lenalidomide.
- ☐ The subject has not and will not break, chew, or open lenalidomide capsules at any point.
- ☐ The subject confirmed that he will return unused lenalidomide capsules to the study doctor.

2. I have provided the Lenalidomide Information Sheet to the subject.

<u>Do Not Dispense Lenalidomide if:</u>

- **The subject stated that he has or does not want to adhere to pregnancy precautions outlined within this PPP.**

Counselor Name (Print): ____________________

Counselor Signature: _______________________ Date: _____/_____/_____(dd/mmm/yyyy)

Maintain a copy of the Education and Counseling Guidance Document in the subject's records.

4. LENALIDOMIDE INFORMATION SHEET

For subjects enrolled in clinical research studies

Please read this Lenalidomide Information Sheet before you start taking lenalidomide and each time you get a new supply. This Lenalidomide Information Sheet does not take the place of an informed consent to participate in clinical research or talking to your study doctor or healthcare provider about your medical condition or your treatment.

What is the most important information I should know about lenalidomide?

1. **Lenalidomide may cause birth defects (deformed babies) or death of an unborn baby.** Lenalidomide is similar to the medicine thalidomide. It is known that thalidomide causes life-threatening birth defects.

If you are a female who is able to become pregnant:

- **Do not take lenalidomide if you are pregnant or plan to become pregnant**
- **You must practice complete abstinence from sexual contact with a male or use two reliable, separate forms of effective birth control at the same time:**

– for 28 days before starting lenalidomide

– while taking lenalidomide

– during breaks (dose interruptions) of lenalidomide

– for at least 28 days after the last dose of lenalidomide

- **You must have pregnancy testing done at the following times:**

– within 10 to 14 days prior to the first dose of lenalidomide

– 24 hours prior to the first dose of lenalidomide

– weekly for the first 28 days

– if you have regular menstrual periods: every 28 days after the first month

– if you have irregular menstrual periods: every 14 days after the first month

– if you miss your period or have unusual menstrual bleeding

– 28 days after the last dose of lenalidomide (14 and 28 days after the last dose if menstrual periods are irregular)

- **Stop taking lenalidomide if you become pregnant while taking lenalidomide**

– If you suspect you are pregnant at any time during the study, you must stop lenalidomide immediately and immediately inform your study doctor. Your study doctor will report all cases of pregnancy to Celgene Corporation.

- **Do not breastfeed while taking lenalidomide and for at least 28 days after the last dose of lenalidomide**

- The study doctor will be able to advise you where to get additional advice on contraception.

If you are a female not able to become pregnant:

In order to ensure that an unborn baby is not exposed to lenalidomide, your study doctor will confirm that you are not able to become pregnant.

If you are a male:

A small amount of lenalidomide is found in human semen. The risk to an unborn baby in females whose male partner is receiving lenalidomide is unknown at this time.

- Male subjects (including those who have had a vasectomy) must practice complete abstinence or must use a condom during sexual contact with a pregnant female or a female that can become pregnant:

– While you are taking lenalidomide

– During breaks (dose interruptions) of lenalidomide

– For at least 28 days after the last dose of lenalidomide

- **Male subjects should not donate sperm or semen** while taking lenalidomide, during breaks (dose interruptions) and for at least 28 days after the last dose of lenalidomide.

- **If you suspect that your partner is pregnant any time during the study, you must immediately inform your study doctor. The study doctor will report all cases of pregnancy to Celgene Corporation. Your partner should call their healthcare provider immediately if they become pregnant.**

2. **All subjects:**

- **Do not share lenalidomide with other people. It must be kept out of the reach of children and should never be given to any other person.**

- **Do not donate blood** while you take lenalidomide, during breaks (dose interruptions) and for at least 28 days after the last dose of lenalidomide.

- **Do not break, chew, or open lenalidomide capsules at any point.**

- You will get no more than a 28-day supply of lenalidomide at one time.

- Return unused lenalidomide capsules to your study doctor.

Additional information is provided in the informed consent form and you can ask your study doctor for more information.

APPENDIX 8: SCHEDULE OF EVENTS

PROCEDURES	Screen	KRd + Dara Induction Cycles 1-4[38]							
DAY	-21 to -1	1	2	8	9	15	16	22	End of Cycle 4
Informed Consent	X								
Medical/Treatment History[1]	X								
Skeletal Survey[2]	X								
ECG[3]	X								
Physical Exam[5]	X	X[4]							
Vital Signs[6]	X	X	X	X	X	X	X		X
Height, Weight, BSA	X	X							
24-hour urine[7]	X	X[4,7]				X[7]			X[7]
Urinalysis	X								
CRP	X								

PROCEDURES	Screen	KRd + Dara Induction Cycles 1-4[38]							
**DAY**	**-21 to -1**	**1**	**2**	**8**	**9**	**15**	**16**	**22**	**End of Cycle 4**
Hematology[9]	X	X		X[10]		X[10]			
Serum Chemistry	X[11]	X[11]	(X)[10, 12]	X[11]	(X)[10, 12]	X[11]	(X)[10, 12]		
Pregnancy Test[13]	X	X		X[14]		X[14]		X[14]	
Hepatitis B (HBV) serology[34]	X								
HBV DNA testing	X								X[35]
**Disease Assessment**									X[15]
β2-microglobulin	X								
SPEP, UPEP[16]	X	X[4]				X[17]			X
Immunofixation – Serum and Urine	X	X[4]				X[17]			X
BM Aspirate/biopsy, cytogenetics, FISH[18]	X								X[18]
Plasmacytoma Evaluation[31]	X								
Quantitative Igs	X	X[4]				X[17]			X

PROCEDURES	Screen	KRd + Dara Induction Cycles 1-4[38]							
DAY	-21 to -1	1	2	8	9	15	16	22	End of Cycle 4
SFLC[19]	X								X[19]
Neurological Assessment[20]	X	X[20]							
Correlative Samples[21]	X								X[21]
Bone marrow aspirate sample for MRD analysis[32]	X								X
CT-PET[33]	X								X
Adverse Events[22]		Ongoing							
Concomitant Medications		Ongoing							
Register Patient into Revlimid REMS® program and prescribe lenalidomide[24]	X								
Daratumumab		X		C1-2		X			
Carfilzomib[25]		X	X	X	X	X	X		
Revlimid® (Lenalidomide)[26]		X	X	X	X	X	X		

**PROCEDURES**	**Screen**	**KRd + Dara Induction Cycles 1-4**[38]							
**DAY**	**-21 to -1**	**1**	**2**	**8**	**9**	**15**	**16**	**22**	**End of Cycle 4**
Dexamethasone[27]		X	X[27]	X	X[27]	X	X[27]	X	

***Following cycle 4, subjects will proceed to Stem Cell Collection with potential for deferred ASCT**

**PROCEDURES**	**KRd + Dara Consolidation Cycles 5-8**								**KRd + Dara Maintenance Cycles 9-24**						**End of Tx**	**LTFU**
**DAY**	**1**	**2**	**8**	**9**	**15**	**16**	**22**	**End of Cycle 8**[30]	**1**	**2**	**8**	**15**	**16**	**22**		
Skeletal Survey															X	
ECG[3]															X	
Physical Exam[5]	X								X						X	

PROCEDURES	KRd + Dara Consolidation Cycles 5-8								KRd + Dara Maintenance Cycles 9-24						End of Tx	LTFU
**DAY**	**1**	**2**	**8**	**9**	**15**	**16**	**22**	**End of Cycle 8[30]**	**1**	**2**	**8**	**15**	**16**	**22**		
Vital Signs	X	X	X	X	X	X			X	X		X	X		X	
Height, Weight, BSA	X								X						X	
24-hour urine[7]	X[7]								X[7]						X[7]	
Urinalysis																
Hematology[9]	X		X		X				X			X			X	
Serum Chemistry	X[11]		X[11]		X[11]			X[11]	X[11]						X	
Pregnancy Test[13]	X				X[14]				X[14]			X[14]			X[14]	
HBV DNA testing[35]	Q12W during treatment														X	X[36]
**Disease Assessment**								X[15]						X[15]		X[23]
β2-microglobulin								X								
SPEP, UPEP[16]	X							X	X						X	
Immunofixation – Serum and Urine	X							X	X						X	

PROCEDURES	KRd + Dara Consolidation Cycles 5-8								KRd + Dara Maintenance Cycles 9-24						End of Tx	LTFU
**DAY**	**1**	**2**	**8**	**9**	**15**	**16**	**22**	**End of Cycle 8[30]**	**1**	**2**	**8**	**15**	**16**	**22**		
BM Aspirate/biopsy, cytogenetics, FISH[18]								X[18]							X[18]	X
Quantitative Igs	X							X	X						X	
SFLC[19]								X[19]							X[19]	
Neurological Assessment[20]	X								X						X	
Correlative Samples[21]															X[21]	
Bone marrow aspirate sample for MRD analysis[32]	X							X							X	X[32]
CT-PET[33]	X								X						X	X[33]
Adverse Events[22]	Ongoing															
Concomitant Medications	Ongoing															
Daratumumab[37]	X				X				X							

PROCEDURES	KRd + Dara Consolidation Cycles 5-8								KRd + Dara Maintenance Cycles 9-24						End of Tx	LTFU
**DAY**	**1**	**2**	**8**	**9**	**15**	**16**	**22**	**End of Cycle 8[30]**	**1**	**2**	**8**	**15**	**16**	**22**		
Carfilzomib[25]	X	X	X	X	X	X			X	X		X	X			
Revlimid® (Lenalidomide)[26,28]	X	X	X	X	X	X			X	X	X	X	X	X		X[28]
Dexamethasone[27]	X		X		X		X		X		X	X		X		
Survival and New Cancer Evaluation [29]																X

* Variations of ± 3 days of the scheduled visit are permitted however doses of carfilzomib may only be rescheduled by up to 2 days (Section 6.3.1).

**The KRd + Dara consolidation cycles are Cycle 5 – 8. Maintenance Cycles are 9-24.

Footnotes for Appendix 8

\+ Documented informed consent must be obtained within ≤ 30 days prior to initiation of therapy.

1. Includes neuropathy history.
2. May be within 30 days planned treatment start. Includes: lateral radiograph of the skull, anteroposterior and lateral views of the spine, and anteroposterior views of the pelvis, ribs, femora, and humeri. Skeletal surveys performed outside of the 30 day window may be considered for inclusion. Please contact the Lead Principal Investigator and/or/CRA on a case-by-case basis. End of treatment skeletal survey only if clinically indicated.
3. 12-lead ECG, including QTc interval. End of treatment ECG only if clinically indicated.
4. For Day 1 of cycle 1, screening results may be used if within 7 days of treatment start.
5. Complete physical exam (including vital signs [systolic and diastolic blood pressure, respiration, pulse, oral temperature], height, weight, calculation of body surface area [BSA]) and ECOG score) required at screening and Day 1 of each cycle.

6. Systolic and diastolic blood pressure, pulse, respiration, temperature approximately 1 hour before dosing.
7. 24 hour urine total protein, urine protein electrophoresis (UPEP), and urine protein immunofixation. For subjects whose disease is being monitored through UPEP, additional post baseline 24-hour urine collections are required as indicated.
8. For subjects whose disease is being monitored through UPEP, additional post baseline 24-hour urine collections are required as indicated.
9. Hemoglobin, hematocrit, WBC with complete differential, RBCs, platelet count. Results must be reviewed before dosing.
10. Cycles 1 and 2 only and subsequent cycles as clinically indicated or per institutional standard of care.
11. Full serum chemistry panel at Screening, Days 1, 8, and 15 of Cycles 1-8, and Day 1 of Cycles 9+: sodium, potassium, chloride, bicarbonate, BUN, creatinine, glucose, uric acid, total protein, albumin, calcium, phosphorus, magnesium, total bilirubin, alkaline phosphatase, ALT, AST, LDH. Results must be reviewed before dosing in Cycles 1 and 2.
12. Abbreviated serum chemistry panel on Days 2, 9, and 16 of Cycles 1 and 2 only or as clinically indicated (e.g., risk factors for tumor lysis syndrome): sodium, potassium, chloride, bicarbonate, BUN, creatinine, glucose, uric acid.
13. Pregnancy tests for females of childbearing potential. A female of childbearing potential (FCBP) is a sexually mature female who: 1) has not had a hysterectomy or bilateral oophorectomy; or 2) has not been naturally post-menopausal for at least 24 consecutive months (i.e., menses within the preceding 24 months).
14. Pregnancy tests must occur within 10-14 days and again within 24 hours prior to prescribing lenalidomide (prescriptions must be filled within 7 days. FCBP with regular or no menstruation must have a pregnancy test weekly for the first 28 days and then every 28 days while on therapy (including breaks in therapy); at discontinuation of lenalidomide and at Day 28 post the last dose of lenalidomide. Females with irregular menstruation must have a pregnancy test weekly for the first 28 days and then every 14 days while on therapy (including breaks in therapy), at discontinuation of lenalidomide and at Day 14 and Day 28 post the last dose of lenalidomide (see Appendix 7 for Risks of Fetal Exposure, Pregnancy Testing Guidelines and Acceptable Birth Control Methods).
15. After Cycle 4, subjects who are deemed candidates for ASCT will proceed to stem cell harvest as per institutional guidelines. However, these subjects will not proceed straight to ASCT and will have cells frozen down for a potential future transplant at progression or unacceptable toxicity after stem cell harvest. After completion of stem cell harvest, subjects will resume protocol treatment.
16. Serum protein electrophoresis and urine protein electrophoresis (the latter only for those whose disease is being followed by UPEP). Subjects with baseline urine protein greater than 200 mg/24 hours must have a UPEP to confirm VGPR or better. Obtain blood for M-protein levels measured by SPEP or quantitative immunoglobulins for those subjects in whom SPEP/UPEP are felt to be unreliable (IgA type multiple myeloma), depending upon which studies were positive at baseline.
17. Disease assessments on Day 15 of Cycle 1 (only) are only for assessing onset of early response, not for definitive achievement of response.
18. Bone marrow aspirate and biopsy - quantify % myeloma cell involvement; bone marrow sample for cytogenetics and fluorescent in situ hybridization (FISH). Bone marrow aspirate and biopsy should be performed at screening and the end of D-KRD consolidation (end of cycle 8), the end of D-KRD treatment (end of cycle 24) and then yearly for up to 2 years. See sections 6.3-6.5. Repeat bone marrow biopsy/aspirate if CR is suspected and as appropriate to confirm achievement of sCR, CR, or nCR (aspirate only—biopsy not required). If CR is suspected after cycle 8, the bone marrow biopsy/aspirate must be performed at the end of

cycle 12. Bone marrow biopsy/aspirate performed outside of the 30 day window may be considered for inclusion. Please contact the Lead Principal Investigator and/or the CRA on a case-by-case basis. (Cytogenetics is required at screening only. If cytogenetics is completed at a time point other than screening, the results should be captured in eCRF)

19. SFLC repeated only to confirm CR.
20. Screening and Day 1 of every cycle. Includes neurologic exam (to detect peripheral neuropathy and/or changes in pre-existing neuropathy) and examination of clinical AEs indicative of neuropathy. Collect FACT/GOG neurotoxicity questionnaire at each time point above.
21. Peripheral blood and bone marrow aspirate samples collected at screening, end of cycle 8, time of response (to confirm complete response), end-of-treatment visit, and yearly after end of treatment for up to 2 years. Buccal mucosa swab will be collected at screening only.
22. AEs will be collected from the time of signing informed consent. 30 days following the last dose of D-KRD, patients will be considered to be off-study so no toxicities will be recorded following this period. All concomitant medications must be recorded on the concomitant medications case report form from 21 days before Day 1 through 30 days following the last dose of study drugs.
23. Assessment for disease progression in subjects who did not progress during treatment. At least every 3 months (+/- 30 days) for 5 years from safety follow-up visit (which must be 28days (+/- 3 days) post-last study treatment).
24. Lenalidomide must be prescribed through and in compliance with the Revlimid REMS® program of Celgene Corporation. Prescriptions must be filled within 7 days. Consideration should be given to prescribing lenalidomide 5 to 7 days in advance of Day 1 of each cycle to allow time for required patient and prescriber surveys, and drug shipment to patient. Any unused Revlimid® (lenalidomide) should be returned to the patient for disposition in accordance with the Revlimid REMS® program.
25. Cycles 1-8: Days 1, 2, 8, 9, 15 & 16. Cycles 9-24 Days 1, 2, 15 and 16.
26. Daily on Days 1-21 followed by a 7-day rest period every 28 days. On day coinciding with carfilzomib administration, lenalidomide should be taken at least 4 hours after the carfilzomib dose and may be self-administered at home by the subject. On days that carfilzomib is not administered, lenalidomide should be taken at approximately the same time each day.
27. Days 1, 8, 15 and 22. Dexamethasone is given between 30 minutes and 4 hours before carfilzomib on days they coincide. Dexamethasone will be dosed at 40 mg PO per week during Cycles 1-4 and 20 mg PO per week during Cycles 5-24. Cycles 9-24 represent the D-KRD Maintenance Phase of therapy and the dose of dexamethasone will remain 20 mg on days 1, 8, 15 and 22. For weeks of daratumumab injection, dexamethasone dose is split into two doses: half the dose prior to daratumumab infusion and half the dose the day after. Split weekly dosing for all other days (e.g. 10 mg on Day 1 and 10 mg on Day 2, etc.) is permitted with approval from Lead Principal Investigator. Dexamethasone given on days without carfilzomib (on Days 22 and 23 of Cycles 1-8) may be self-administered by the subject on an outpatient basis. If Day 2 of carfilzomib dosing is delayed (i.e., Day 2, 9, 16) 4 mg of dexamethasone premedication is required to be used prior to second treatment. Missed doses will not be replaced during a cycle. Missed doses of dexamethasone will not be made up. Single-agent lenalidomide maintenance therapy, is recommended using last tolerated dose of lenalidomide for 21 days in 28 day cycles after D-KRD protocol treatment (Cycles 1-24) is completed.
28. Patients will be followed for survival and development of any new cancers, at least every 3 months. Reports of any death should include date of death and specific cause (disease under study or specify other cause).
29. Evaluations at the end of Cycle 8 may be done on Day 1 of the next Cycle 9.

30. Plasmacytoma evaluation is required at screening only and may be completed as physical exam or imaging if indicated and at the treating investigator's discretion. After screening, plasmacytoma evaluation is required only for patients who have no other measurable disease and/or at investigator's discretion.
31. One bone marrow sample for central Minimal Residual Disease (MRD) analysis by NGS at Adaptive Biotechnologies will be collected from all subjects: 1) screening (sample is required for calibration of MRD by gene sequencing In the event that a superfluous bone marrow aspirate –BMA- sample from screening was not available, slides of BMA smear or clot from a time prior to enrollment can be used), 2) end of cycle 8, 3) end of cycle 24 (EOT), 4) 1 and 2 years after EOT for all subjects; 5) any time that a bone marrow is performed as SOC to assess CR response. If CR is suspected after cycle 8, the bone marrow biopsy/aspirate must be performed at the end of Cycle 12. For any bone marrow collected after Cycle 8 or Cycle 24, the visit may be done on Day 22-28 of that cycle.
32. A CT-PET will be performed to confirm MRD-negative disease per Standard of Care, at every time-point when MRD is checked (+/- 2 weeks). A bone marrow done as SOC 1 and 2 years after the EOT BM will be tested for MRD and a CT-PET done to confirm MRD-negative disease (+/- 2 weeks).
33. Local testing for hepatitis B surface antigen (HBsAg), hepatitis B surface antibody (Anti-HBs), and hepatitis B core antibody (Anti-HBc). HBV serology is not required at Screening if this was performed as part of standard of care within 3 months prior to first dose.
34. For subjects with serologic evidence of resolved HBV infection (i.e., positive Anti-HBs or positive Anti-HBc) at Screening, HBV DNA testing by PCR must be performed locally.
35. Q12W for up to 6 months after the last dose of study treatment.
36. If administered by subcutaneous (SC) injection, daratumumab will be provided as a fixed-dose of 1800 mg and 30,000 units hyaluronidase per 15 mL (120 mg and 2,000 units/mL) for all cycles. All subjects receiving daratumumab subcutaneous injection will be observed for at least 6 hours after the end of the SC injection during Cycle 1 Day 1 and, if deemed necessary by the treating investigator, after subsequent injections.
37. No provider patient visits will be held on Cycle 1, Day 22.
