# Supplementary material for: Final analysis of a phase II trial of daratumumab, carfilzomib, lenalidomide, and dexamethasone in newly diagnosed multiple myeloma without transplant
Source: Blood Cancer J. 2024 May 29;14(1):87. doi: 10.1038/s41408-024-01045-3 (PMC11136961; doi:10.1038/s41408-024-01045-3)
Supplement: Supplementary file 2 — Supplemental material [file 41408_2024_1045_MOESM2_ESM.pdf]

Supplemental Appendix for "Final Analysis of a Phase II Trial of Daratumumab, Carfilzomib, Lenalidomide, and Dexamethasone in Newly Diagnosed Multiple Myeloma Without Transplant"

Table of Contents

Supplemental Table 1: Patient Disposition 2

Supplemental Table 2: Characteristics of Patients with Disease Progression 3

Supplemental Figure 1: MRD by NGS Status Using Intent-to-Treat Principle 4

Supplemental Figure 2: MRD by Mass Spectrometry Using Intent-to-Treat Principle 5

Supplemental Figure 3: Progression-Free Survival Stratified by Next Generation Sequencing Following Cycle 8 of Dara-KRd 6

Supplemental Figure 4: Progression-Free Survival Stratified by Mass Spectrometry Status Following Cycle 8 of Dara-KRd 7

Supplemental Table 1: Patient Disposition

Outcome	Enrolled Patients (n=42)
Reached end of therapy	21 (50%)
Receiving treatment per protocol	11 (26%)
Disease progression during treatment	6 (14%)
Discontinued protocol therapy early*	4 (10%)
Proceeded to ASCT off protocol	4 (10%)
In response	1 (2%)
After progression	3 (7%)
Post-protocol therapies of responders	
Dual maintenance	15 (36%)
Single-agent maintenance	6 (14%)
Discontinued all therapy at later point	6 (14%)
Disease Progression	7 (17%)
Death	2 (5%)

*1 due to geographic relocation, 1 due to psychological disturbance unrelated to treatment, 1 did not adhere to REMS program for lenalidomide, and 1 did not adhere to study protocol.

Supplemental Table 2: Characteristics of Patients with Disease Progression

Patient	Cytogenetics at Diagnosis	EMD at Diagnosis	MS MRD Best Response	NGS MRD Best Response	Time to progression
101-03	t(4;14)	No	Always positive	Always positive	41.2 months
101-13	t(4;14), 1q gain	Yes	Unknown (early progression)	Unknown (early progression)	0.8 months
101-25	t(4;14), 1q amplification, deletion 17p	Yes	Always positive	Unknown (no clone)	18.7 months
101-26	t(4;14), 1q amplification	No	EXENT® negative LC-MS positive	10^{-6} negative	18.1 months
101-31	t(4;14)	Yes	Always positive	10^{-5} negative 10^{-6} positive	14.1 months
101-35	Unknown cytogenetics; 15% circulating plasma cells	No	Unknown (early progression)	Unknown (early progression	1.2 months
101-40	t(4;14), 1q gain	Yes	Always positive	Unknown (no clone)	17.2 months

EMD = extramedullary disease; LC-MS = liquid chromatography mass spectrometry; MS = mass spectrometry

Supplemental Figure 1: MRD by NGS Status Using Intent-to-Treat Principle

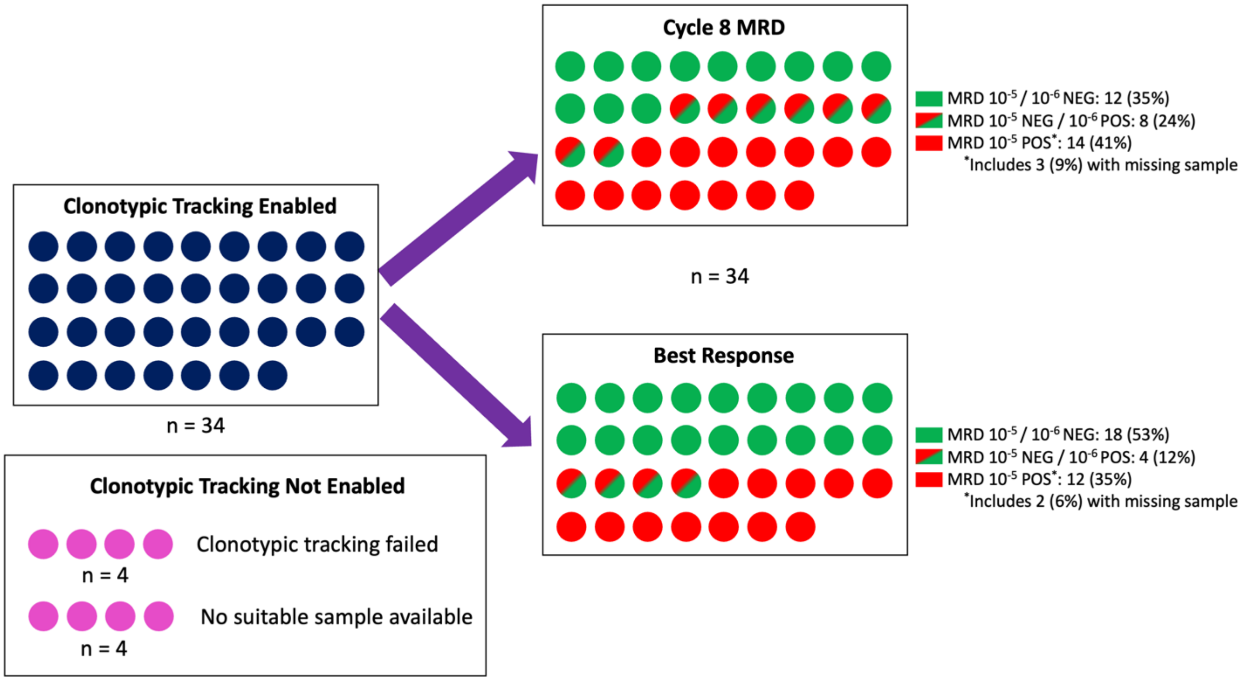

Supplemental Figure 2: MRD by Mass Spectrometry Using Intent-to-Treat Principle

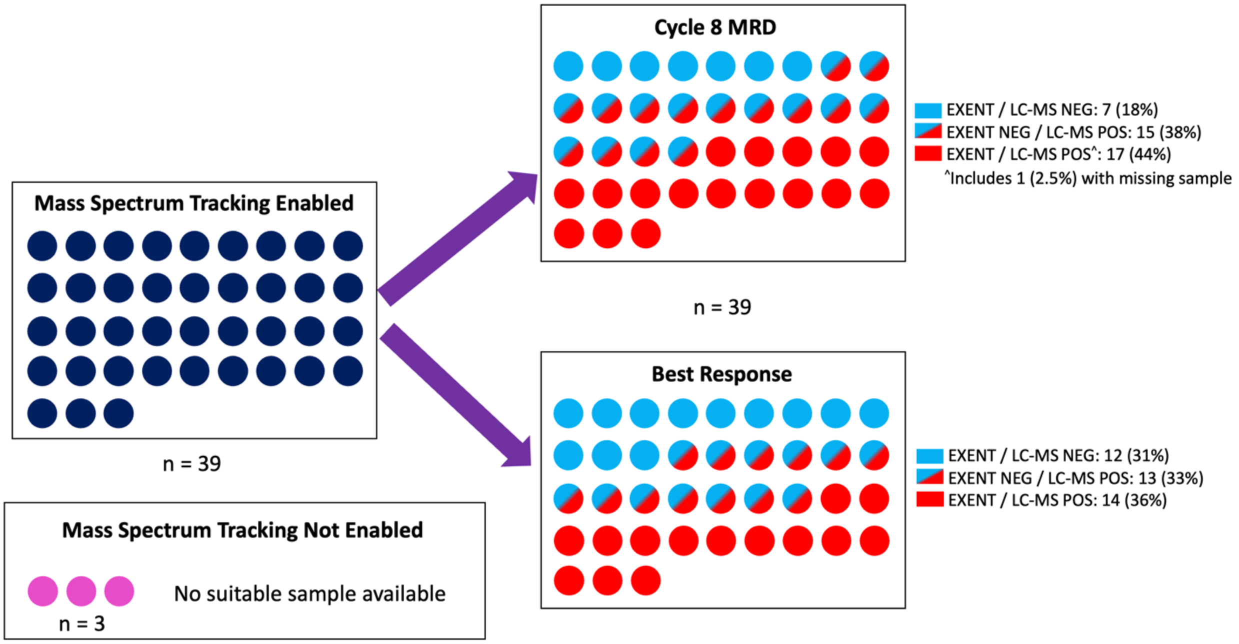

Supplemental Figure 3: Progression-Free Survival Stratified by Next Generation Sequencing Following Cycle 8 of Dara-KRd

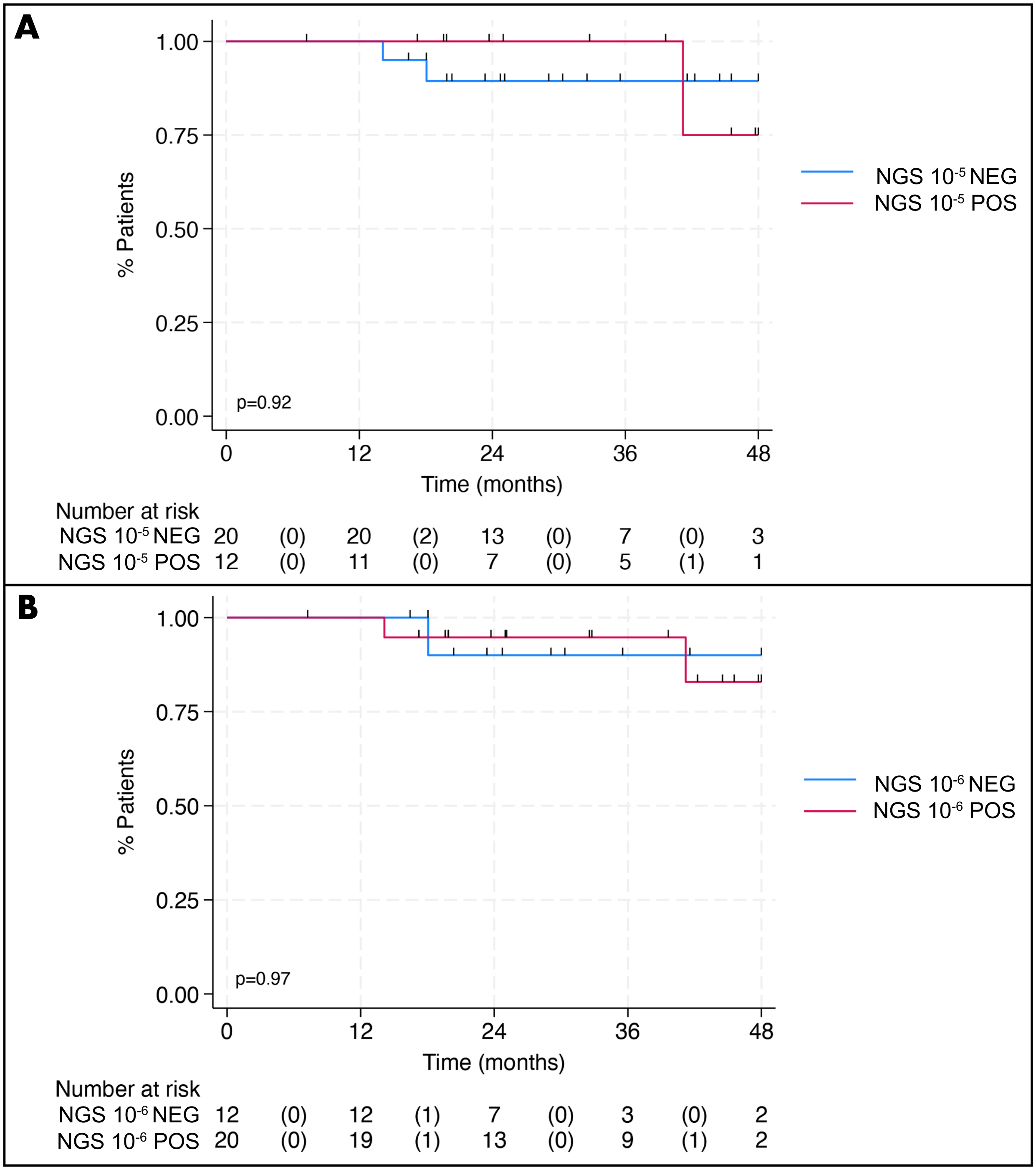

A) Progression-free survival stratified by next generation sequencing (NGS) 10^{-5} status after eight cycles of Dara-KRd using the landmark method. (B) Progression-free survival stratified by NGS 10^{-6} status after eight cycles of Dara-KRd using the landmark method.

Supplemental Figure 4: Progression-Free Survival Stratified by Mass Spectrometry Status Following Cycle 8 of Dara-KRd

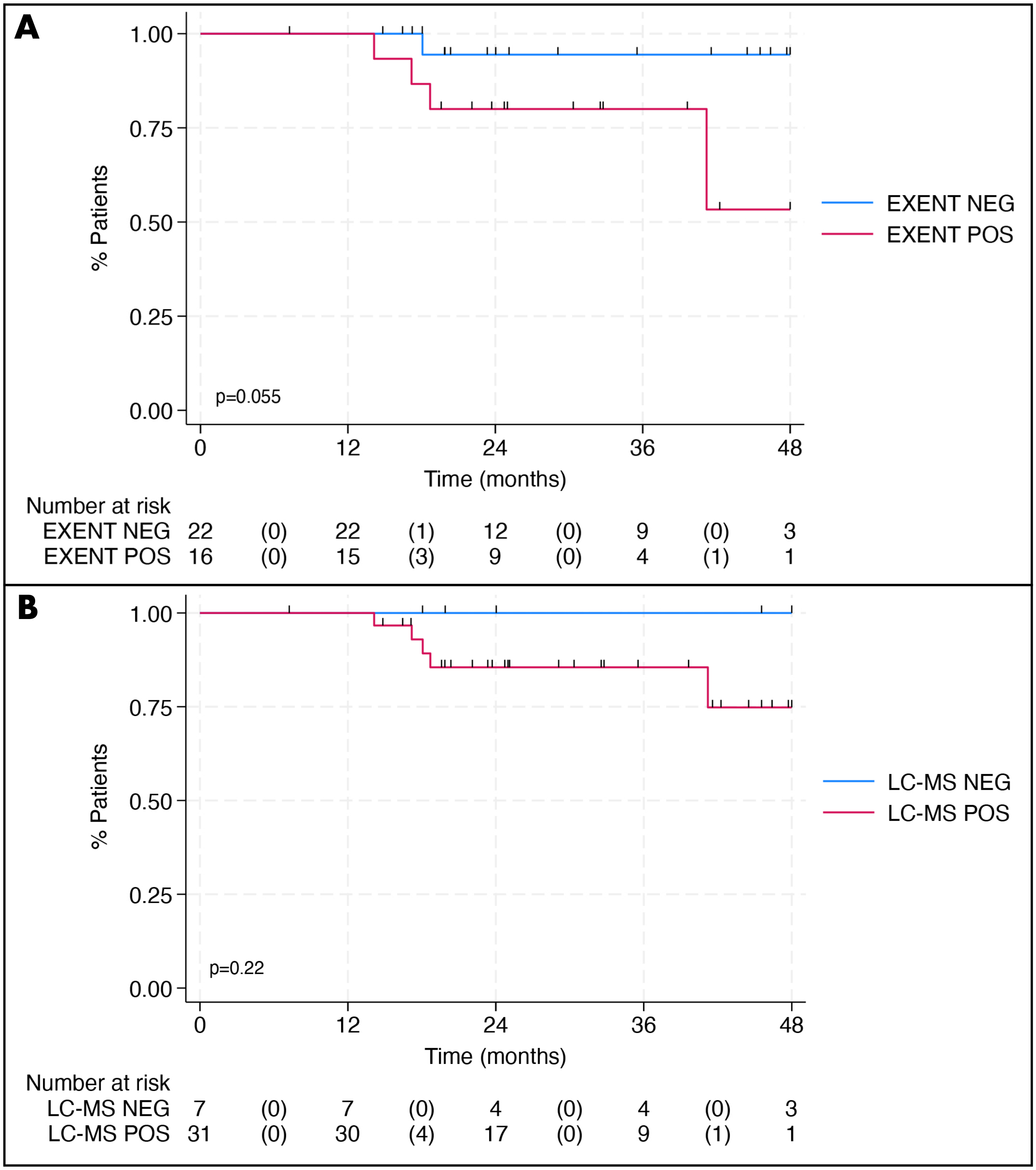

A) Progression-free survival stratified by EXENT status after eight cycles of Dara-KRd using the landmark method. (B) Progression-free survival stratified by liquid-chromatography mass spectrometry (LC-MS) after eight cycles of Dara-KRd using the landmark method.
